# Principles of melting in hybrid organic–inorganic perovskite and polymorphic ABX_3_ structures†

**DOI:** 10.1039/d1sc07080k

**Published:** 2022-01-20

**Authors:** Bikash Kumar Shaw, Celia Castillo-Blas, Michael F. Thorne, María Laura Ríos Gómez, Tom Forrest, Maria Diaz Lopez, Philip A. Chater, Lauren N. McHugh, David A. Keen, Thomas D. Bennett

**Affiliations:** Department of Materials Science and Metallurgy, University of Cambridge CB3 0FS UK tdb35@cam.ac.uk; Departamento de Química Inorgánica, Universidad Autónoma de Madrid 28049 Madrid Spain; Diamond Light Source Ltd, Diamond House, Harwell Campus Didcot Oxfordshire OX11 0DE UK; ISIS Facility, Rutherford Appleton Laboratory, Harwell Campus Didcot Oxfordshire OX11 0QX UK

## Abstract

Four novel dicyanamide-containing hybrid organic–inorganic ABX_3_ structures are reported, and the thermal behaviour of a series of nine perovskite and non-perovskite [AB(N(CN)_2_)_3_] (A = (C_3_H_7_)_4_N, (C_4_H_9_)_4_N, (C_5_H_11_)_4_N; B = Co, Fe, Mn) is analyzed. Structure–property relationships are investigated by varying both A-site organic and B-site transition metal cations. In particular, increasing the size of the A-site cation from (C_3_H_7_)_4_N → (C_4_H_9_)_4_N → (C_5_H_11_)_4_N was observed to result in a decrease in *T*_m_ through an increase in Δ*S*_f_. Consistent trends in *T*_m_ with metal replacement are observed with each A-site cation, with Co < Fe < Mn. The majority of the melts formed were found to recrystallise partially upon cooling, though glasses could be formed through a small degree of organic linker decomposition. Total scattering methods are used to provide a greater understanding of the melting mechanism.

## Abstract 2

Increasing the size of the A-site cation from (C_3_H_7_)_4_N → (C_4_H_9_)_4_N → (C_5_H_11_)_4_N in hybrid organic–inorganic ABX_3_ structures was observed to result in a decrease in *T*_m_, through an increase in Δ*S*_f_.

## Graphical abstract

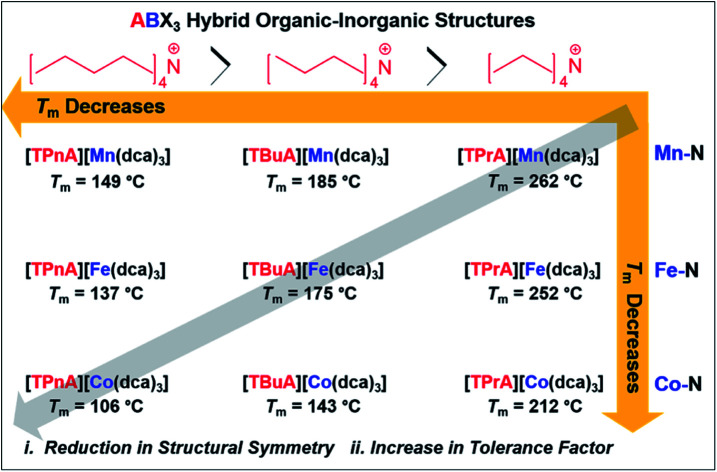

## Introduction

Hybrid organic–inorganic perovskites (HOIPs) are a huge class of crystalline materials with general formula ABX_3_, where A, B and X are an organic cation, metal cation and multidentate inorganic or organic anion, respectively. HOIPs have recently emerged as efficient optoelectronic materials thanks to their valuable utility in ionic transport,^1^ multiferroic^2^ and photovoltaic applications.^3^ They possess a high chemical and structural variability through the replacement of the X species with other multidentate bridging ligands such as cyanide [CN^−^], thiocyanate [SCN^−^], formate [HCOO^−^], hypophosphite [H_2_POO^−^] or dicyanamide [dca, N(CN)_2_^−^], among others.^4–7^ Careful selection, or modification of the linker and/or metal cation allows property modulation and enhancement.^8^

The extended hybrid connectivity of HOIPs mean that they share many similarities with metal–organic frameworks (MOFs).^9^ However, HOIPs are considerably more dense than MOFs given the presence of the A-site cation, which means they are typically unable to selectively adsorb guest species.^10^ Accordingly, interest in their behaviour does not revolve around porosity, but instead concentrates on ionic conductivity,^11^ ferroelectric^12^ and barocaloric^13^ properties.

Phase transitions between crystalline HOIP polymorphs upon application of external stimuli such as temperature or pressure are relatively common. Such transitions are commonly associated with, amongst other factors, displacement of the organic A site cation, and a large anisotropic thermal expansion.^14^ For example, [TPrA][Mn(dca)_3_] (TPrA = tetrapropylammonium, [(CH_3_CH_2_CH_2_)_4_N]^+^), displays a first-order structural phase transition at 57 °C from a non-centrosymmetric structure (space group *P*4̄2_1_*c*), to a high temperature polymorph with the centrosymmetric space group *I*4/*mcm*.^15^

Despite the dominance of the solid crystalline state in the HOIPs family due to their thermal stability,^16,17^ several two-dimensional HOIPs have previously demonstrated the ability to melt into liquid phases. These 2D perovskites, known as Ruddlesden–Popper phases, contain inorganic perovskite layers separated by cations. Work on lead iodide alkylammonium derivatives, *i.e.* those with the general formula (RNH_3_)_2_MA_*n*−1_Pb_*n*_I_3*n*+1_, has shown melting to occur between 172 and 290 °C. This is facilitated by weakening of the inter-layer interactions between alkyl chains, with longer, and/or branched chains resulting in lower melting temperatures.^18,19^

More recently, we have previously found meltable 3D hybrid organic–inorganic ABX_3_ structures. They are composed by both tributylmethylammonium and tetrapropylammonium as the A-site cation.^20^ The [TPrA][M(dca)_3_] (M = Mn, Fe, Co) series melt at temperatures ∼250 °C, *via* M–N coordination bond breaking and the formation of under-coordinated M centres. Interestingly, cooling of these high temperature liquids back to room temperature results in glass formation, like the recent example in two-dimensional perovskites.^21^ Furthermore, unlike existing glasses formed by melt-quenching MOFs,^22–25^ those formed from HOIPs exhibit interesting electronic/phononic properties with potential applications in *e.g.* thermoelectrics.^20^

**Fig. 1:**
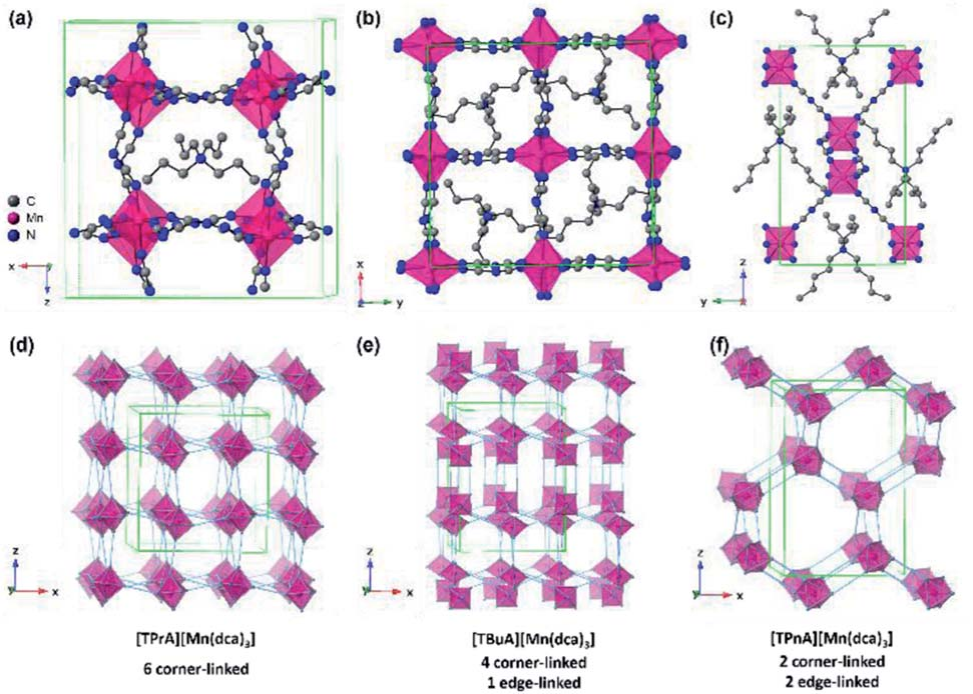
Simplified structure of (a) [TPrA][Mn(dca)_3_], (b) [TBuA][Mn(dca)_3_] and (c) [TPnA][Mn(dca)_3_] at 298 K respectively.^29^ All H atoms have been omitted for clarity. Only one orientation of the tetraalkylammonium and dca ions within the average crystal structure are shown for each ABX_3_ structure even though the average structures may have multiple orientations. Unit cells are indicated by green lines. (d)–(f) Indicate the polyhedral connectivity of each structure.

In this work, we look to lower melting temperatures through careful modification of A-site and B-site species. Specifically, we investigate the structure–property correlation between hybrid organic–inorganic ABX_3_ structures with different chemical compositions and their melting and glass transition temperatures (*T*_m_ and *T*_g_). We modify the size of the organic A cation in [TAlA][M(dca)_3_] (TAlA = tetralkylammonium linker, M = Mn, Fe, Co) structures through the introduction of successively larger tetrabutylammonium (TBuA = tetrabutylammonium, (CH_3_CH_2_CH_2_CH_2_)_4_N^+^) and tetrapentylammonium (TPnA = tetrapentylammonium, (CH_3_CH_2_CH_2_CH_2_CH_2_)_4_N^+^) species (Fig. 1). This results in a series of nine ABX_3_ structures, of which four are previously unreported. Structural characterisation techniques allow Tolerance Factors to be calculated for each, which are then linked to thermal analysis to try and rationalise the reduction in *T*_m_ upon increasing A cation size.^26^

## Results and discussion

### Synthesis and crystallography

The synthesis of all [TAlA][M(dca)_3_] materials was performed by solvent layering, and through equimolar replacement of precursor quantities in each case.^27^ Typically, 10 ml of an aqueous solution (2 mmol) of the metal salt was placed at the bottom of a thin crystallisation tube, and layered with a mixture of a sodium dicyanamide solution in 10 ml of water (6 mmol) and the tetraalkylammonium bromide in 10 ml of ethanol (2 mmol). Block-shaped single crystals were isolated from the mother liquor after one week of slow evaporation in an open atmosphere at 298 K. [TPrA][Mn(dca)_3_], crystallises in the previously reported tetragonal structure in the *P*4̄2_1_*c* space group at room temperature (Fig. 1a; *a* = 16.29 Å, *c* = 17.43 Å; Table 1).^20^ Here the dca ligand bridges metal centres through nitrogen atoms in a μ_1,5_ end-to-end fashion. This means that neighbouring metal-centered octahedra are corner-sharing, and create a pseudo-cubooctahedral cavity in which the tetraalkylammonium cation (TAlA) is located (Fig. 1d–f).

**Table 1:** Chemical and physical properties for the nine [TAlA][M(dca)_3_] compounds in this study

A	B	Space group	Structure type	*T*_d_ (°C)	*T*_m_ (°C)	Δ*H*_f_ (kJ mol^−1^)		Δ*S*_f_ (J mol^−1^ K^−1^)	Ref.
TPrA	Mn	*P*4̄2_1_*c*	Perovskite	281^a^	262^a^	47^a^	88^a^		15 and 27
TPrA	Fe	*Pnna*	Perovskite	271^a^	252^a^	50^a^		95^a^	12
TPrA	Co	*Pnna*	Perovskite	267^a^	212^a^	65^a^		134^a^	12
TBuA	Mn	*P*2_1_2_1_2	Triple rutile	282	185	59		128	27
TBuA	Fe	*P*2_1_2_1_2	Triple rutile	271	175	60		134	This work
TBuA	Co	*P*2_1_2_1_2	Triple rutile	271	143	63		152	This work
TPnA	Mn	*Pnna*	LiSbO_3_	283	149	56		132	27
TPnA	Fe	*Pnna*	LiSbO_3_	273	137	61		149	This work
TPnA	Co	*Pnna*	LiSbO_3_	272	106	59		155	This work

a The values were taken from our previous report.^20^

On the other hand, [TBuA][Mn(dca)_3_] crystallises in the orthorhombic space group *P*2_1_2_1_2, *a* = 16.01 Å, *b* = 16.01 Å, *c* = 21.55 Å (Fig. 1b). This is not a perovskite structure, but instead adopts a distorted triple rutile topology. The Mn coordination sphere contains three crystallographically independent μ_1,5_ dca anions. Each is joined to five Mn atoms. One is through a doubly bridged dca ligand, which creates an edge sharing MnN_6_ dimer unit. The further connection of each Mn centre to four additional atoms through single μ_1,5_ dca ligands then creates a 3D anionic framework. Hexagonal channels contain the TBuA cation.

[TPnA][Mn(dca)_3_] crystallises in the *Pnna* space group, *a* = 13.22 Å, *b* = 11.63 Å, *c* = 20.31 Å (Fig. 1c), and adopt a LiSbO_3_ structure. Here, each octahedral Mn coordination environment contains three crystallographically independent nitrile groups. These octahedra share edges with two neighbouring Mn coordination spheres through double μ_1,5_ dca ligands, which creates a zigzag chain motif. They also share edges with two additional Mn octahedra through single μ_1,5_ dca ligands, which link these chains together. The successive reduction of corner sharing linkages upon increase of the A site cation size facilitates the provision of space in which to accommodate the latter.

Fe and Co analogues of [TPrA][Mn(dca)_3_], possess the *Pnna* space group, with cell parameters: *a* = 11.52 Å, *b* = 11.54 Å, *c* = 17.44 Å and *a* = 17.22 Å, *b* = 23.13 Å, *c* = 22.85 Å for [TPrA][Fe(dca)_3_] and [TPrA][Co(dca)_3_], respectively, according to previous reported structures.^17^ On the other hand, Fe and Co analogues of [TBuA][Mn(dca)_3_] and [TPnA][Mn(dca)_3_] materials have not been previously reported.

In this work, single crystal X-ray diffraction studies (Fig. S1–S6 and Tables S1–S4†) confirmed the successful synthesis of these four structures ([TBuA][Fe(dca)_3_], [TBuA][Co(dca)_3_], [TPnA][Fe(dca)_3_] and [TPnA][Co(dca)_3_]). We confirmed that, similar to [TBuA][Mn(dca)_3_], both [TBuA][Fe(dca)_3_], *a* = 15.7971(7) Å, *b* = 15.8093(9) Å, *c* = 21.4138(11) Å and [TBuA][Co(dca)_3_], *a* = 15.7659(6) Å, *b* = 15.7712(5) Å, *c* = 21.2815(7) Å crystallise in the *P*2_1_2_1_2 space group.

Likewise, as [TPnA][Mn(dca)_3_], [TPnA][Fe(dca)_3_] (*a* = 13.1007(6) Å, *b* = 11.6128(5) Å, *c* = 20.0929(8) Å) and [TPnA][Co(dca)_3_] (*a* = 13.066(2) Å, *b* = 11.6026(19) Å, *c* = 19.926(4) Å) crystallise in the *Pnna* space group. In all cases, alkyl chains contain structural disorder, which is accentuated in the case of longer chains.

The cell parameters obtained were then used in a Pawley refinement of powder X-ray diffraction data collected on bulk samples (Fig. S7 and S8†), to confirm phase purity in each case. Altogether, these compounds, in addition with the previously reported [TPrA][M(dca)_3_] series (Table 1) provide an opportunity to relate chemical composition to changes in physical properties.

### Tolerance factors

The stability of perovskite structures has been extensively discussed, using values of the Tolerance Factor (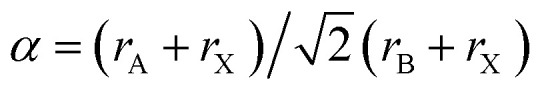, where *r*_A_, *r*_B_, *r*_X_ are the ionic radii of ‘A’ cation, ‘B’ metal and ‘X’ anion, respectively) proposed by Goldschmidt in 1926 for inorganic perovskites.^26^ Intense research has focused on using this relatively simple relation, which relies upon maximizing enthalpic interactions in the structure to predict whether a given chemical composition will form a cubic (0.8 < *α* < 1) or a distorted structure. Cheetham and co-workers have modified this relation, basing this on a rigid sphere model which treats highly anisotropic anions *e.g.* HCOO^−^, N_3_^−^, CN^−^, N(CN)_2_^−^ as rigid cylinders, with effective radius *r*_Xeff_ and an effective height *h*_Xeff_ for HOIPs (eqn (1)).^28^1
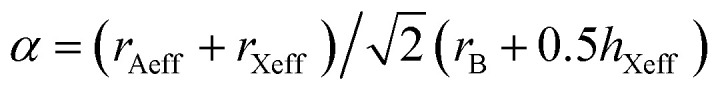
where, *r*_Aeff_ = *r*_mass_ + *r*_ion_, with *r*_mass_ being the distance between the centre of mass of the molecule and the atom with the largest distance to the centre of mass (excluding hydrogen atoms), and *r*_ion_ is the corresponding ionic radius of this atom.

Cheetham *et al.* found, using their adjusted relationship, that although cubic perovskites were still typically found in the region 0.9–1.0, values of 0.8–0.9 led to distorted perovskite structures, and those less than 0.8 generally led to other architectures such as ilmenite. On the other hand, values larger than 1.0 were observed to yield many hexagonal structures.

**Fig. 2:**
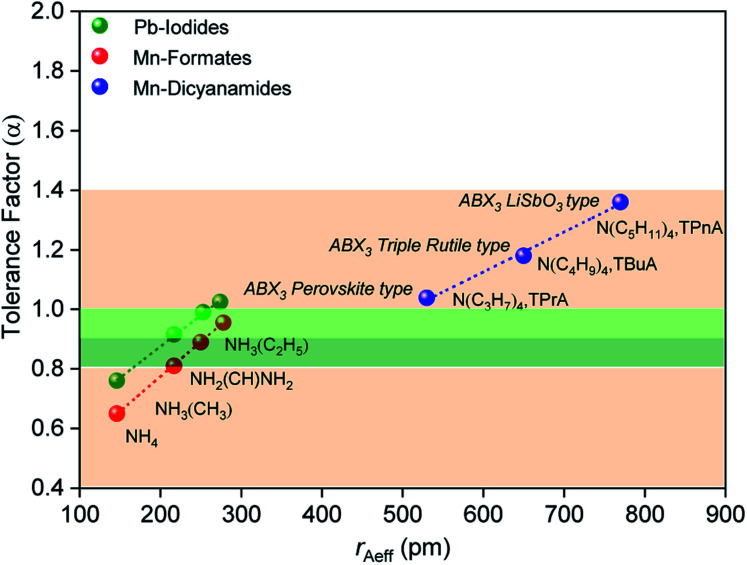
Values of effective radius of tetraalkylammonium cations is plotted with the calculated values of tolerance factor (*α*) for various ABX_3_ perovskite and polymorphic hybrid structures. The data for the current ABX_3_ perovskite and polymorphic Mn-dicyanamide structures (blue points) is shown here along with other ABX_3_ materials,^28^*e.g.* Mn-formate (red data point) and Pb-iodide (green data point). The light green and dark green shaded area (*α* = 0.8–1.0) highlights the range highlighted by Cheetham *et al.*, where perovskite and distorted perovskite structures are formed. Outside of this range (1.0 > *α* > 0.8, pink) indicates unstable and low symmetry structures.

The size of the A site cation in the dca-based materials studied here compared to those in known hybrid halides and formates, results in larger tolerance factors between 1.0 and 1.4 (Fig. 2), with these increasing upon increasing organic cation size (1.03 (±0.002) for [TPrA][Mn(dca)_3_], 1.18 (±0.003) for [TBuA][Mn(dca)_3_], 1.36 (±0.005) for [TPnA][Mn(dca)_3_]). The variation in ‘B’ metal from Mn → Fe → Co, also increases the tolerance values slightly (*e.g.* 1.03 (±0.002) for [TPrA][Mn(dca)_3_], 1.05 (±0.004) for [TPrA][Fe(dca)_3_], 1.07 (±0.004) for [TPrA][Co(dca)_3_], Fig. S9;† calculated using the values of constituent ions sizes in the structures presented by García *et al.*^12,15^).

### Thermal analysis

**Fig. 3:**
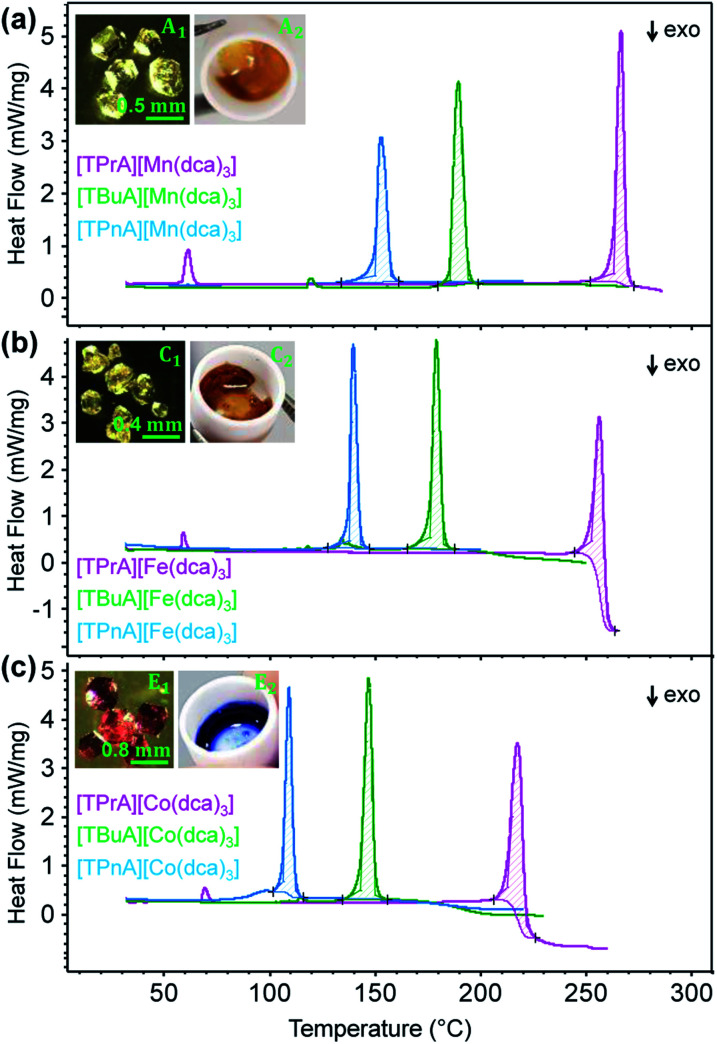
(a)–(c) The change in heat flow as a function of temperature for the [TBuA][M(dca)_3_] and [TPnA][M(dca)_3_] samples synthesized in this study, alongside previously reported data for [TPrA][M(dca)_3_].^20^ Inset of (a), (b) and (c) shows the optical images of crystalline solid and molten liquid (taken instantly after opening the heating furnace at high-*T* near *T*_m-offset_) for [TBuA][Mn(dca)_3_], [TBuA][Fe(dca)_3_] and [TBuA][Co(dca)_3_] respectively. Optical images for all other materials (B_1_ − F_1_, B_2_ − F_2_) are given in Fig. S11.†

Thermogravimetric analysis (TGA) and differential scanning calorimetry (DSC) experiments were carried out on the [TBuA][M(dca)_3_] and [TPnA][M(dca)_3_] samples. On subsequent data analysis, values for *T*_m_, the enthalpy of fusion (Δ*H*_f_), the entropy of fusion (Δ*S*_f_) and the temperature of decomposition (*T*_d_), were identified (Fig. 3, Table 1). Those for the [TPrA][Co(dca)_3_] and [TPrA][Fe(dca)_3_] may be overestimated due to the change in baseline at high temperature (Table S5†). A decreasing trend in *T*_m_ values with the increasing number of carbon atoms in A site chains is evident, *i.e.* [TPnA][M(dca)_3_] < [TBuA][M(dca)_3_] < [TPrA][M(dca)_3_]. This would be consistent with the different structure types afforded upon increasing A site cation size allowing the dca ligands to achieve a greater degree of thermal libration.

Though linear trends across in the set of the nine compounds were not observed, the decrease in *T*_m_ is, in general, consistent with larger values of Δ*S*_f_. The increment of the Δ*S*_f_ values is related with the presence of larger alkyl chains in the alkylammonium cations in the solid state. This is accordance with the discussion adopted by Mason *et al.* in their network-forming bis(acetamides).^29,30^

**Fig. 4:**
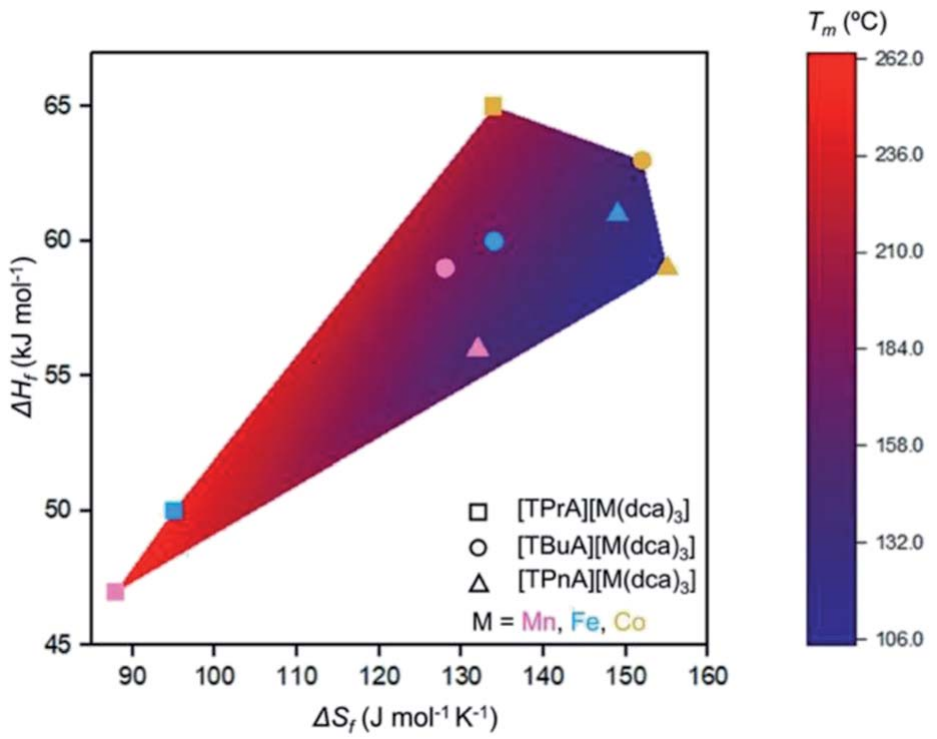
Comparison of Δ*H*_f_, Δ*S*_f_, and *T*_m_ for the series [TAlA][M(dca)_3_]. The symbol identifies the tetraalkylammonium cation and the symbol colour designates the metal.

Any trends in Δ*H*_f_ and alkyl chain length are less clear. This is because the melting does not imply the bond breaking within the alkylammonium cation itself, but rather the M–N coordination bonds in the surrounding framework (Fig. 4). This suggests that melting in these materials is dominated by entropic effects.

Trends upon changing the B-site transition metal ion are also present within each tetraalkylammonium set (TPrA, TBuA and TPnA). The trend in *T*_m_ is the same in each case, *i.e.* Co < Fe < Mn. These reductions were however, subtler than the effect of changing the A site cation, resulting in for example a lowering in *T*_m_ from 149 °C for [TPnA][Mn(dca)_3_], to 137 °C for [TPnA][Fe(dca)_3_] and then to 106 °C for [TPnA][Co(dca)_3_] (Table 1).

This follows the trend in ionic radius, which decreases in the order Mn > Fe > Co and consistent with hard-soft/acid-base theory as suggested previously.^20^

### Structural changes upon melting

To provide an atomic level insight into the changes in bonding upon heating into the liquid state, X-ray total scattering experiments were performed on the crystalline ABX_3_ structures. Variable temperature total scattering measurements were carried out on [TBuA][Mn(dca)_3_] and [TPnA][Mn(dca)_3_] from 34 °C to 258 °C (*i.e.* below *T*_d_ in each case) to determine changes in local structure.

**Fig. 5:**
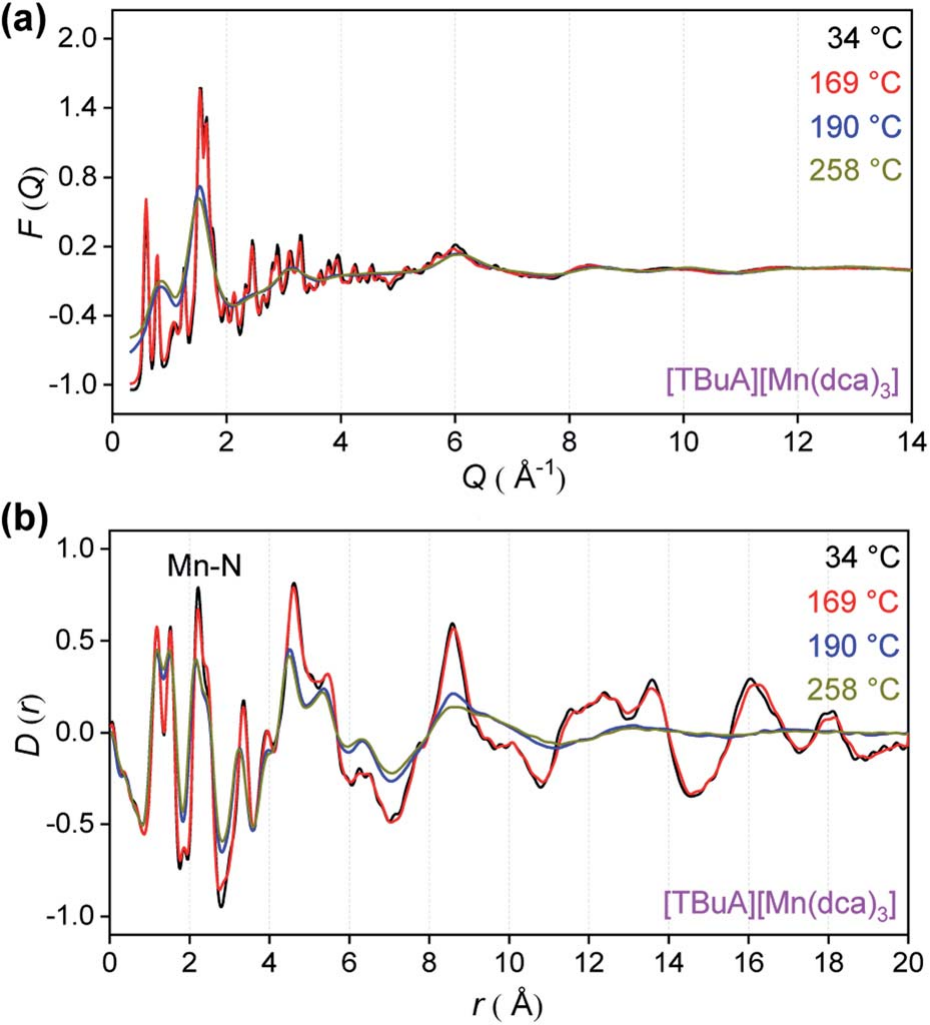
(a) X-ray structure factors, *F*(*Q*) and (b) pair distribution functions (PDF), *D*(*r*) for [TBuA][Mn(dca)_3_] upon heating. Only selected temperatures are shown for clarity, see Fig. S12† for full data.

The structure factors, *F*(*Q*), of [TBuA][Mn(dca)_3_] cease to contain sharp features above 190 °C, indicating the loss of long-range crystalline order at this temperature (Fig. 5a, complete dataset shown in Fig. S12a†). This temperature coincides with the *T*_m_ observed in DSC experiments (*T*_m-onset_ = 185 °C). No further significant changes in the *F*(*Q*)s occurred until the maximum temperature of 258 °C reached in the experiment. A similar experiment for [TPnA][Mn(dca)_3_] demonstrated a loss of sharp features from the *F*(*Q*) at 169 °C, which again agrees with the DSC experiments (*T*_m-onset_ = 149 °C) (Fig. S13a†).

The *F*(*Q*)*s* for both samples collected after cooling back to room temperature from the last experimental temperature (258 °C) provided evidence of recrystallisation back to the original structure in each case (Fig. S12a and S13a†).

Pair distribution functions (PDF), *D*(*r*)s, were extracted from the *F*(*Q*)*s* after appropriate data corrections using experimental pycnometric densities (Table S6†).^31^ The variable temperature PDFs of [TBuA][Mn(dca)_3_] (Fig. 5b, full dataset in Fig. S11b†) demonstrate a reduction in the strength of the peaks and troughs above 10 Å at 190 °C, consistent with liquid-like atom–atom correlations above the framework *T*_m_. No further substantial changes were observed, up to the maximum temperature of the experiment at 258 °C. A similar scenario is observed in the PDFs of [TPnA][Mn(dca)_3_] (Fig. S12b†), with the disappearance of strong peaks in the correlations above 10 Å at temperatures above the *T*_m_ observed in DSC measurements.

To evaluate the specific atom–atom pair correlations to the PDF peaks, the published structure for the [TBuA][Mn(dca)_3_] crystalline sample was refined against the PDF data (Methods, Fig. S13†).^27,32^ The weighted partial PDFs, *g*(*r*), were then calculated from the refined structure, and show that the Mn–N correlation at *ca.* 2.22 Å is accompanied by small contributions from nearby C–C and C–N correlations at 2.45 Å and 2.57 Å respectively (Fig. S14†).

The position, full-width at half-maximum (FWHM) and area of the C–C, C–N and Mn–N correlations were fitted using a Gaussian function. The proximity of the C–C (2.45 Å) and C–N (2.57 Å) correlations to that for Mn–N (2.22 Å), rendered it necessary to fit the experimental peak using multiple peak fitting. Regression analysis is shown in Fig. S16† and the extracted parameters are tabulated in Table S7.† The substantial contribution of instrumental broadening to the peaks (2π/*Q*_max_ = 0.29 Å) dominates the measurements, and, combined with correlations in the fitting parameters from overlapping peaks, direct links to the Lindemann criterion for melting could not be made.^33^

Nevertheless, the temperature variation of the FWHM, peak positions and areas at *T*_m_ clearly show marked changes, which may be consistent with a melting process involving Mn–N bond breakage and some thermal decomposition of both organic species (Fig. S17†). A similar analysis for [TPnA][Mn(dca)_3_] (Fig. S18–S21†) was carried out and is also suggestive of changes at *T*_m_.

### Glass formation

**Fig. 6:**
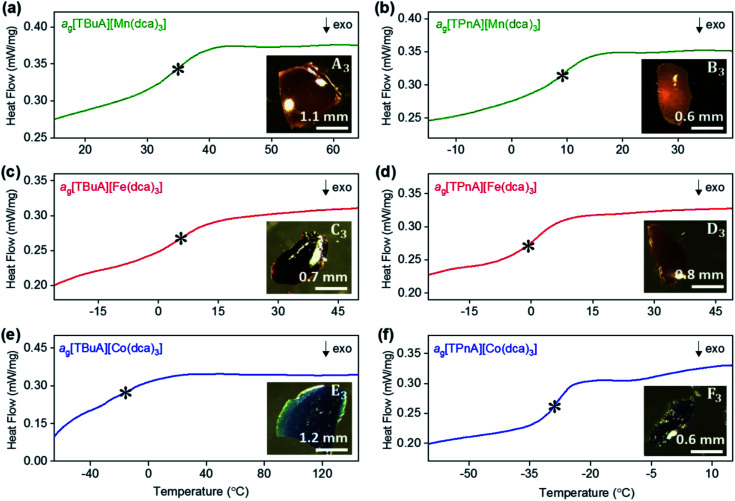
Change in heat flow as a function of temperature for (a) a_g_[TBuA][Mn(dca)_3_], (b) a_g_[TPnA][Mn(dca)_3_], (c) a_g_[TBuA][Fe(dca)_3_], (d) a_g_[TPnA][Fe(dca)_3_], (e) a_g_[TBuA][Co(dca)_3_], (f) a_g_[TPnA][Co(dca)_3_]. Onset of glass transitions (*T*_g_) were evaluated and marked with asterisk. Full temperature scans were shown in ESI.† Inset shows the optical images of each glasses. All experiments performed in an argon atmosphere.

As in our previous report on *a*_g_[TPrA][M(dca)_3_] samples,^20^ opaque, glass-like pieces were observed after cooling the melts from their offset of melting near *T*_d_ (insets of Fig. 6a–e), and found to be amorphous by X-ray diffraction (Fig. S22 and S23†). The glasses, in keeping with existing nomenclature on hybrid glasses, are thus termed *a*_g_[TAlA][M(dca)_3_] (*a*_g_: melt-quenched amorphous).

The glass transition temperatures (*T*_g_) of all *a*_g_[TBuA][M(dca)_3_] and *a*_g_[TPnA][M(dca)_3_] (M = Mn, Fe, Co) melt-quenched glasses were obtained in DSC experiments each case (Fig. 6). Complete cycles of heating–cooling–heating runs are shown in the ESI (Fig. S24–S29†). The low values of *T*_g_ (33 °C to −26 °C), are broadly consistent with both their low *T*_m_ values (185 °C to 106 °C), and the empirical “*T*_g_/*T*_m_ ∼ 2/3” law (Table 2).^34^ Their location below room temperature is similar to the behaviour of dca based ILs, which generally exhibit glass transitions below room temperature (*e.g.* −67 °C for [N_8444_][dca]).^35,36^

**Table 2:** Properties of glass transitions in melt-quenched *a*_g_[TAlA][M(dca)_3_]

Samples	*T*_g_ (°C)	*T*_g_/*T*_m_^b^	Ref.
*a*_g_[TPrA][Mn(dca)_3_]^a^	223	0.89	20
*a*_g_[TPrA][Fe(dca)_3_]^a^	225	0.90	20
*a*_g_[TPrA][Co(dca)_3_]^a^	125	0.82	20
*a*_g_[TBuA][Mn(dca)_3_]	33	0.66	This work
*a*_g_[TBuA][Fe(dca)_3_]	6	0.62	This work
*a*_g_[TBuA][Co(dca)_3_]	−12	0.63	This work
*a*_g_[TPnA][Mn(dca)_3_]	9	0.66	This work
*a*_g_[TPnA][Fe(dca)_3_]	−1	0.66	This work
*a*_g_[TPnA][Co(dca)_3_]	−26	0.65	This work

a The values were taken from our previous report.^20^

b Ratio of *T*_g_/*T*_m_ is calculated from temperatures in kelvin.

These results are consistent with the observation that *a*_g_[TPnA][Fe(dca)_3_] and *a*_g_[TPnA][Co(dca)_3_] remain viscous after quenching at room temperature and take a relatively long time to solidify (see Methods). This same phenomenon also explains why these melts in particular appear prone to partial recrystallisation after cooling from offset of *T*_m_ (Fig. S30†).

Heating these structures near to *T*_d_ and the use of slower cooling rates prevented recrystallization in *a*_g_[TBuA][M(dca)_3_] and *a*_g_[TPnA][M(dca)_3_].^20^ Minimal gravimetric mass loss (∼2.0%) was detected upon quenching the melts at *ca.* 10 °C min^−1^ near to room temperature (Fig. S31 and S32†). However, a slightly higher mass loss (∼5.0%) was detected during quenching at slow cooling rates (Fig. S33†), which would be consistent with some degree of ligand decomposition. This is reflected in the FT-IR spectra of the glasses which combines of two (weak) new bands at 1629–1634 cm^−1^ and 802–806 cm^−1^ as reported previously (Fig. S34†).^20,37–39^

The behaviour is very similar to dca-based organic–inorganic salts, which possess ionic liquid (IL) character at room temperature.^35,36^ MacFarlane *et al.* showed that, for alkylpyrrolidinium-dca based ILs, the increase in alkyl chain length in pyrrolidinium organic cations *e.g.* from [P_13_][dca] to [P_16_][dca] (where P_13_ = *N*-propyl-*N*-methylpyrrolidinium, P_16_ = *N*-hexyl-*N*-methylpyrrolidinium) increases the entropy of melting (Δ*S*_f_ = 5 J mol^−1^ K^−1^ for [P_13_][dca], 95 J mol^−1^ K^−1^ for [P_16_][dca]).^36^ They also showed a decrease in viscosity from 50 to 45 cP (at 20 °C) with increase in cation chain length from [P_14_][dca] to [P_16_][dca] (where P_14_ = *N*-butyl-*N*-methylpyrrolidinium). The ABX_3_ structures studied here behave in the same way, in which the increase in alkyl chain length (steric effect) in A-site organic cation from *a*_g_[TPrA][M(dca)_3_] to *a*_g_[TPnA][M(dca)_3_] is offset by the larger entropy of melting (*e.g.* Δ*S*_f_ = 95 J mol^−1^ K^−1^ for *a*_g_[TPrA][Fe(dca)_3_], 149 J mol^−1^ K^−1^ for *a*_g_[TPnA][Fe(dca)_3_]) (Fig. 4), and hence accounts for the less viscous character in TPnA containing ABX_3_ structures at room temperature.

### X-ray structural characterisation – PDF analysis

**Fig. 7:**
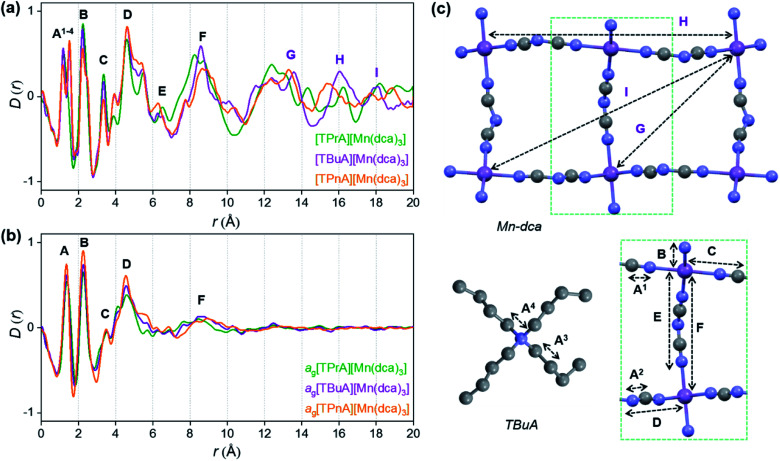
Pair distribution functions for [TPrA][Mn(dca)_3_],^20^ [TBuA][Mn(dca)_3_] and [TPnA][Mn(dca)_3_] (a) crystals and (b) glasses at room temperature, with the atom pairs that contribute most of the intensity in the labelled peaks indicated inside the (c) dotted structural fragment (A–F) of [TBuA][Mn(dca)_3_] for PDF peak assignment using the published CIF at 298 K (ref. 27) (violet: Mn; grey: C; blue: N; H atoms omitted for clarity).

*Ex situ*, room temperature PDF data were also collected on the three [TAlA][Mn(dca)_3_] (TAlA = TPrA,^20^ TBuA, TPnA) samples (Fig. 7a), and the calculated partial pair distributions functions were used to aid feature assignment (Figs. S14–S19†). Short range correlations (up to 10 Å) are ascribed to those between atoms present in a single Mn–dca–Mn linkage (*i.e.* green bounded portion of Fig. 7c) and those between atoms within A-site molecules, which are largely invariant in position upon increasing the size of the A cation. Specifically, the correlation noted at *r* = ∼1.1 to 1.3 Å, contains contributions from C–C, C–N (including C≡N) atom pairs (
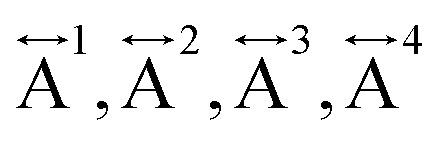
). The correlation at *r* = 2.25 Å is ascribed to the Mn–N pair (
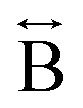
) and the two peaks at 3.3 Å and 4.7 Å are ascribed to the Mn–N–C (
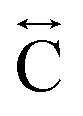
) and Mn–N–C–N (
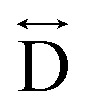
) correlations respectively. The major contributions to the peak at *r* = ∼8.2 Å (
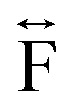
) arise primarily from the Mn–Mn correlation.

Correlations at high-*r* (>10 Å) are dominated by those between atoms in adjacent Mn–dca pairs (
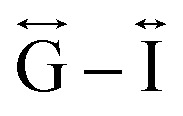
), and as such are more heavily influenced by the increase in A cation size. As expected, changes in the B site metal appeared to have negligible effect on the observed correlations (Fig. S35†).

The local structures of vitrified *a*_g_[TBuA][Mn(dca)_3_] and *a*_g_[TPnA][Mn(dca)_3_] were probed by using samples prepared in the DSC experiments (see Methods). The resultant *F*(*Q*)s did not contain any Bragg scattering (Fig. S36a and S37a†), confirming their amorphous nature (Fig. S36a–c†).

We found similar results for *a*_g_[TBuA][Fe(dca)_3_] and *a*_g_[TBuA][Co(dca)_3_] materials (Fig. S36†). The short-range correlations (
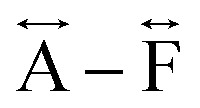
) present in the glass samples were relatively similar to the crystalline precursors (Fig. 7b and S36e–f†). Correlations belonging to Mn–N–C (
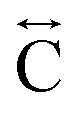
) and Mn–N–C–N (
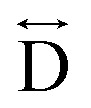
) were however reduced in intensity and substantially broadened. Prominent correlations were also not observed beyond 10 Å, consistent with the mechanism of melting involving the breakage of M–N bonds, and the associated movement of the A-site cation out of the central cavity.^20^

Difficulties in preparing a sufficient quantity of finely ground solid samples for PDF measurements of *a*_g_[TPnA][Fe(dca)_3_] and *a*_g_[TPnA][Co(dca)_3_] (owing to their viscous melts), led us to obtain variable temperature PDF data for these two compounds. Data collected in the liquid state for each bore a strong resemblance to those for other glass samples in this study, maintaining the same correlations as preheated material and with a similar behavior to their homologous *a*_g_[TPnA][Mn(dca)_3_] (Fig. S37e–f†).

### Magnetism study

Temperature dependent DC magnetic susceptibility measurements were carried out for all crystalline and melt-quenched glass samples, and the variation of effective magnetic moment, *μ*_eff_ (calculated from *χ*_M_: molar magnetic susceptibility, *μ* (B.M.) = 2.83√*χ*_M_T) is plotted as a function of *T* in Fig. S38–S40.†^40^ Room temperature magnetic moments (*μ*_RT_) for all crystalline phases are as per the number of unpaired spins present in their high spin M^2+^ ‘d’ orbital configurations. The decrease at low temperatures in all cases demonstrates a contribution of zero-field splitting and a weak antiferromagnetic coupling.^25^ Interestingly, the *μ*_RT_ for the glasses were found to be slightly lower than those for the corresponding crystalline phases, indicating a reduction in oxidation state for a proportion of metal centres during melt-quenching (M^2+^ → M^0^). These differences were used to calculate the percentage of metal ions reduced in each case (*a*_g_[TBuA][Mn(dca)_3_] – 1.7%, *a*_g_[TPnA][Mn(dca)_3_] – 0.5%, *a*_g_[TBuA][Fe(dca)_3_] – 3.9%, *a*_g_[TPnA][Fe(dca)_3_] – 4.5%, *a*_g_[TBuA][Co(dca)_3_] – 2.0%, *a*_g_[TPnA][Co(dca)_3_] – 2.8%).These are lower than the reduction observed in the *a*_g_[TPrA][Mn(dca)_3_] series, which is consistent with their lower *T*_m_s and wider temperature range between *T*_m_ and *T*_d_. Elemental compositions of both crystalline and glassy states were also similar (Table S8†).

## Conclusions

The results reported in this work demonstrate the effect of chemical composition and structure type on the melting temperature of ABX_3_ hybrid organic–inorganic materials. Specifically, the comparison of four novel [TAlA][M(dca)_3_] structures with 5 known materials facilitated a study of the relationship of thermal properties to chemical structure. The size of A-site alkylammonium cation and B-site transition metal was altered and found to influence *T*_m_. In particular, increasing the size of the A-site cation from TPrA → TBuA → TPnA was observed to result in a decrease in *T*_m_ through an increase in Δ*S*_f_.

The majority of the melts formed were found to recrystallise partially upon cooling from temperatures (near their *T*_m-offset_). To form the glasses, annealing near their *T*_d_ was necessary, which suggests a small degree of decomposition of the organic linker occurs. Those structures possessing bulkier TPnA A-site cations remained viscous after quenching at room temperature, and solidified only after several days, suggesting a further structural change. The viscous character of *a*_g_[TPnA][Fe(dca)_3_] and *a*_g_[TPnA][Co(dca)_3_] is linked to their high Δ*S*_f_ values upon melting and below-room temperature *T*_g_s. Alongside relatively low values of *T*_g_ below room temperature indicates the kinematic similarity of this dca based ABX_3_ melts with dca based ionic liquids. Such basic observations may provide further rules for the design of glass forming hybrid structures.

Previously, we have shown that the glasses formed *via* melt-quenching HOIPs possess potential as thermoelectric materials, owing to their moderate electrical conductivity as well as very low thermal conductivity values. This study provides a rational for altering the physical properties of the starting perovskite and non-perovskite type ABX_3_ materials, and at the same time opens up directions in forming further examples of functional liquid and glasses from ABX_3_ hybrid structures.

## Experimental methods

### Thermal analysis

Simultaneous thermogravimetric and calorimetric analysis (TGA/Heat flow) were carried out in a SDT apparatus (TA Q600). Data were collected in the range from 25 °C to 400 °C at a scan rate of 10 °C min^−1^ under an argon atmosphere.

To obtain the liquid states, samples (∼10 mg) were placed into a 70 μl alumina crucible and heated above their respective melting offsets at a heating rate of 10 °C min^−1^ in SDT TA Q600 under argon atmosphere. Differential Scanning Calorimetry (DSC) measurements were conducted using a TA Q2000 instrument. To obtain the glass transitions, the samples were reheated after cooling to low temperatures.

### Preparation of glasses

#### *a*_g_[TBuA][Mn(dca)_3_]

The [TBuA][Mn(dca)_3_] crystal was heated at 10 °C min^−1^ to 280 °C, then cooled under an argon atmosphere (flow rate 50 ml min^−1^) to −20 °C at *ca.* 3 °C min^−1^.

#### *a*_g_[TPnA][Mn(dca)_3_]

The [TPnA][Mn(dca)_3_] crystal was heated at 10 °C min^−1^ to 282 °C, then cooled under an argon atmosphere (flow rate 50 ml min^−1^) to −50 °C at *ca.* 3 °C min^−1^.

#### *a*_g_[TBuA][Fe(dca)_3_]

The [TBuA][Fe(dca)_3_] crystal was heated at 10 °C min^−1^ to 265 °C, then cooled under an argon atmosphere (flow rate 50 ml min^−1^) to −50 °C at *ca.* 5 °C min^−1^.

#### *a*_g_[TPnA][Fe(dca)_3_]

The [TPnA][Fe(dca)_3_] crystal was heated at 10 °C min^−1^ to 265 °C, then cooled under an argon atmosphere (flow rate 50 ml min^−1^) to −70 °C at *ca.* 5 °C min^−1^. The highly viscous melt formed after was kept for 72 hours at room temperature. Note: solidification of the viscous sample at room temperature might result in partial recrystallisation and so successive heating may require to remove the remnant crystallisation and achieve X-ray amorphous samples, Fig. S23b.†

#### *a*_g_[TBuA][Co(dca)_3_]

The [TBuA][Co(dca)_3_] crystal was heated at 10 °C min^−1^ to 255 °C, then cooled under an argon atmosphere (flow rate 50 ml min^−1^) to −70 °C at *ca.* 5 °C min^−1^.

#### *a*_g_[TPnA][Co(dca)_3_]

The [TPnA][Co(dca)_3_] crystal was heated at 10 °C min^−1^ to 260 °C, then cooled under an argon atmosphere (flow rate 50 ml min^−1^) to −80 °C at *ca.* 5 °C min^−1^. The highly viscous melt formed after was kept for 72 hours at room temperature. Note: solidification of the viscous sample at room temperature might result in partial recrystallisation and so successive heating may require to remove the remnant crystallisation and achieve X-ray amorphous samples, Fig. S23c.†

### Powder X-ray diffraction

Ambient temperature: X-ray powder diffraction (PXRD) patterns were recorded (2*θ* = 5°–60°) on a Bruker D8 Advance diffractometer (equipped with a LynxEye EX linear position sensitive detector) in Bragg–Brentano geometry using Cu Kα (*λ* = 1.540598 Å) source fitted with a Ni 0.012 mm filter. Data were collected in 2*θ* step size of 0.02°, with 10 s per step.

### X-ray total scattering experiments

Variable temperature measurements were performed on a sample of crystalline [TBuA][Mn(dca)_3_], [TPnA][Mn(dca)_3_], [TPnA][Fe(dca)_3_] and [TPnA][Co(dca)_3_]. Data were collected at the I15-1 beamline at the Diamond Light Source, UK (*λ* = 0.158345 Å, 78.3 keV) in the range 0.6 < *Q* < 24 Å^−1^. A finely ground sample of the crystal was loaded into a 1 mm-diameter silica capillary under argon atmosphere using a glove box, and capped with glue along with glass wool to hold it in place during the melting process. Data were collected at a temperature of 34 °C, and then upon subsequent heating at 114, 145, 169, 190, 211, 236, 258 °C and then back to 30 °C. Room temperature measurements were performed for [TBuA][Mn(dca)_3_], [TBuA][Fe(dca)_3_], [TBuA][Co(dca)_3_] and [TPnA][Mn(dca)_3_] crystalline and melt-quenched glass samples in an identical manner to those used for the high temperature measurements. Data on the sample, empty instrument and capillary were collected in the region of 0.6 < *Q* < 24 Å^−1^. Background, multiple scattering, container scattering, Compton scattering, and absorption corrections were performed using the GudrunX program.^31^

### Refinement of structures against pair distribution function data and calculation of partial pair distribution functions

Published structural models were refined against PDF data using PDFGui.^32^ Starting values used were: *Q*_damp_ = 0.08, *S*_ratio_ = 1, and model scale factor = 1.0. Values set and not refined were *r*_cut_ = 5.75 Å, data scale factor = 0.5, and *Q*_broad_ = 0.0001. Isotropic thermal parameters were used for all atoms, initially set to the same value of 0.003 Å^2^. Refinements were done in the range 0.5 < *r* < 20 Å, with *Q*_max_ = 22 Å^−1^.

The published structure of [TBuA][Mn(dca)_3_] included positional disorder in the dca anion and in the A site molecule, modelled by partial occupancies of multiple sites. To enable refinement, only one of each multiple site options was chosen to give chemically sensible linkers and cations. The chosen sites were assigned full occupancy and all other sites were discarded. The final structural model was consistent with the given chemical formula. The atomic positions of the dca linkers were refined with appropriate symmetry constraints, and no positions appeared significantly different from their starting ones. The atomic positions of the A site cations were not refined, given the substantial disorder of this site. Four distinct thermal parameters for Mn, C, H and N were refined isotropically. Note that a good fit using this model is not expected; the disorder in the published structure strongly implies that the positions of the molecular ions will vary from one-unit cell to another. It is not possible for PDFGui to accurately account for these differences using a ‘small box’ model based on a single unit cell and this is reflected in the relatively poor fits.

An identical treatment was also applied to the published structure of [TPnA][Mn(dca)_3_].

### Magnetism study

A SQUID MPMS 3 instrument was used to conduct the magnetic measurements of crystals and glasses. For *a*_g_[TPnA][Fe(dca)_3_] and *a*_g_[TPnA][Co(dca)_3_], we have used semi-solid glass samples. The temperature variation of field-cooled susceptibility (*M*–*T*) data was collected at 500 Oe magnetic field at a temperature range 2–300 K. Samples were placed in a light weight homogeneous quartz tube to minimize the background noise and stray field effects. The magnetic data were corrected for the diamagnetic contribution from the quartz sample holder and the intrinsic diamagnetism of the samples by the standard literature using Pascal's constants.^41^

### CHN elemental analysis

The elemental compositions were obtained from CHN analyzer. Ground powder samples (highly viscous liquid samples for *a*_g_[TPnA][Fe(dca)_3_] and *a*_g_[TPnA][Co(dca)_3_]) were used to perform the measurements using CE440 Elemental Analyzer, EAI Exeter Analytical Inc.

## Data availability

All data available in ESI.†

## Author contributions

B. K. S. and T. D. B. designed the project. C. C. B. and M. L. R. G. collected and analysed the single crystal X-ray diffraction data. T. D. B., M. F. T., L. N. M., D. K., T. F., P. A. C. and M. D. L. collected the X-ray total scattering data. B. K. S., D. A. K. and T. D. B. analysed the data. B. K. S. performed and analysed all other experimental data. B. K. S., C. C. B. and T. D. B. wrote the manuscript with input from all authors.

## Conflicts of interest

There are no conflicts to declare.

## Supplementary Material

SC-013-D1SC07080K-s001

SC-013-D1SC07080K-s002
