# Supplementary material for: Principles of melting in hybrid organic–inorganic perovskite and polymorphic ABX3 structures
Source: Chem Sci. 2022 Jan 20;13(7):2033–42. doi: 10.1039/d1sc07080k (PMC8849004; doi:10.1039/d1sc07080k)
Supplement: SC-013-D1SC07080K-s001 [file SC-013-D1SC07080K-s001.pdf]

Electronic Supplementary Material (ESI) for Chemical Science.
This journal is © The Royal Society of Chemistry 2022

Supporting Information

Principles of Melting in Hybrid Organic-Inorganic Perovskite and Polymorphic ABX_3 Structures

Bikash Kumar Shaw,[a] Celia Castillo-Blas,[a,b] Michael F. Thorne,[a] María Laura Ríos Gómez,[a] Tom Forrest,[c] Maria Diaz Lopez,[c] Philip A. Chater,[c] Lauren N. McHugh,[a] David A. Keen,[d] and Thomas D. Bennett*[a]

[a] Department of Materials Science and Metallurgy, University of Cambridge, CB3 0FS, UK.

[b] Departamento de Química Inorgánica, Universidad Autónoma de Madrid, 28049 Madrid, Spain.

[c] Diamond Light Source Ltd, Diamond House, Harwell Campus, Didcot, Oxfordshire, OX11 0DE, UK.

[d] ISIS Facility, Rutherford Appleton Laboratory, Harwell Campus, Didcot, Oxfordshire, OX11 0QX, UK.

Synthesis

Reagents:

$Mn(NO_3)_2\cdot4H_2O$ (98.5%, Sigma-Aldrich), $FeCl_2\cdot4H_2O$ (98%, Sigma-Aldrich), $Co(NO_3)_2\cdot6H_2O$ (98%, Sigma-Aldrich), (TBuA)Br (98%, Aldrich), (TPnA)Br (98%, Aldrich) and Na(dca) (96%, Sigma-Aldrich) were purchased as indicated and used as received.

Procedure:

The synthesis reported in the literature was followed.[1] Specifically, 10 ml of an aqueous solution of metal salt (2 mmol M: Mn^{2+}, Fe^{2+}, Co^{2+}) was placed at the bottom of a thin crystallisation tube and layered with a mixture of a solution of Na(dca) (6 mmol in 10 ml of water) and (TPrA)Br, (TBuA)Br or (TPnA)Br (2 mmol in 10 ml of ethanol). Block-shaped single crystals were obtained from the mother liquor after one week of slow evaporation in an open atmosphere.

Experimental and Refinement Details: [TBuA][Fe(dca)$_3$] (Orthorhombic)

(CCDC Deposition Number 2026498)

The experiment was performed on a colourless crystal of [TBuA][Fe(dca)$_3$] (0.10× 0.06 × 0.06 mm^3), with a prism shape. The crystal was placed in a 0.1 mm diameter polyamide loop and mounted on a Bruker four circle kappa-diffractometer equipped with a Cu INCOATED microsource, operated at 30 W power (45 kV, 0.60 mA) to generate Cu Kα radiation (λ = 1.54178 Å), and a Bruker VANTEC 500 area detector (microgap technology) at 293(2) K. Diffraction data were collected over reciprocal space in a combination of φ and ω scans to reach a resolution of 0.85 Å, with a completeness > 95%, and redundancy >3. For this, either a generic hemisphere collection strategy or a specific one determined using Bruker APEX3 software suite was used. The exposure time was adjusted based on the size and diffracting quality of the specimen, each exposure covering 1º in ω or φ. Unit cell dimensions was determined for least-squares fit of reflections with I>4σ. The structure was solved by direct methods implemented in SHELX package in the orthorhombic $P2_12_12$ space group with $Z = 8$. This crystal structure is complicated by disorder, both in the dca ligands and in the TBuA cations.[1] In addition, this structure was solved on the basis of diffraction data from pseudomerohedrally twinned crystals applying the twin law: (-1.0,0.0, 0.0; -1.0, 0.0, 0.0; 0.0, 0.0, -1.0). The hydrogen atoms were fixed at their calculated positions using distances and angle constraints. All calculations were performed using APEX3 software for data collection and OLEX2-1.2[2] and SHELXTL[3] to resolve and refine the structure. Modelling of electron density led to the identification of an asymmetric unit (atom in crystallographic independent positions) with one $(N_2C_3)_2$ structural unit at the site A and one $Fe_4N_{18}C_{12}$ structural unit at the site B. There was no significant residual density indicating the presence of solvent molecules in the structure. The structural unit in site A is disordered between two slightly different configurations with complementary occupancies. All non-hydrogen atoms in the structure were refined anisotropically. Final refinement was executed in 80 cycles and the final flux matrix F^2 converged to $R_1 = 0.1067$ ($F_o > 4\sigma F_o$) and $wR_2 = 0.3345$ (all data), with GooF = 1.048.

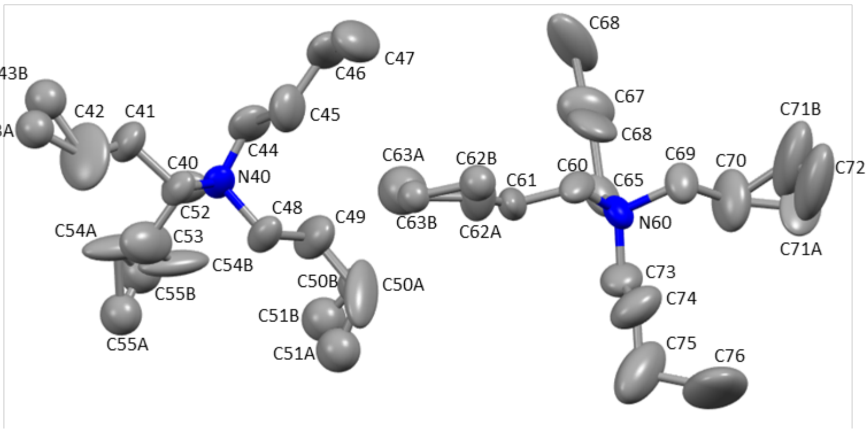

Figure S1. Graphical representation using thermal ellipsoids of the $[TBuA]^+$ cation associated with N40 and N60 in compound $[TBuA][Fe(dca)_3]$, integrated by two NC_{16} structural unit at the site A. Carbon atoms exhibit positional disorder. Hydrogen atoms are omitted for clarity.

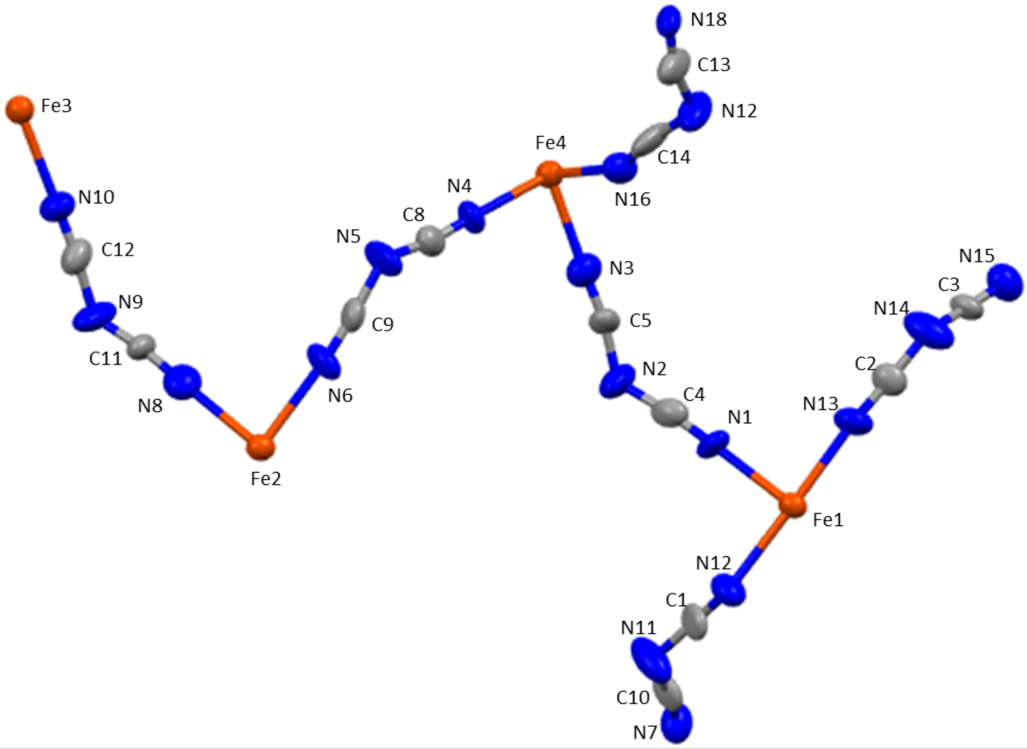

Figure S2. Graphical representation using thermal ellipsoids of the $Fe(dca)_3^-$ anion in compound $[TBuA][Fe(dca)_3]$, integrated by $Fe_4N_{18}C_{12}$ structural unit at the site A. Carbon atoms exhibit positional disorder. Hydrogen atoms are omitted for clarity.

Table S1. Crystal data and structure refinement details for [TBuA][Fe(dca)$_3$].

Empirical formula	$C_{22}H_{36}FeN_{10}$
Formula weight	496.39
Temperature/K	240
Crystal system	orthorhombic
Space group	$P2_12_12$
a/Å	15.7971(7)
b/Å	15.8093(9)
c/Å	21.4138(11)
α/°	90
β/°	90
γ/°	90
Volume/Å^3	5347.9(5)
Z	8
ρ_{calc}g/cm^3	1.233
μ/mm^{-1}	4.747
F(000)	2111.0
Crystal size/mm^3	0.2 × 0.2 × 0.2
Radiation	CuKα (λ = 1.54178)
2Θ range for data collection/°	4.126 to 130.676
Index ranges	$-18 \leq h \leq 18$, $-17 \leq k \leq 18$, $-25 \leq l \leq 25$
Reflections collected	55212
Independent reflections	9065 [R_{int} = 0.1126, R_{sigma} = 0.0817]
Data/restraints/parameters	9065/18/626
Goodness-of-fit on F^2	1.048
Final R indexes [I>=2σ (I)]	R_1 = 0.1067, wR_2 = 0.3058
Final R indexes [all data]	R_1 = 0.1407, wR_2 = 0.3345
Largest diff. peak/hole / e Å^{-3}	0.54/-0.58
Flack parameter	0.08(3)

$R_1 = \sum||F_O| - |F_C|| / \sum|F_O|$; $wR_2 = [\sum w(F_O^2 - F_C^2)^2 / \sum w(F_O^2)^2]^{1/2}$; $GooF = [\sum w(F_O^2 - F_C^2)^2 / (N_{ref} - N_{par})]^{1/2}$

Experimental and Refinement Details: [TBuA][Co(dca)$_3$] (Orthorhombic)

(CCDC Deposition Number 2026497)

The experiment was performed on a pink, prismatic crystal of [TBuA][Co(dca)$_3$] (0.14× 0.10 × 0.10 mm^3). The crystal was placed in a 0.1 mm diameter polyamide loop and mounted on a Bruker four circle kappa-diffractometer equipped with a Cu INCOATED microsource, operated at 30 W power (45 kV, 0.60 mA) to generate Cu Kα radiation (λ = 1.54178 Å), and a Bruker VANTEC 500 area detector (microgap technology) at 293(2) K. Diffraction data were collected over reciprocal space in a combination of φ and ω scans to reach a resolution of 0.85 Å, with a completeness > 95%, and redundancy >3. For this, either a generic hemisphere collection strategy or a specific one determined using Bruker APEX3 software suite was used. The exposure time was adjusted based on the size and diffracting quality of the specimen, each exposure covering 1° in ω or φ. Unit cell dimensions was determined for least-squares fit of reflections with I>4σ. The structure was solved by direct methods implemented in SHELX package in the orthorhombic $P2_12_12$ space group with Z = 8. This crystal structure is complicated by disorder, both in the dca ligands and in the TBuA cations.[1] In addition, this structure was solved on the basis of diffraction data from pseudomerohedrally twinned crystals applying the twin law: (-1.0,0.0, 0.0; -1.0, 0.0, 0.0; 0.0, 0.0, -1.0). The hydrogen atoms were fixed at their calculated positions using distances and angle constraints. All calculations were performed using APEX3 software for data collection and OLEX2-1.2[2] and SHELXTL[3] to resolve and refine the structure. Modelling of electron density led to the identification of an asymmetric unit (atom in crystallographic independent positions) with one $(N_2C_3)_2$ structural unit at the site A and one $Co_4N_{18}C_{12}$ structural unit at the site B. There was no significant residual density indicating the presence of solvent molecules in the structure. The structural unit in site A is disordered between two slightly different configurations with complementary occupancies. All non-hydrogen atoms in the structure were refined anisotropically. Final refinement was executed in 80 cycles and the final flux matrix F^2 converged to R_1 = 0.1093 (F_o > 4σF_o) and wR_2 = 0.2569 (all data), with GooF = 1.039.

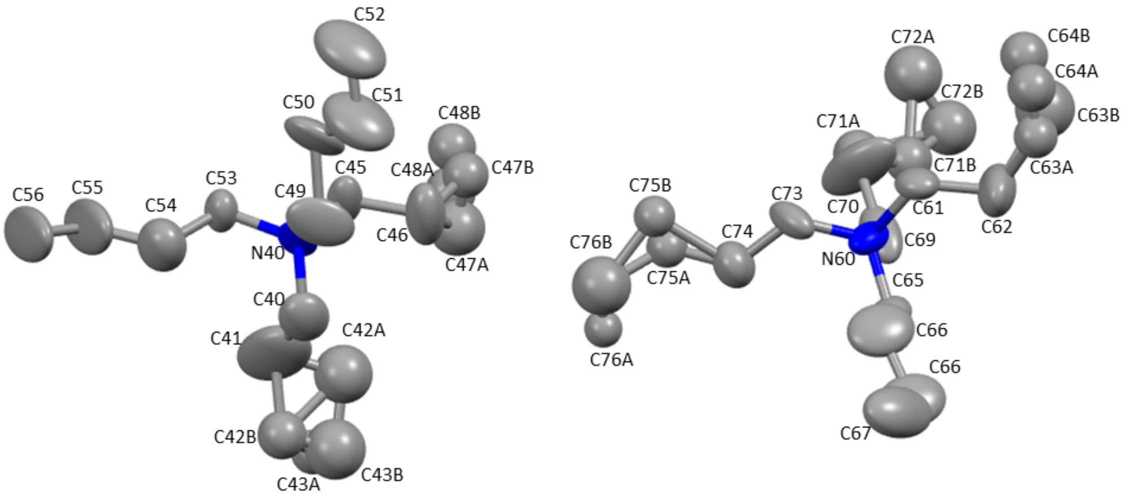

Figure S3. Graphical representation using thermal ellipsoids of the [TBuA]$^+$ cation associated with N40 and N60 in compound [TBuA][Co(dca)$_3$], integrated by two NC_{16} structural unit at the site A. Carbon atoms exhibit positional disorder. Hydrogen atoms are omitted for clarity.

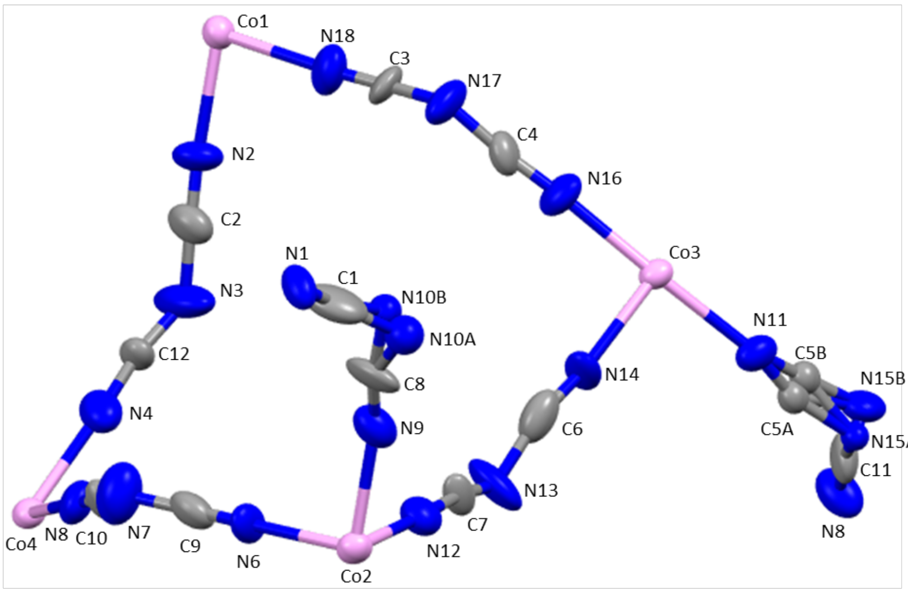

Figure S4. Graphical representation using thermal ellipsoids of the $Co(dca)_3^-$ anion in compound [TBuA][Co(dca)$_3$], integrated by $Co_4N_{18}C_{12}$ structural unit at the site B. Carbon atoms exhibit positional disorder. Hydrogen atoms are omitted for clarity.

Table S2. Crystal data and structure refinement details for [TBuA][Co(dca)$_3$].

Empirical formula	$C_{22}H_{36}CoN_{10}$
Formula weight	499.01
Temperature/K	296.15
Crystal system	orthorhombic
Space group	$P2_12_12$
a/Å	15.7659(6)
b/Å	15.7712(5)
c/Å	21.2815(7)
α/°	90
β/°	90
γ/°	90
Volume/Å^3	5291.6(3)
Z	8
ρ_{calc}g/cm^3	1.253
μ/mm^{-1}	5.311
F(000)	2116.0
Crystal size/mm^3	0.2 × 0.2 × 0.2
Radiation	CuKα (λ = 1.54178)
2Θ range for data collection/°	4.152 to 130.74
Index ranges	$-18 \le h \le 16$, $-18 \le k \le 18$, $-24 \le l \le 25$
Reflections collected	54532
Independent reflections	8979 [R_{int} = 0.1251, R_{sigma} = 0.0954]
Data/restraints/parameters	8979/12/598
Goodness-of-fit on F^2	1.039
Final R indexes [I>=2σ (I)]	R_1 = 0.1094, wR_2 = 0.2333
Final R indexes [all data]	R_1 = 0.1507, wR_2 = 0.2569
Largest diff. peak/hole / e Å^{-3}	0.50/-0.60
Flack parameter	0.09(2)

$R_1 = \sum||F_O| - |F_C|| / \sum|F_O|$; $wR_2 = [\sum w(F_O^2 - F_C^2)^2 / \sum w(F_O^2)^2]^{1/2}$; $GooF = [\sum w(F_O^2 - F_C^2)^2 / (N_{ref} - N_{par})]^{1/2}$

Experimental and Refinement Details: [TPnA][Fe(dca)$_3$] (Orthorhombic)

(CCDC Deposition Number 2026508)

The experiment was performed on a colourless crystal of [TPnA][Fe(dca)$_3$] (0.11× 0.08 × 0.05 mm^3), with a tetrahedral shape. The crystal was placed in a 0.1 mm diameter borosilicate loop and mounted on a Gemini E Ultra diffractometer from Oxford Diffraction equipped with an Atlas CCD area detector, operating at 35 kV and 35 mA to generate Cu Kα radiation (λ = 1.54184 Å) at 293(2) K. A total of 6312 reflections were collected within a θ range from 4.03 – 64.15°. From those reflections, 2546 were unique and 2066 were greater than 2σ(I). According with the data analysis, experimental data decay was negligible during data collection. The structure was solved in the orthorhombic *Pnna* space group with $Z = 4$ using the SUPERFLIP method.[4] Modelling of electron density led to the identification of an asymmetric unit (crystallographic independent atoms) with one NC_{10} structural unit at the site A and one FeN_5C_3 structural unit at the site B. There was no indication of solvent molecules in the structure. The structural unit in site A is disordered between two slightly different configurations with complementary occupancies (55% and 45%). All non-hydrogen atoms in the structure were refined anisotropically. All hydrogen atoms were placed in geometrically located positions and their displacement parameters are tied to those of the attached carbon atoms. Final refinement was executed in 50 cycles and the final flux matrix F^2 converged to $R_1 = 0.0413$ ($F_o > 4\sigma F_o$)) and $wR_2 = 0.1174$ (all data), with GooF = 1.056.

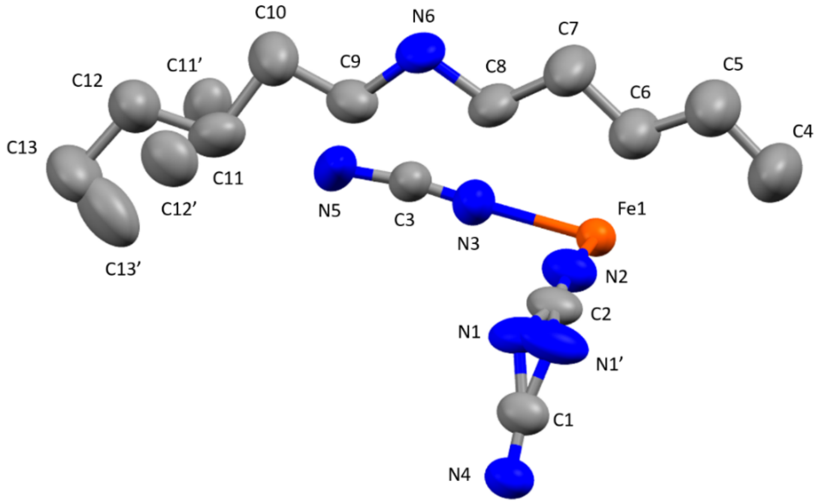

Figure S5. Graphical representation using thermal ellipsoids of the asymmetric unit of [TPnA][Fe(dca)$_3$], integrated by one NC_{10} structural unit at the site A and one FeN_5C_3 structural unit at the site B. Cations in sites A and B exhibit positional disorder. Hydrogen atoms are omitted for clarity.

Table S3. Crystal data and structure refinement details for [TPnA][Fe(dca)$_3$].

Empirical formula	$C_{26}H_{44}FeN_{10}$
Formula weight	552.56
Temperature/K	293 (2)
Crystal system	orthorhombic
Space group	Pnna
a/Å	13.1007(6)
b/Å	11.6128(5)
c/Å	20.0929(8)
α/°	90
β/°	90
γ/°	90
Volume/Å^3	3056.8(2)
Z	4
ρ_{calc}g/cm^3	1.201
μ/mm^{-1}	4.200
F(000)	1184.0
Crystal size/mm^3	0.11 × 0.08 × 0.05
Radiation	CuKα (λ = 1.54184)
2Θ range for data collection/°	8.056 to 128.754
Index ranges	$-15 \leq h \leq 14$, $-13 \leq k \leq 13$, $-15 \leq l \leq 23$
Reflections collected	6974
Independent reflections	2546 [R_{int} = 0.0250, R_{sigma} = 0.0253]
Data/restraints/parameters	2546/6/207
Goodness-of-fit on F^2	1.055
Final R indexes [I>=2σ (I)]	R_1 = 0.0413, wR_2 = 0.1079
Final R indexes [all data]	R_1 = 0.0524, wR_2 = 0.1161
Largest diff. peak/hole / e Å^{-3}	0.26/-0.23

$R_1 = \sum||F_O| - |F_C|| / \sum|F_O|$; $wR_2 = [\sum w(F_O^2 - F_C^2)^2 / \sum w(F_O^2)^2]^{1/2}$; $GooF = [\sum w(F_O^2 - F_C^2)^2 / (N_{ref} - N_{par})]^{1/2}$

Experimental and Refinement Details: [TPnA][Co(dca)$_3$] (Orthorhombic)

(CCDC Deposition Number 2026496)

The experiment was performed on a pink crystal of [TPnA][Co(dca)$_3$] (0.10×0.08×0.06 mm^3), with a tetrahedral shape. The crystal was placed in a 0.2 mm diameter borosilicate loop and mounted on a Gemini E Ultra diffractometer from Oxford Diffraction equipped with an Atlas CCD area detector, operating at 35 kV and 35 mA to generate Cu Kα radiation (λ = 1.54184 Å) at 293(2) K. A total of 2570 reflections were collected in a θ range from 3.90 - 63.52°. From those reflections, 1720 were unique and 1573 were greater than 2σ(I). Experimental data decay during data collection was negligible, according with the data analysis. The structure was solved in the orthorhombic *Pnna* space group with $Z = 4$ using the SUPERFLIP method.[4] Modelling of electron density led to the identification of an asymmetric unit (atom in crystallographic independent positions) with one NC_{10} structural unit at the site A and one CoN_5C_3 structural unit at the site B. There was no significant residual density indicating the presence of solvent molecules in the structure. The structural unit in site A is disordered between two slightly different configurations with complementary occupancies (61% and 39%). All non-hydrogen atoms in the structure were refined anisotropically. All hydrogen atoms were placed in geometrically located positions and their displacement parameters are tied to those of the attached carbon atoms. Final refinement was executed in 50 cycles and the final flux matrix F^2 converged to $R_1 = 0.0623$ ($F_o > 4\sigma F_o$)) and $wR_2 = 0.1949$ (all data), with GooF = 1.147.

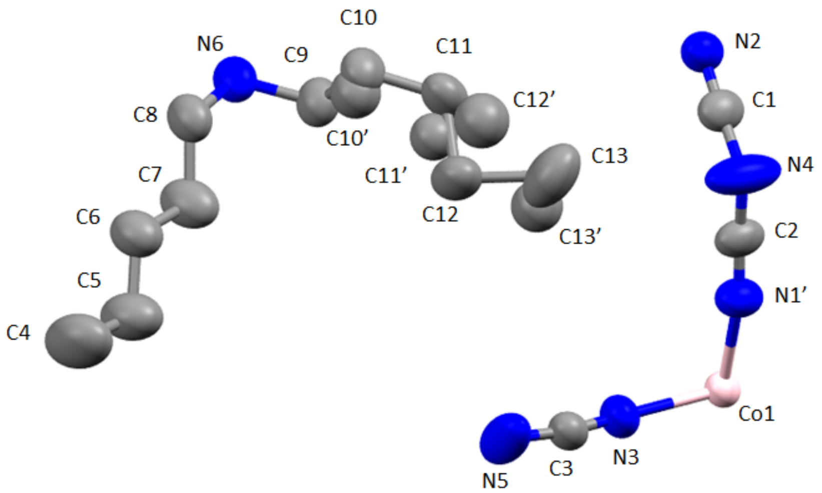

Figure S6. Graphical representation using thermal ellipsoids of the asymmetric unit of [TPnA][Co(dca)$_3$] integrated by one NC_{10} structural unit at the site A and one CoN_5C_3 structural unit at the site B. The molecular unit in site A is disordered between two complementary different orientations. All hydrogen atoms are omitted for clarity.

Table S4. Crystal data and structure refinement details for $[TPnA][Co(dca)_3]$.

Empirical formula	$C_{10}H_{20}Co_{0.5}N_8$
Formula weight	281.80
Temperature/K	293(2)
Crystal system	orthorhombic
Space group	Pnna
a/Å	13.066(2)
b/Å	11.6026(19)
c/Å	19.926(4)
α/°	90
β/°	90
γ/°	90
Volume/Å^3	3020.8(9)
Z	8
ρ_{calc}g/cm^3	1.239
μ/mm^{-1}	4.766
F(000)	1196.0
Crystal size/mm^3	0.10 × 0.08 × 0.06
Radiation	CuKα (λ = 1.54184)
2Θ range for data collection/°	8.092 to 128.338
Index ranges	$-13 \le h \le 15$, $-13 \le k \le 6$, $-23 \le l \le 19$
Reflections collected	6117
Independent reflections	2520 [R_{int} = 0.0448, R_{sigma} = 0.0543]
Data/restraints/parameters	2520/1/187
Goodness-of-fit on F^2	1.147
Final R indexes [I>=2σ (I)]	R_1 = 0.0623, wR_2 = 0.1730
Final R indexes [all data]	R_1 = 0.1078, wR_2 = 0.1949
Largest diff. peak/hole / e Å^{-3}	0.28/-0.20

$R_1 = \sum||F_O| - |F_C|| / \sum|F_O|$; $wR_2 = [\sum w(F_O^2 - F_C^2)^2 / \sum w(F_O^2)^2]^{1/2}$; $GooF = [\sum w(F_O^2 - F_C^2)^2 / (N_{ref} - N_{par})]^{1/2}$

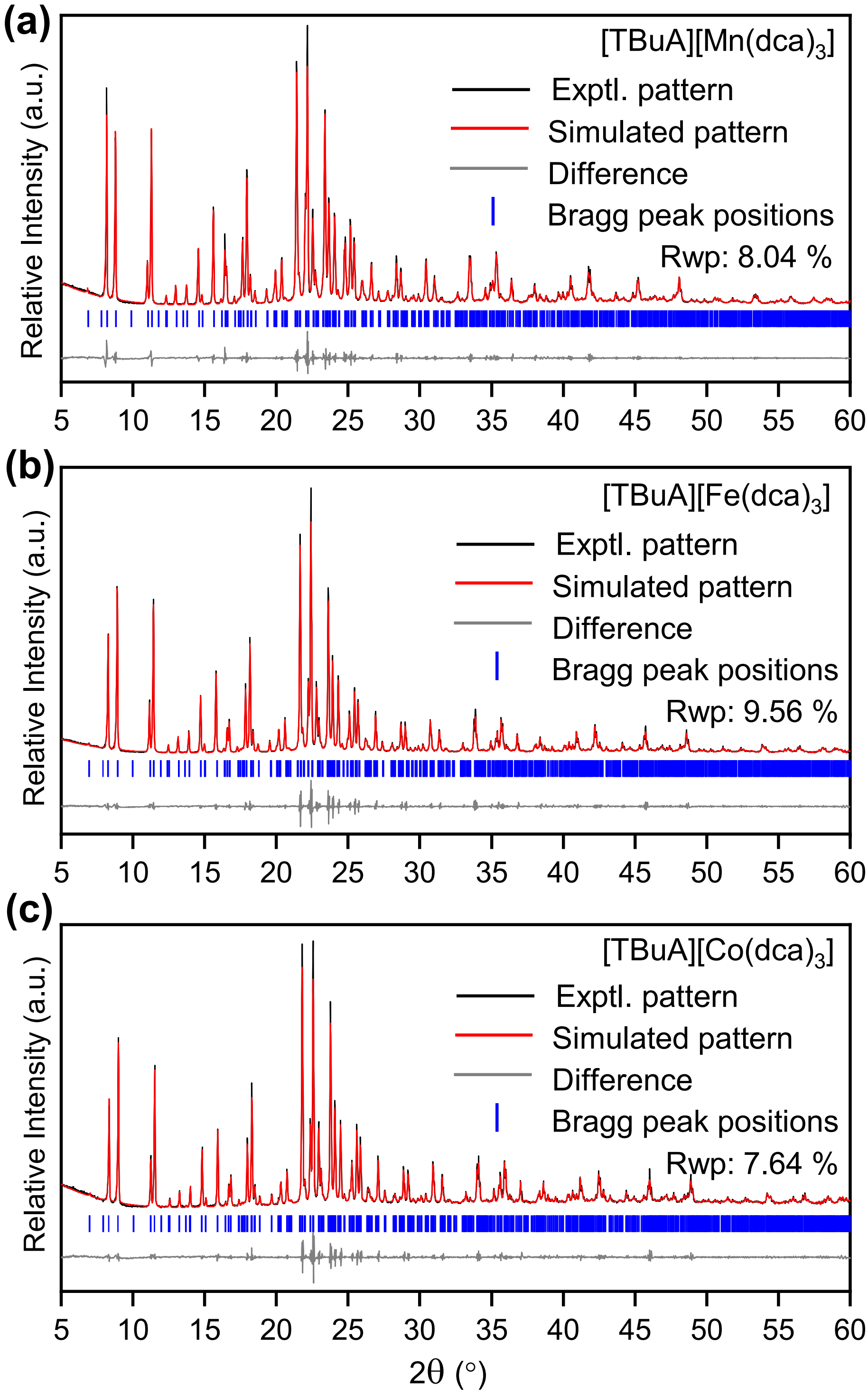

Figure S7. Pawley refinement of X-ray powder diffraction of [TBuA][M(dca)$_3$] data using TOPAS academic v6 software[5]. **a**, [TBuA][Mn(dca)$_3$], Reported cell parameters (Å): a = 16.0335 (5), b = 16.0150 (5), c = 21.5594 (4);[1] Refined cell parameters (Å): a = 16.0334, b = 16.0150, c = 21.5594. **b**, [TBuA][Fe(dca)$_3$], Experimental cell parameters (Å): a = 15.7971(7), b = 15.8093(9), c = 21.4138(11); Refined cell parameters (Å): a = 16.0034, b = 16.0050, c = 21.3594. **c**, [TBuA][Co(dca)$_3$], Experimental cell parameters (Å): a = 15.7659(6), b = 15.7712(5), c = 21.2815(7); Refined cell parameters (Å): a = 15.8034, b = 15.8050, c = 21.0594. Experimental cell parameters for **b** and **c** taken from single crystal X-ray diffraction data collected in this work.

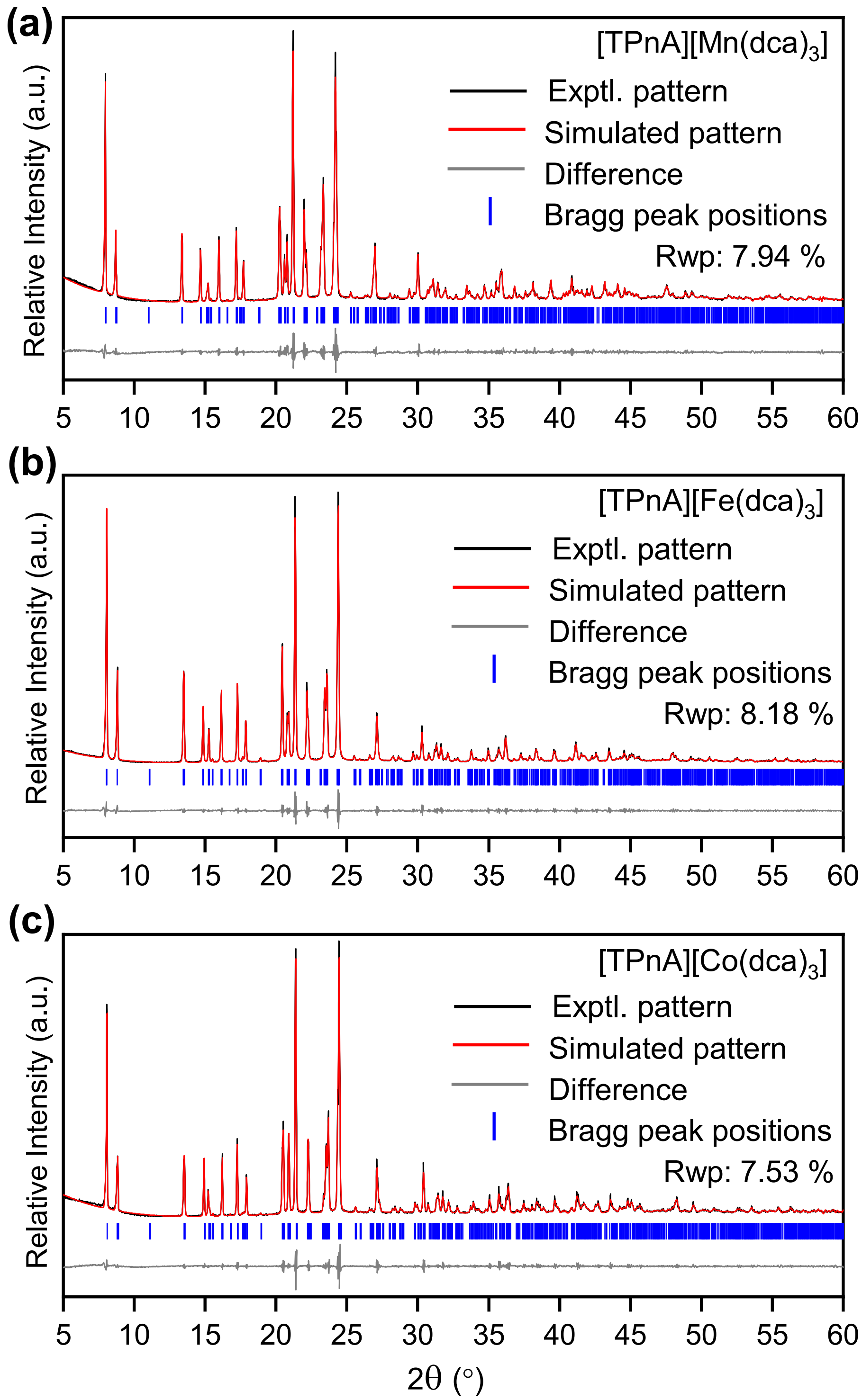

Figure S8. Pawley refinement of X-ray powder diffraction data of [TPnA][M(dca)$_3$] using TOPAS academic v6 software[5]. **a**, [TPnA][Mn(dca)$_3$], Reported cell parameters (Å): a = 13.2236(6), b = 11.6300(6), c = 20.3176(9);[1] Refined cell parameters (Å): a = 13.2227, b = 11.6403, c = 20.3254. **b**, [TPnA][Fe(dca)$_3$], Experimental cell parameters (Å): a = 13.1007(6), b = 11.6128(5), c = 20.0929(8); Refined cell parameters (Å): a = 13.2034, b = 11.5050, c = 20.0594. **c**, [TPnA][Co(dca)$_3$], Experimental cell parameters (Å): a = 13.066(2), b = 11.6026(9), c = 19.926(4); Refined cell parameters (Å): a = 13.2034, b = 11.5050, c = 20.0595. Experimental cell parameters for **b** and **c** taken from single crystal X-ray diffraction data collected in this work.

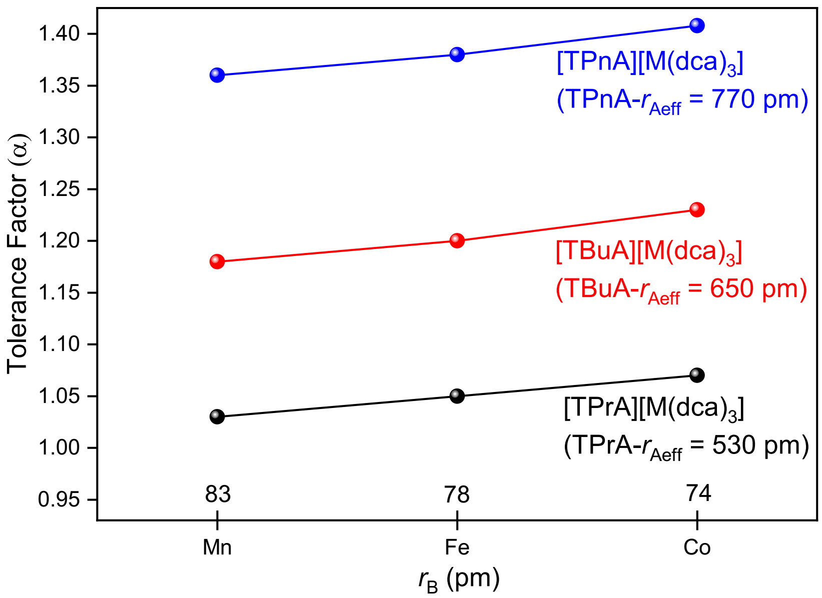

Figure S9: Values of ionic radius of 'B' metal is plotted with the calculated values of tolerance factor (α) for the current Mn-dicyanamide structures. Decrease of ionic radius of 'B' metal is found to increase the Tolerance factor slightly as per **equation (2)** given in main manuscript. For [TBuA][Fe(dca)$_3$] and [TBuA][Co(dca)$_3$] samples, the effective height of dca anion is taken as same as obtained in [TBuA][Mn(dca)$_3$].

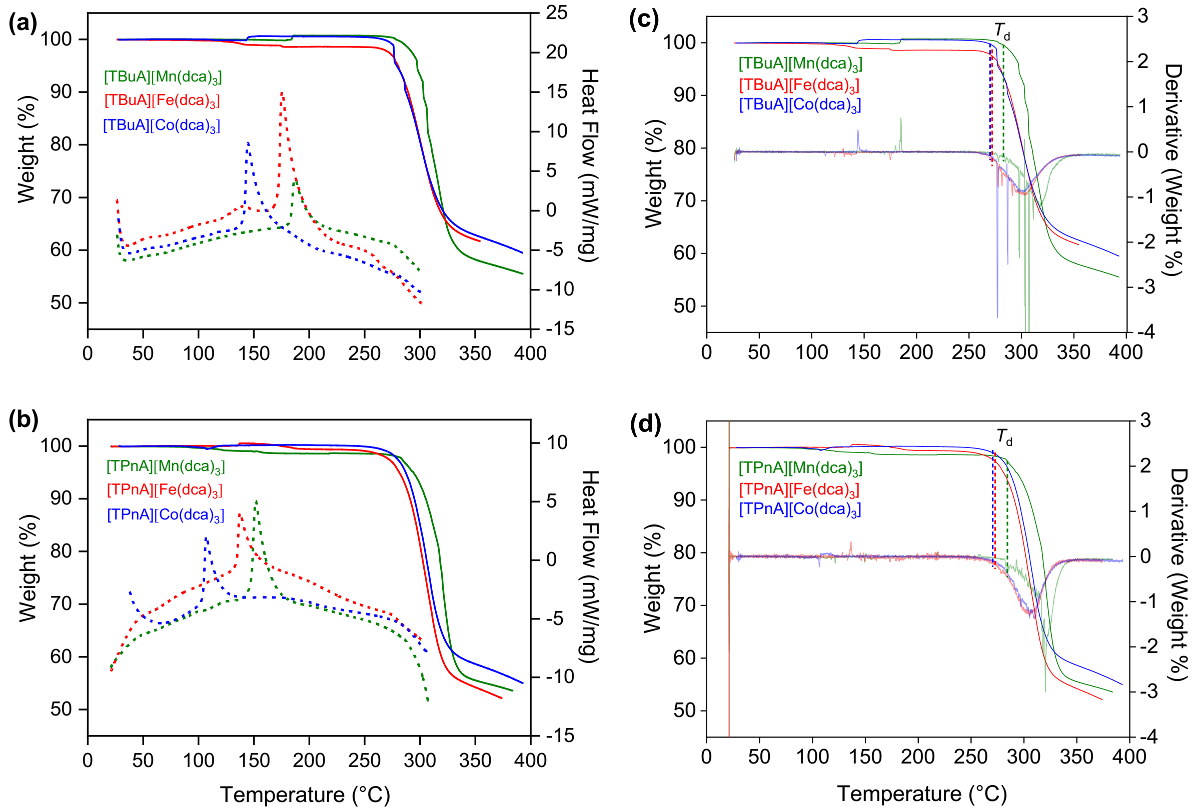

Figure S10. Change in weight % (solid lines, left axis) and corresponding change in heat flow (dashed lines, right axis) with temperature for **(a)** [TBuA][M(dca)$_3$] and **(b)** [TPnA][M(dca)$_3$] (M = Mn, Fe, Co), measured at a rate of 10 °C min^{-1} under an argon atmosphere. The absence of any mass loss at temperatures corresponding to the endotherms are indicative of melting in each case. Values of relative heat flow above 300 °C are omitted for clarity. Figures **(c)** and **(d)** show the temperature of decomposition for each case, evaluated from the change in the weight derivative (%) vs temperature. The dashed lines (in respective colours) represent the temperature at which decomposition (T_d) follows (282 °C for [TBuA][Mn(dca)$_3$], 271 °C for [TBuA][Fe(dca)$_3$], 271 °C for [TBuA][Co(dca)$_3$], 283 °C for [TPnA][Mn(dca)$_3$], 273 °C for [TPnA][Fe(dca)$_3$], 272 °C for [TPnA][Co(dca)$_3$]).

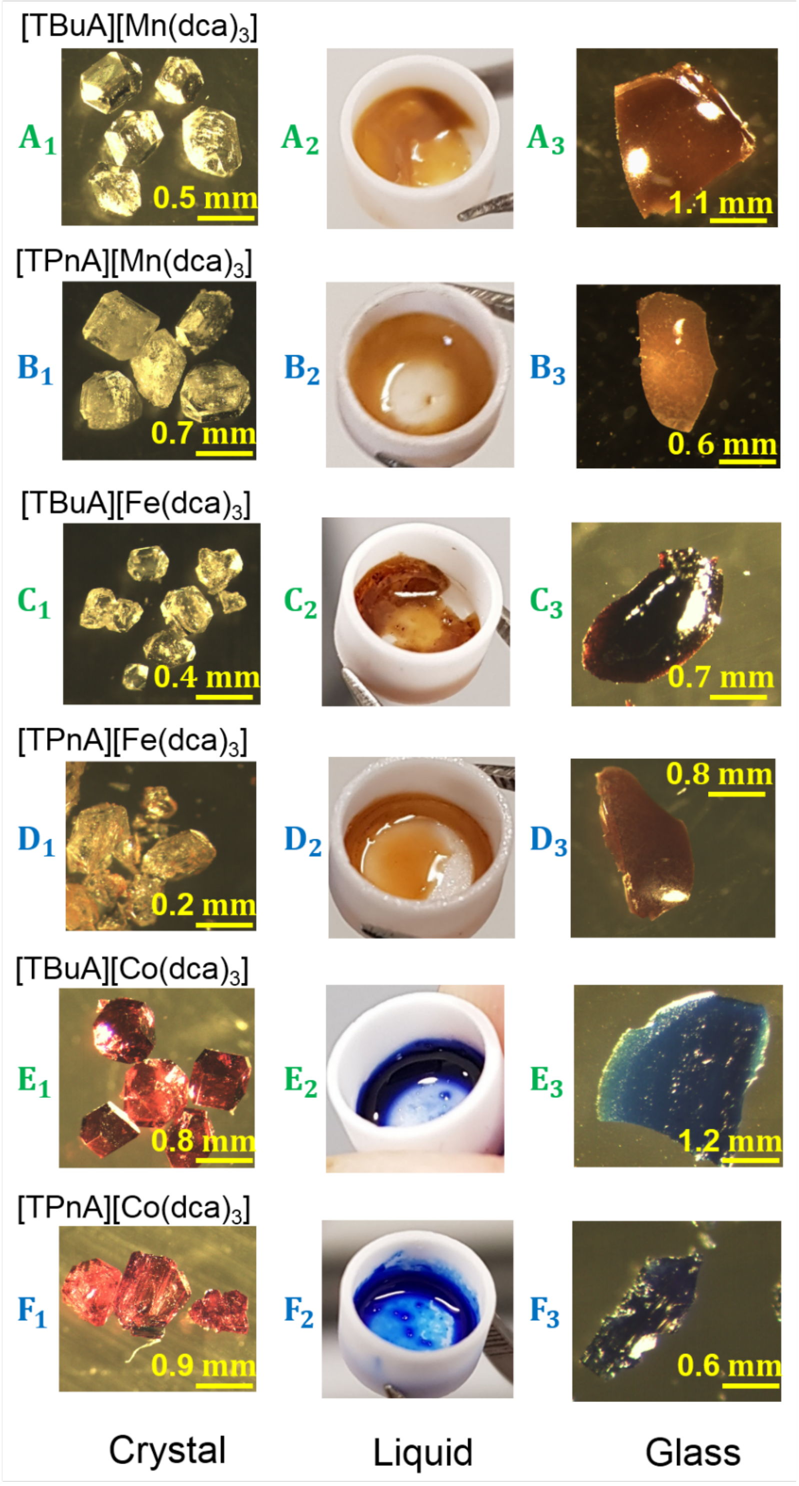

Figure S11. Optical images of the [TAlA][M(dca)$_3$] samples (where TAlA (tetraalkylammonium) = TBuA, TPnA and M = Mn, Fe, Co) prior to melting, immediately after melting and the quenched glasses formed using the methods described in the main manuscript. Numbered symbols, A-F indicate the transformation from crystal (1) to molten liquid (2) to quenched-glass (3) (see 'Preparation of glasses' for detail). Melting was performed in 70-μL alumina crucibles in an Argon atmosphere above their offset temperatures (images were taken instantly after opening the furnace at high temperature).

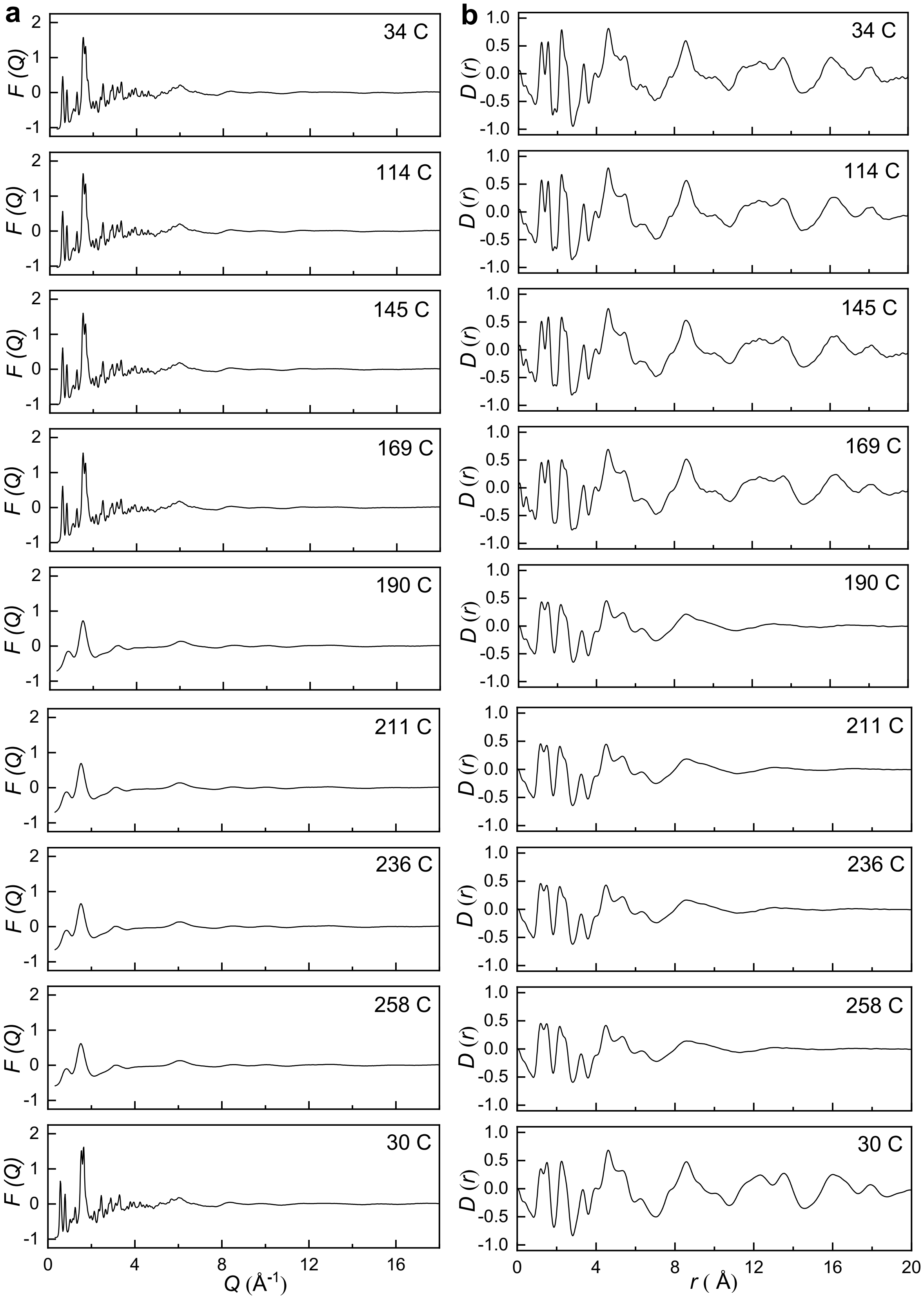

Figure S12. Variable temperature X-ray total scattering data of [TBuA][Mn(dca)$_3$] from 34 °C – 258 °C. **(a)** Structure factors, *F*(*Q*) and corresponding **(b)** Pair distribution functions, *D*(*r*). The absence of sharp features in the *F*(*Q*) at 190 °C clearly indicates melting and liquid formation. Sharp features appeared upon cooling from 258 °C indicate recrystallization as evidenced in DSC (**Fig. S23**).

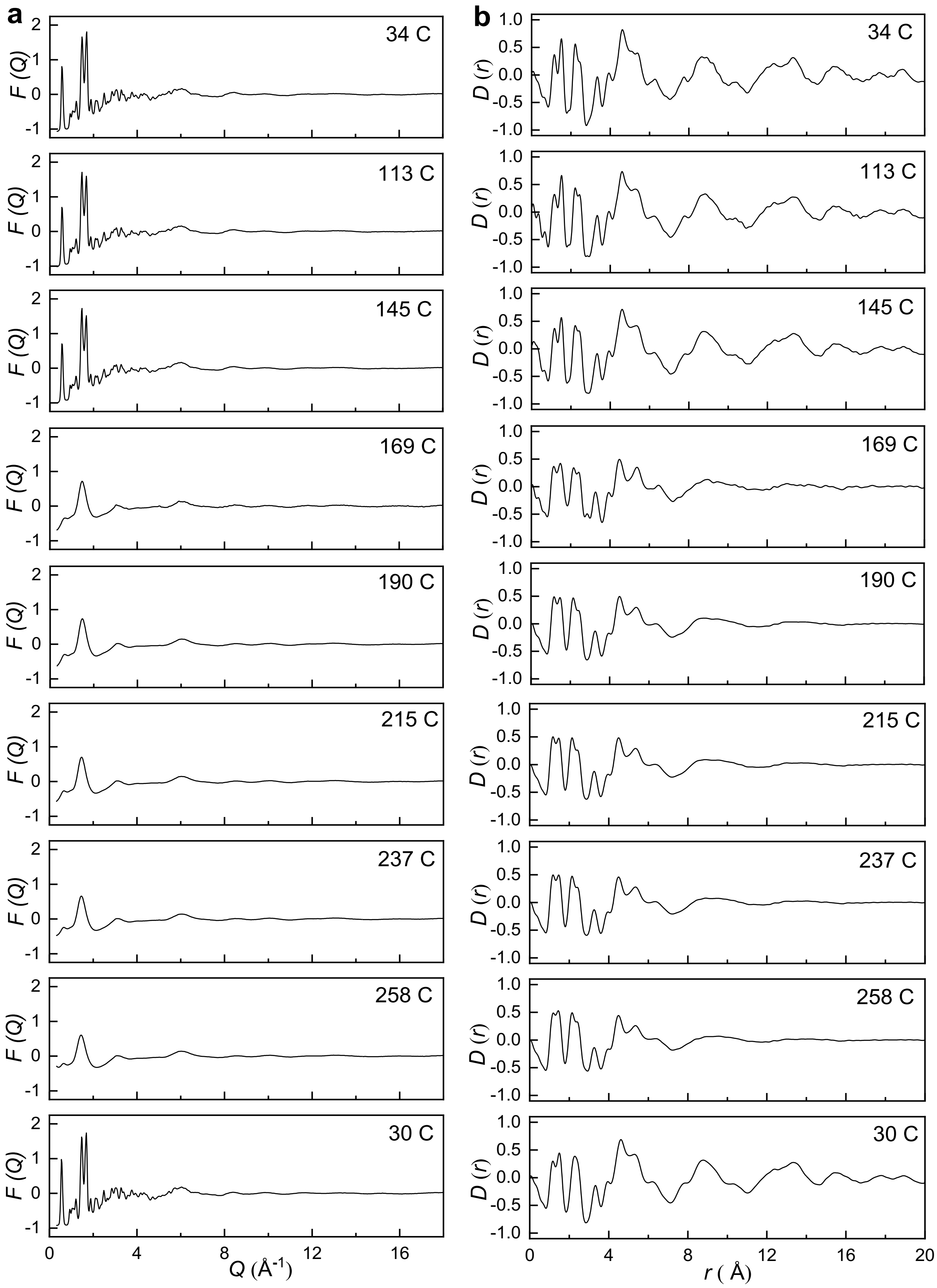

Figure S13. Variable temperature X-ray total scattering data of [TPnA][Mn(dca)$_3$] from 34 °C – 258 °C. **(a)** Structure factors, $F(Q)$ and corresponding **(b)** Pair distribution functions, $D(r)$. The absence of sharp features in the $F(Q)$ at 169 °C clearly indicates melting and liquid formation. Sharp features appeared upon cooling from 258 °C indicate recrystallization as evidenced in DSC (**Fig. S23**).

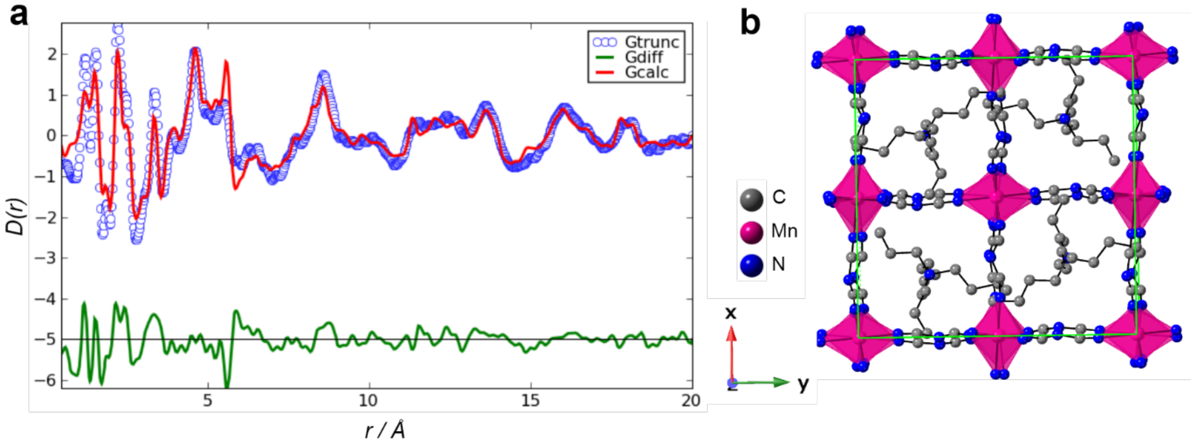

Figure S14. Structural Refinement of [TBuA][Mn(dca)$_3$] using PDF data. (a) Calculated and experimental data, along with difference plot. Reported cell parameters (Å): a = 16.0335 (5), b = 16.0150 (5), c = 21.5594 (4);[1] Refined cell parameters (Å): a = 13.2227, b = 11.6403, c = 20.3254. Rw = 0.39. Red – calculated $D(r)$, Blue – experimental $D(r)$, green – difference. **(b)** Simplified unit cell of the refined structure. Hydrogen atoms not shown for clarity.

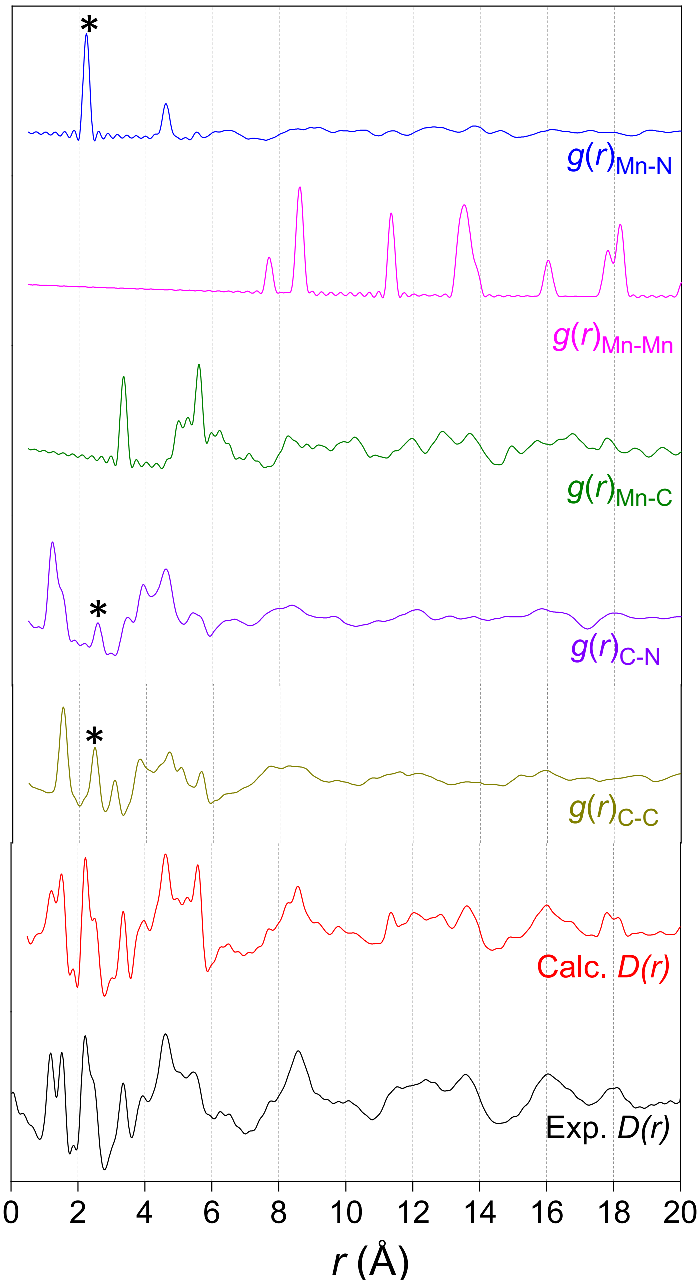

Figure S15. Comparison of calculated total and partial pair distribution functions for [TBuA][Mn(dca)$_3$]. These were calculated from PDFgui refinement of the published crystal structure.[1,6] Experimental $D(r)$ data in black. Black asterisks indicate the proximity of C-C and C-N correlations to the Mn-N correlation.

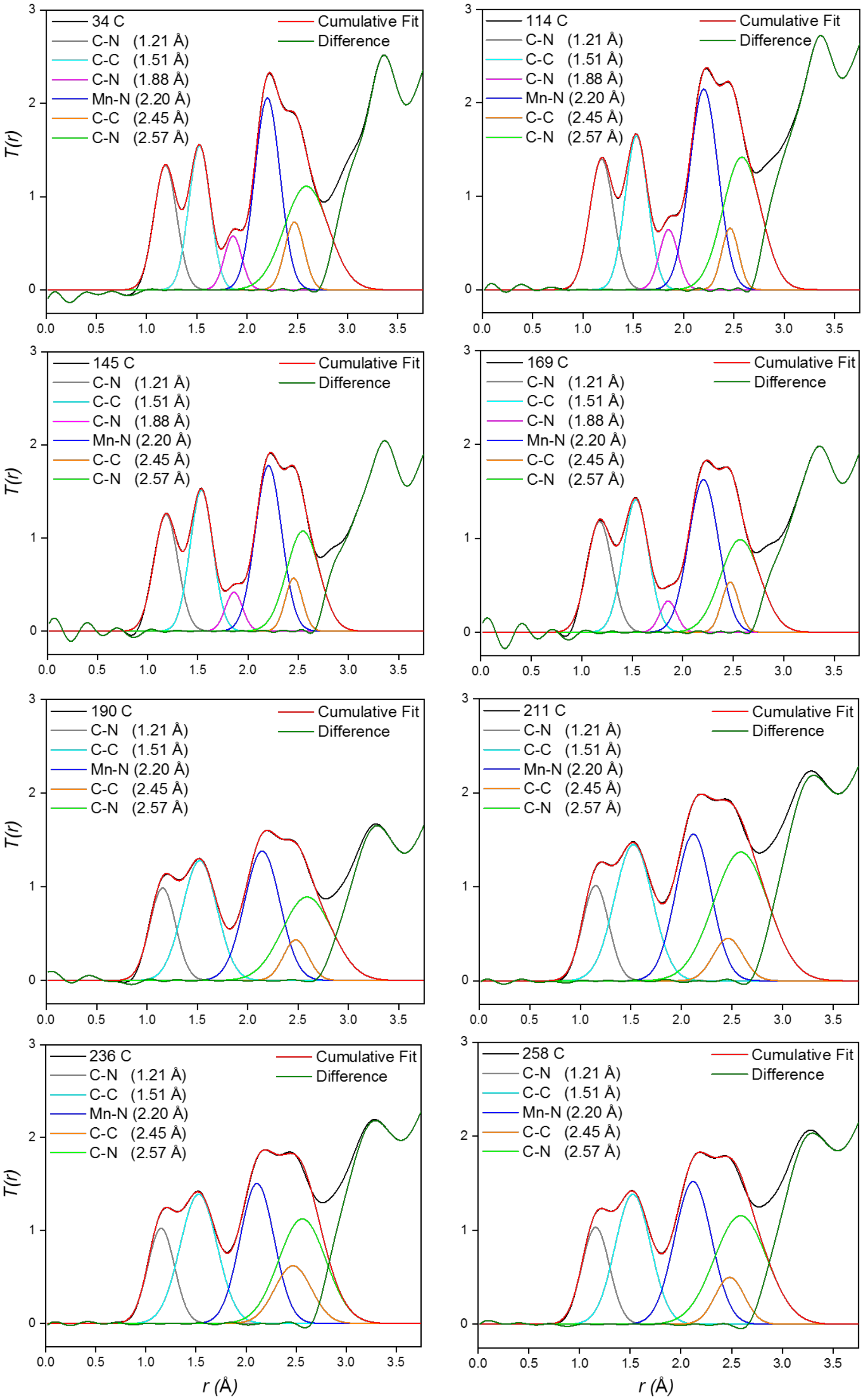

Figure S16. Regression analysis of Mn-N pair distribution function peak after background subtraction, $T(r)$ for [TBuA][Mn(dca)$_3$] at various temperatures. The proximity of C-C (2.45 Å) and C-N (2.57 Å) correlations to Mn-N (2.20 Å) correlation rendered it necessary to fit the experimental peak using multiple peak fitting, and extract the FWHM and d_0 values separately for Mn-N correlation (blue – fitted peak line). The parameters obtained from the peak fitting are summarized in **Table S6**.

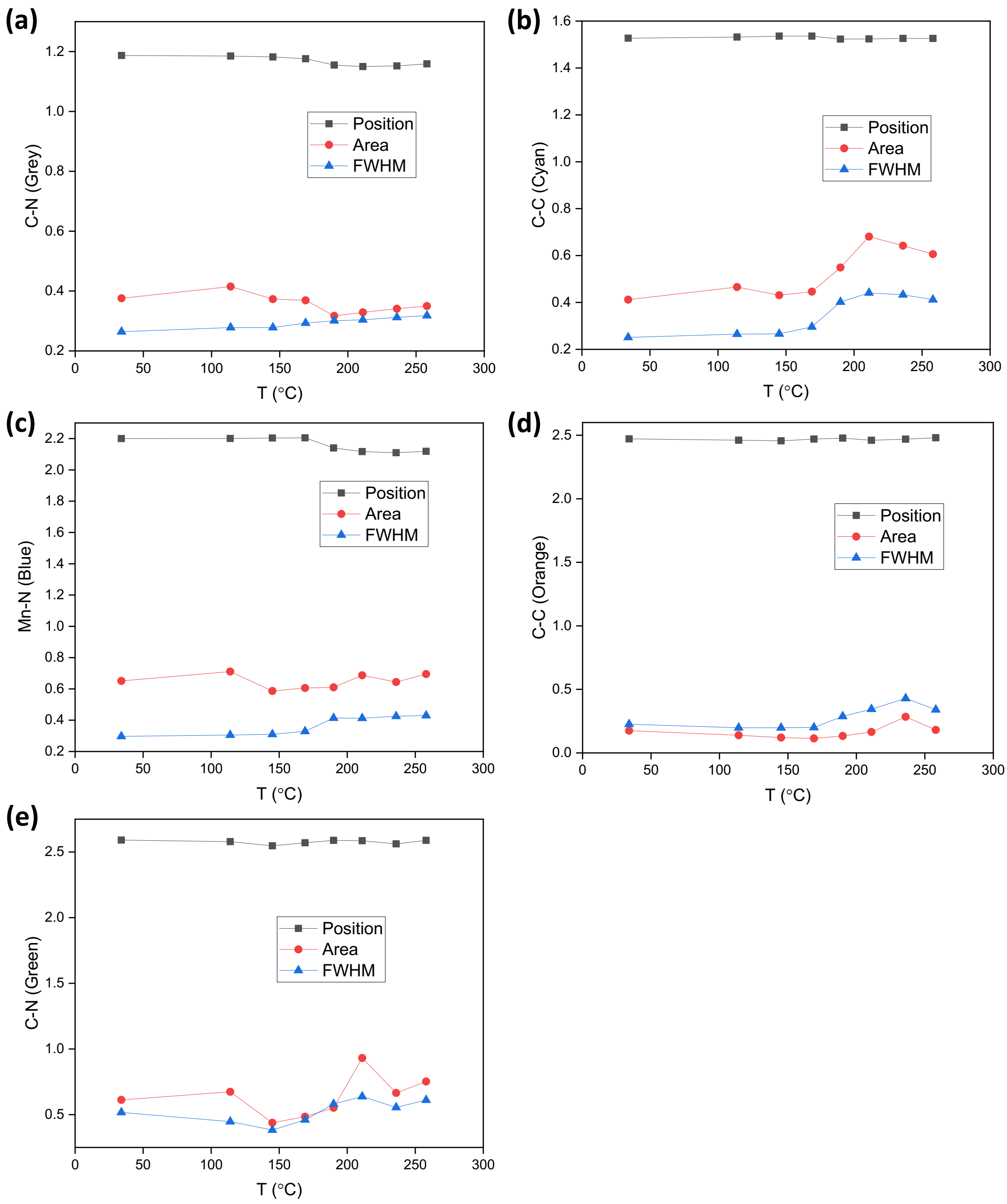

Figure S17. Deconvoluted peak positions, area and FWHM as a function of temperature. **(a)** C-N (terminal on dca linker): decreasing area indicating a portion of ligand decomposition. **(b)** C-C on quaternary amine in the A site – increased due to linker decomposition. **(c)** Mn-N coordination bond to dca linker. As we reported previously,[10] there is a decomposition of the dca linker to tricyclic amines, these will also be bonded to Mn so we would perhaps not have seen a big decrease. **(d)** Not a physical bond – distance between two adjacent carbons on both the dca linker, and also on the quaternary amine. **(e)** Not a physical bond – distance between non adjacent C and N atoms on 'A' site cation.

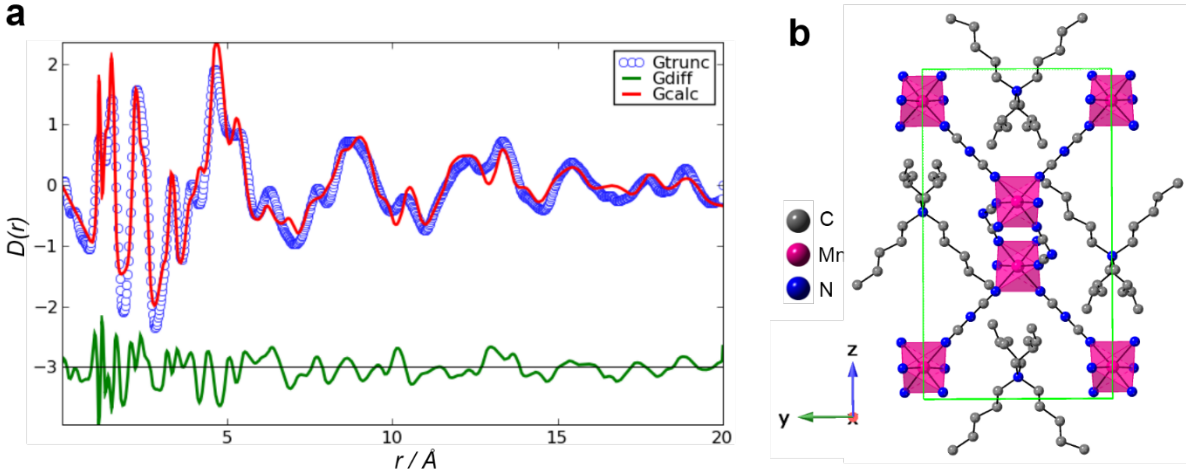

Figure S18. Structural Refinement of [TPnA][Mn(dca)$_3$] using PDF data. (a) Calculated and experimental data, along with difference plot. Reported cell parameters (Å): a = 13.2236(6), b = 11.6300(6), c = 20.3176(9);[1] Refined cell parameters (Å): a = 13.3107, b = 11.6768, c = 20.1817. Rw = 0.12. Red – calculated $D(r)$, Blue – experimental $D(r)$, green – difference. **(b)** Simplified unit cell of the refined structure.

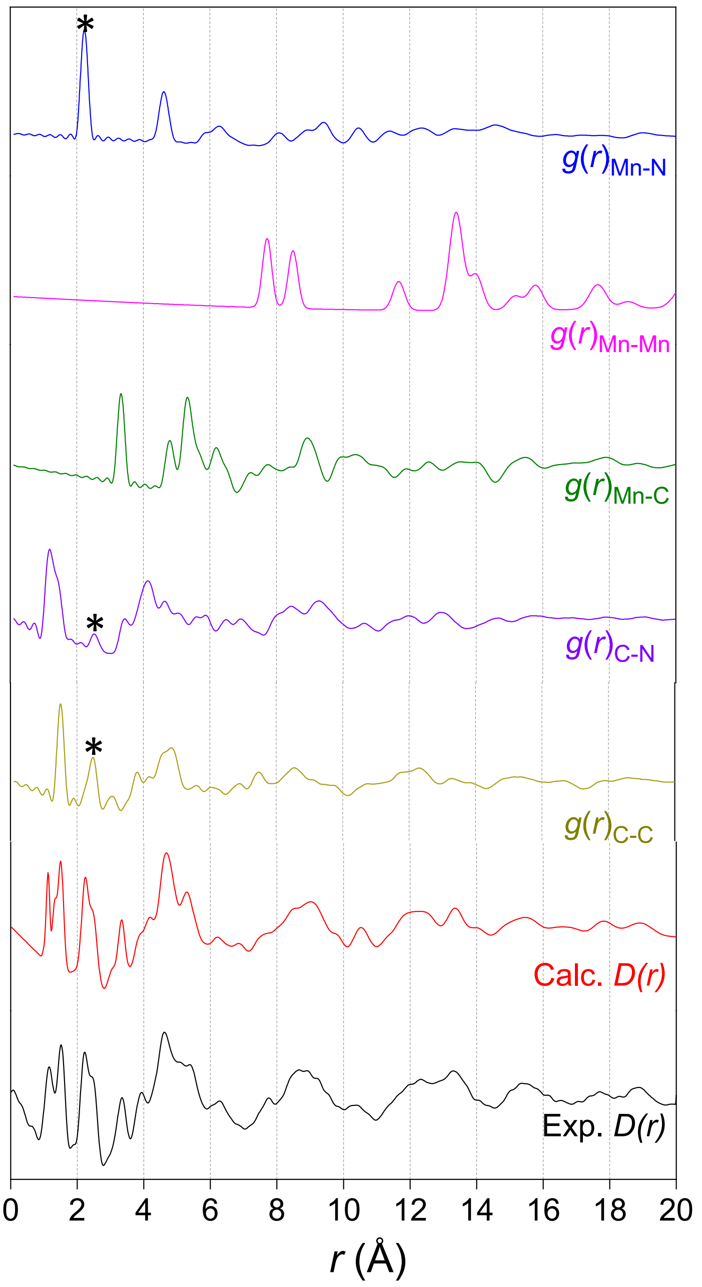

Figure S19. Comparison of calculated total and partial pair distribution functions for [TPnA][Mn(dca)$_3$]. These were calculated from PDFgui refinement of the published crystal structure.[1,6] Experimental $D(r)$ data in black. Black asterisks indicate the proximity of C-C and C-N correlations to the Mn-N correlation.

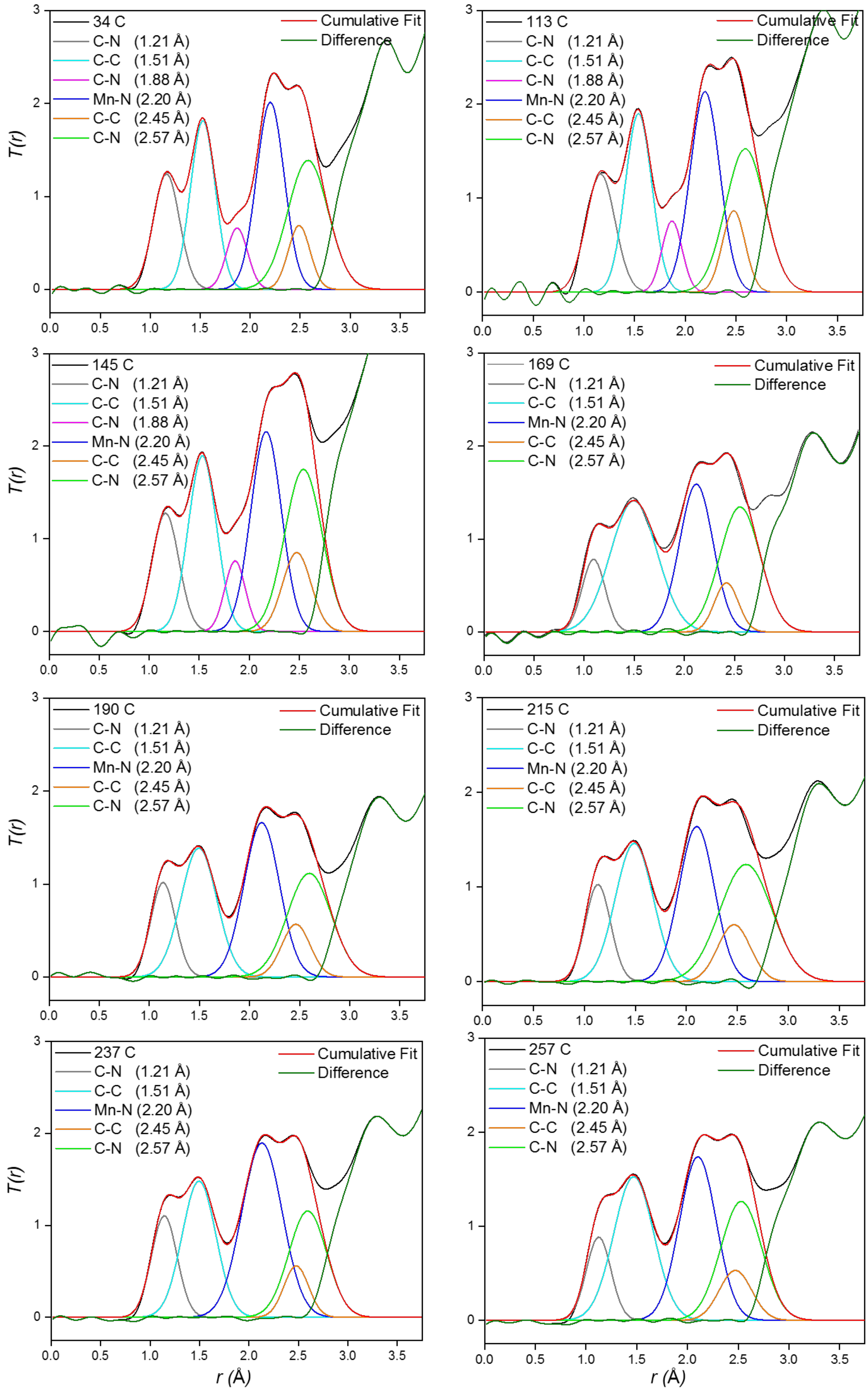

Figure S20. Regression analysis of Mn-N pair distribution function peak after background subtraction, $T(r)$ for [TPnA][Mn(dca)$_3$] at various temperatures. The proximity of C-C (2.45 Å) and C-N (2.57 Å) correlations to Mn-N (2.20 Å) correlation rendered it necessary to fit the experimental peak using multiple peak fitting, and extract the FWHM and d_0 values separately for Mn-N correlation (blue – fitted peak line). The parameters obtained from the peak fitting are summarized in **Table S6**.

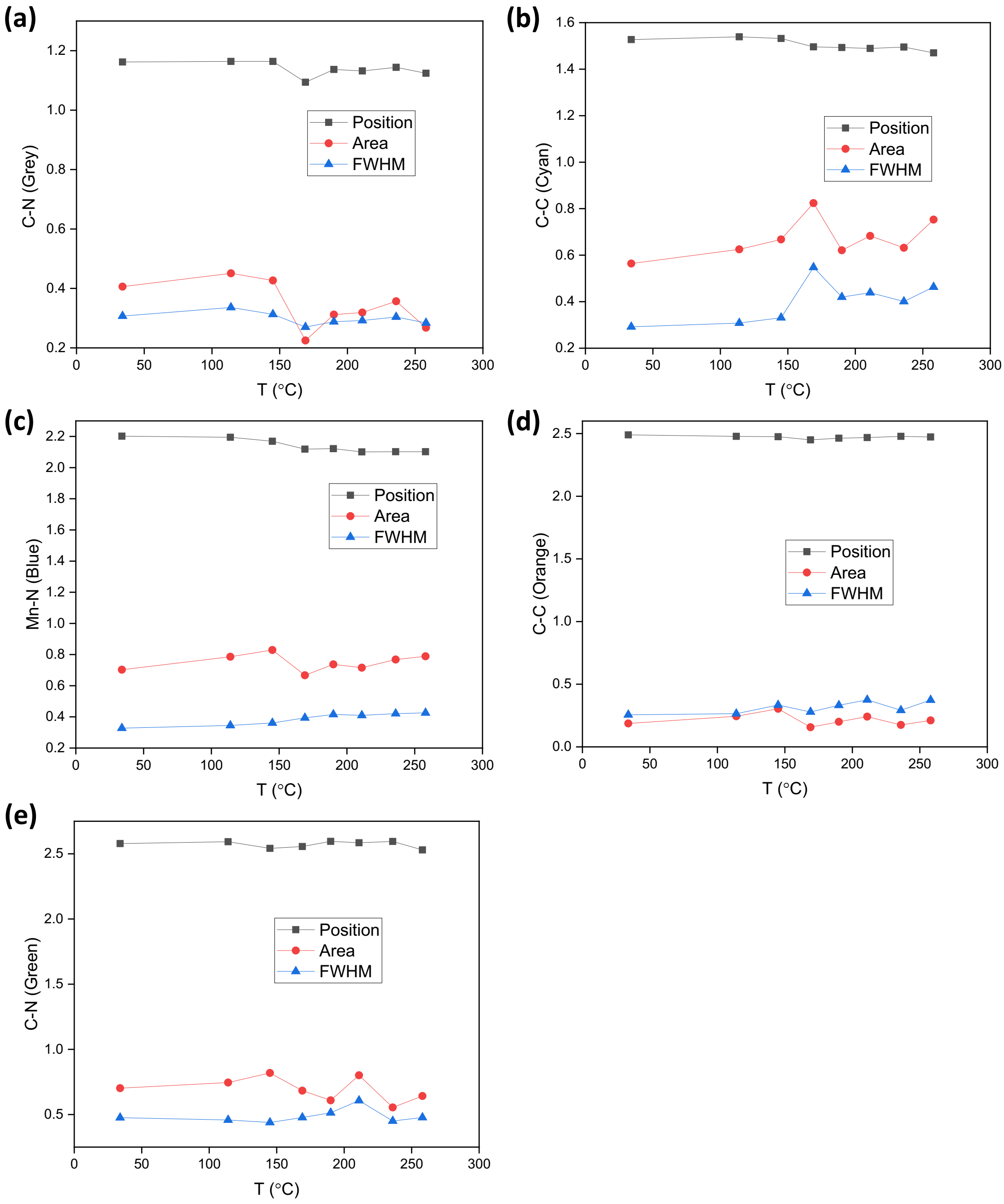

Figure S21. Deconvoluted peak positions, area and FWHM as a function of temperature. **(a)** C-N (terminal on dca linker): decreasing area indicating a portion of ligand decomposition. **(b)** C-C on quaternary amine in the A site – increased due to linker decomposition. **(c)** Mn-N coordination bond to dca linker. As reported previously,[10] there is a decomposition of the dca linker to tricyclic amines, these will also be bonded to Mn so a big change is perhaps not expected. **(d)** Not a physical bond – distance between two adjacent carbons on both the dca linker, and also on the quaternary amine. **(e)** Not a physical bond – distance between non adjacent C and N atoms on 'A' site cation.

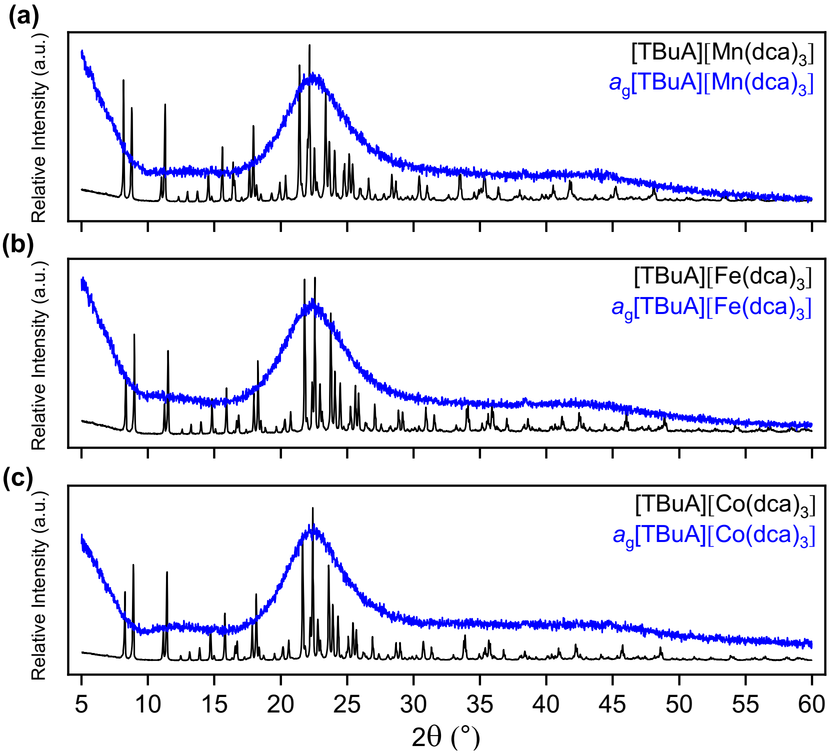

Figure S22. Ambient temperature powder X-ray diffraction patterns of [TBuA][M(dca)$_3$] (M = Mn, Fe, Co), before heating (black) and upon quenching from the liquid phase (blue). These samples correspond to the glasses shown in Supplementary Figure S10.

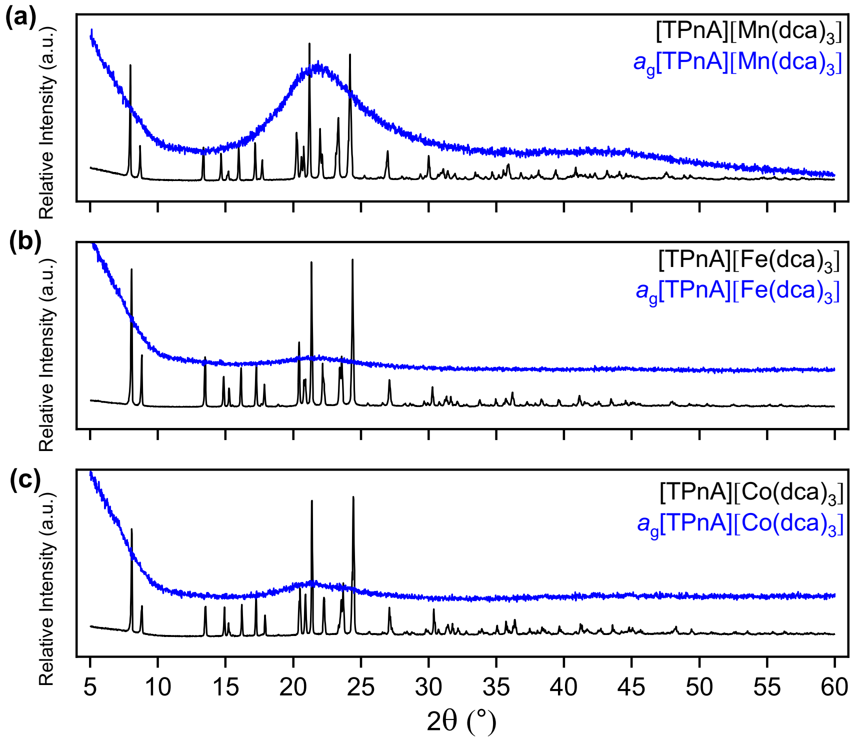

Figure S23. Ambient temperature powder X-ray diffraction patterns of [TPnA][M(dca)$_3$] (M = Mn, Fe, Co), before heating (black) and upon quenching from the liquid phase (blue). These samples correspond to the glasses shown in Supplementary Figure S10.

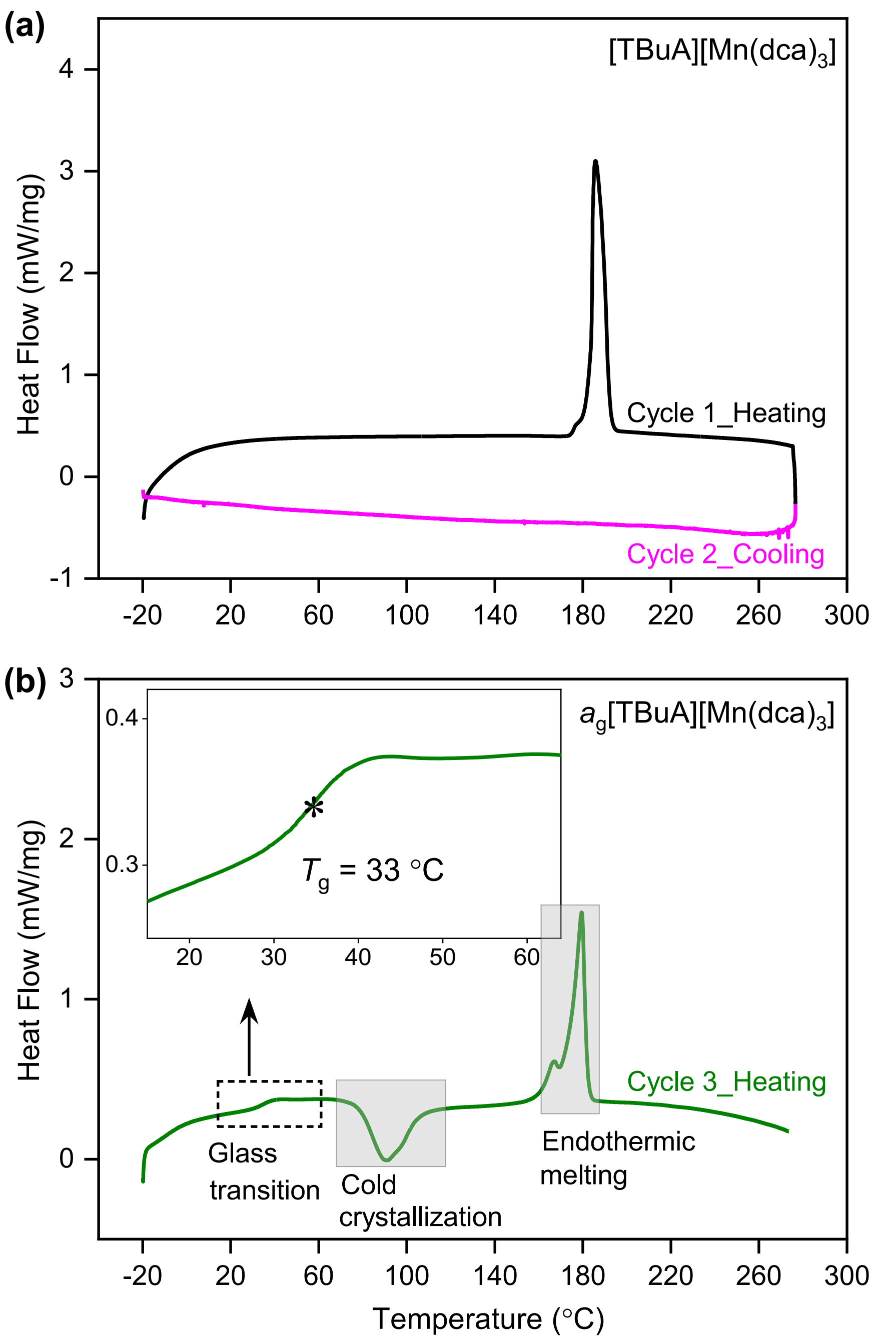

Figure S24: Change in heat flow as a function of temperature for **(a)** [TBuA][Mn(dca)$_3$] and **(b)** a_g[TBuA][Mn(dca)$_3$]. **(a)** Crystals were first heated above T_m at a rate of 10 °C min^{-1} under an argon atmosphere and then cooled to -20 °C at a rate of 3 °C min^{-1}. **(b)** The glass formed after quenching was then reheated at a rate of 10 °C min^{-1} to obtain the glass transition (T_g) which was identified (shown within black dashed line box) and marked with asterisk (zoomed and shown in inset). The glass also found to have cold crystallized and then melts at higher temperature (represented with sections under grey boxes).

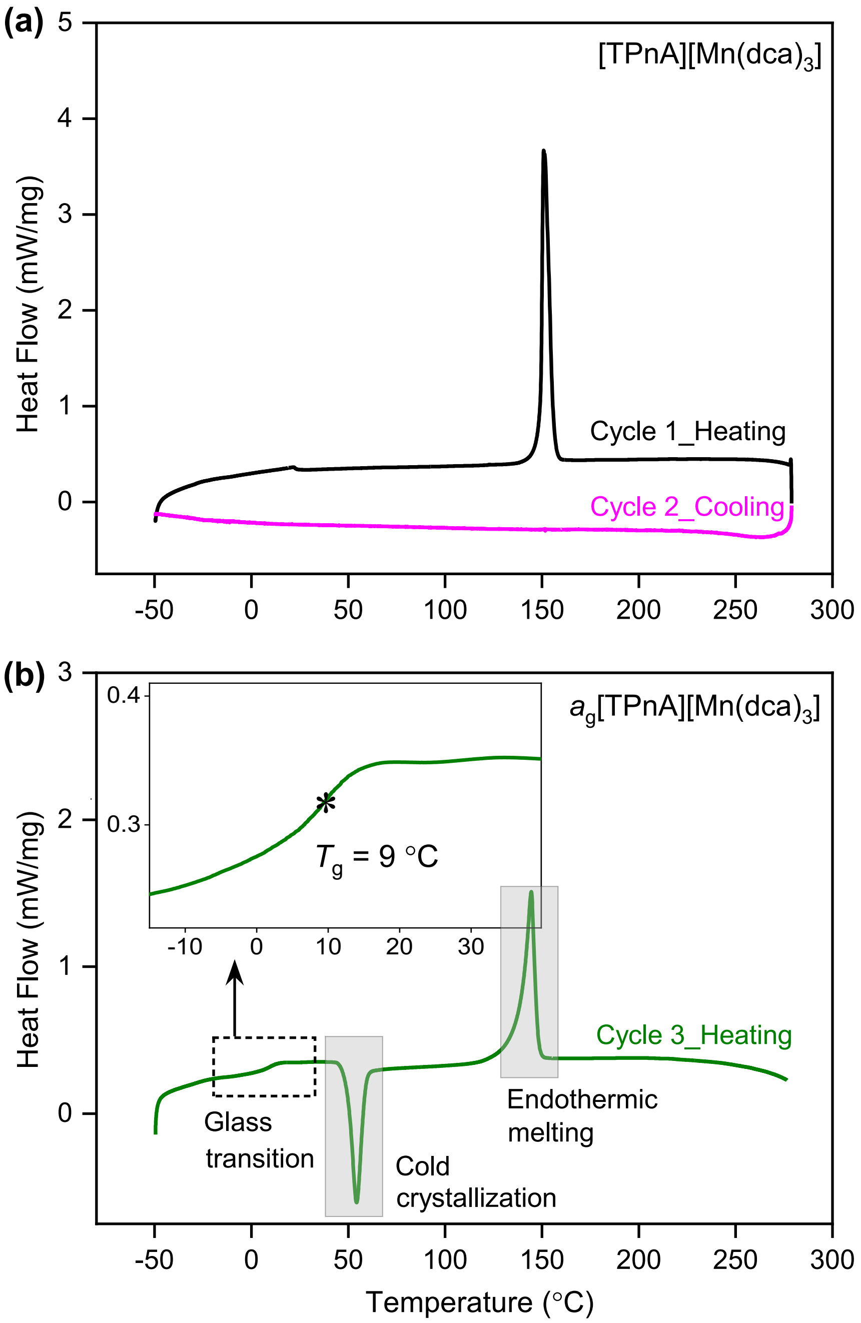

Figure S25: Change in heat flow as a function of temperature for **(a)** [TPnA][Mn(dca)$_3$] and **(b)** a_g[TPnA][Mn(dca)$_3$]. **(a)** Crystals were first heated above T_m at a rate of 10 °C min^{-1} under an argon atmosphere and then cooled to -50 °C at a rate of 3 °C min^{-1}. **(b)** The glass formed after quenching was then reheated at a rate of 10 °C min^{-1} to obtain the glass transition (T_g) which was identified (shown within black dashed line box) and marked with asterisk (zoomed and shown in inset). The glass also found to have cold crystallized and then melts at higher temperature (represented with sections under grey boxes).

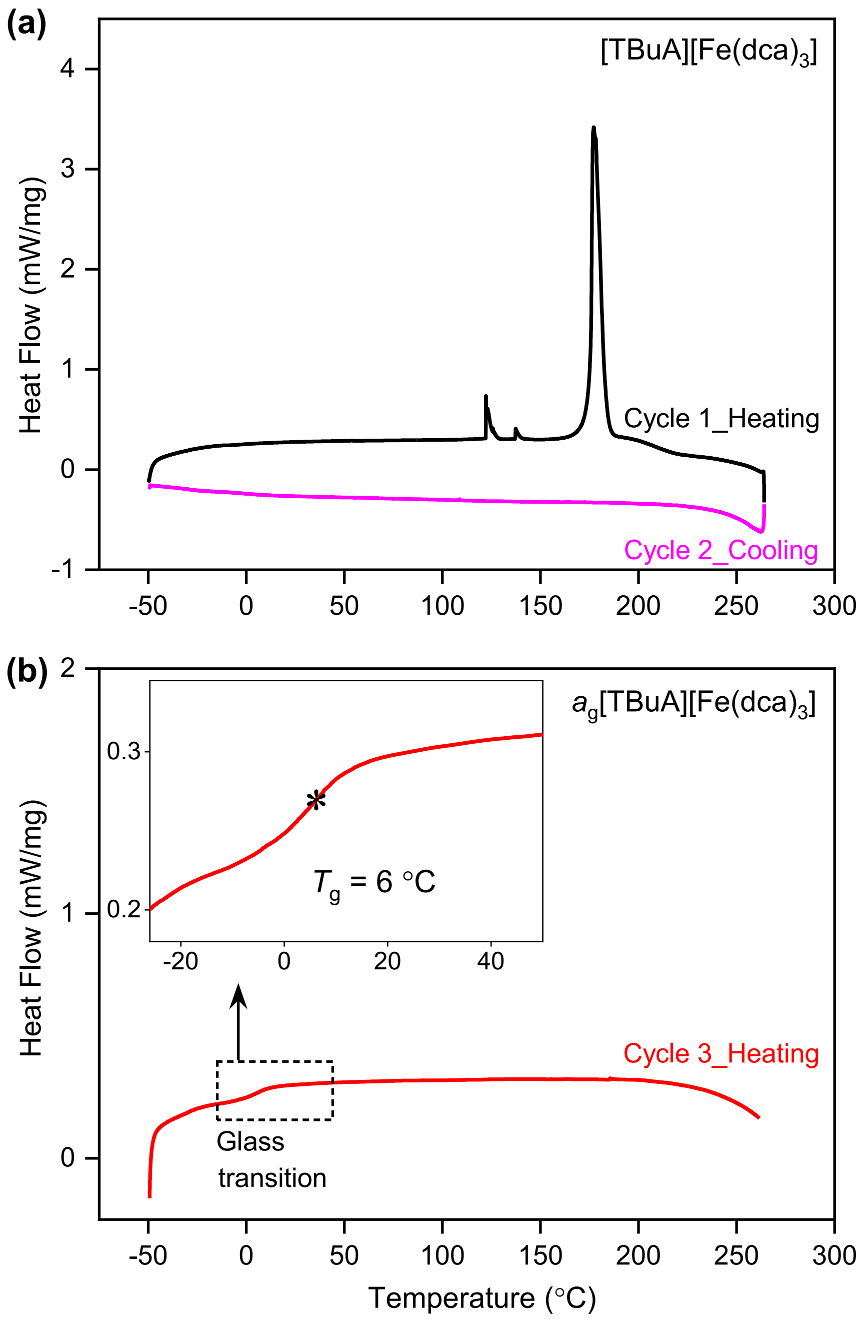

Figure S26: Change in heat flow as a function of temperature for **(a)** [TBuA][Fe(dca)$_3$] and **(b)** a_g[TBuA][Fe(dca)$_3$]. **(a)** Crystals were first heated above T_m at a rate of 10 °C min^{-1} under an argon atmosphere and then cooled to -50 °C at a rate of 5 °C min^{-1}. **(b)** The glass formed after quenching was then reheated at a rate of 10 °C min^{-1} to obtain the glass transition (T_g) which was identified (shown within black dashed line box) and marked with asterisk (zoomed and shown in inset).

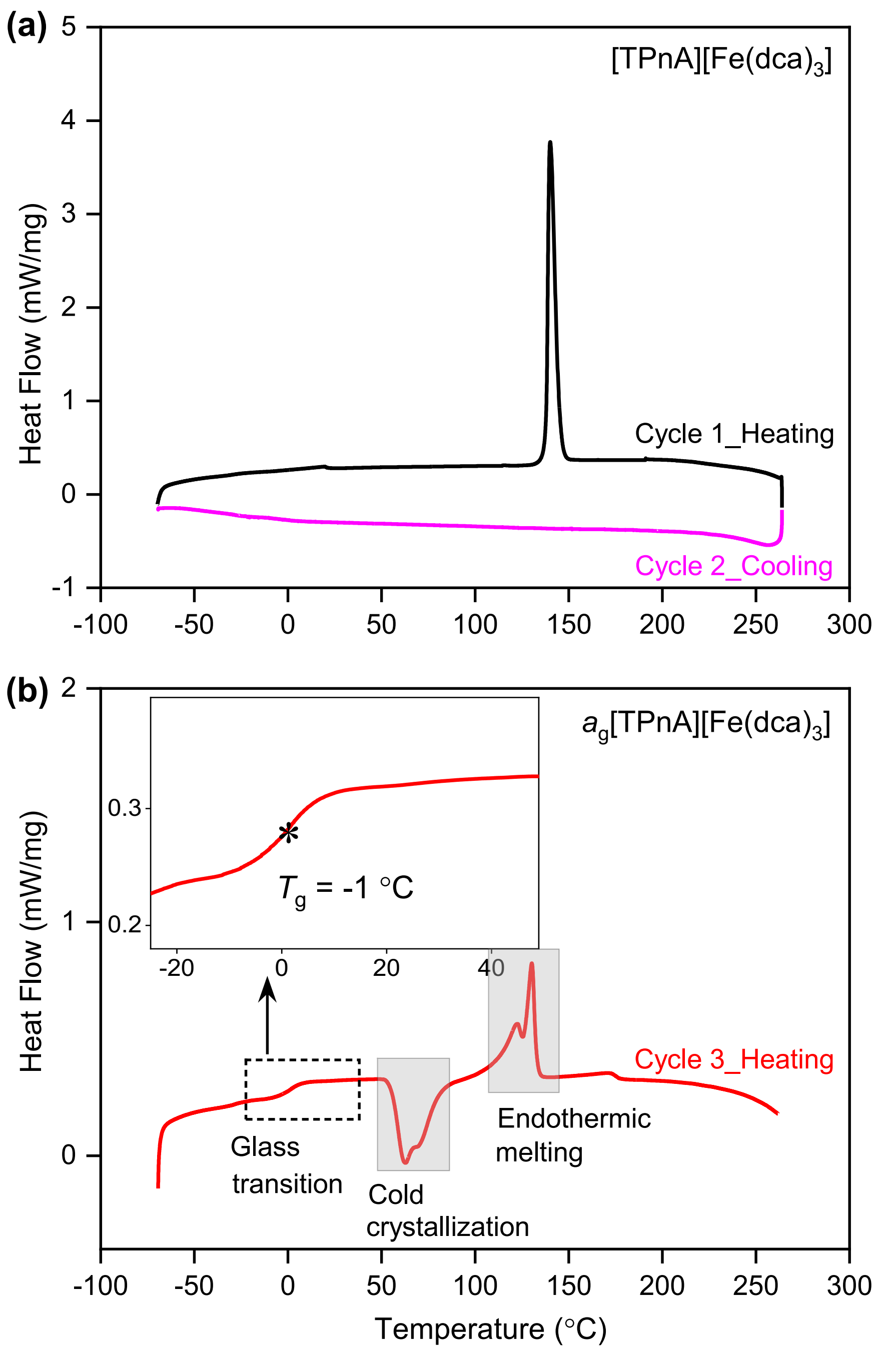

Figure S27: Change in heat flow as a function of temperature for **(a)** [TPnA][Fe(dca)$_3$] and **(b)** a_g[TPnA][Fe(dca)$_3$]. **(a)** Crystals were first heated above T_m at a rate of 10 °C min^{-1} under an argon atmosphere and then cooled to -70 °C at a rate of 5 °C min^{-1}. **(b)** The glass formed after quenching was then reheated at a rate of 10 °C min^{-1} to obtain the glass transition (T_g) which was identified (shown within black dashed line box) and marked with asterisk (zoomed and shown in inset). The glass also found to have cold crystallised and then melts at higher temperature (represented with sections under grey boxes).

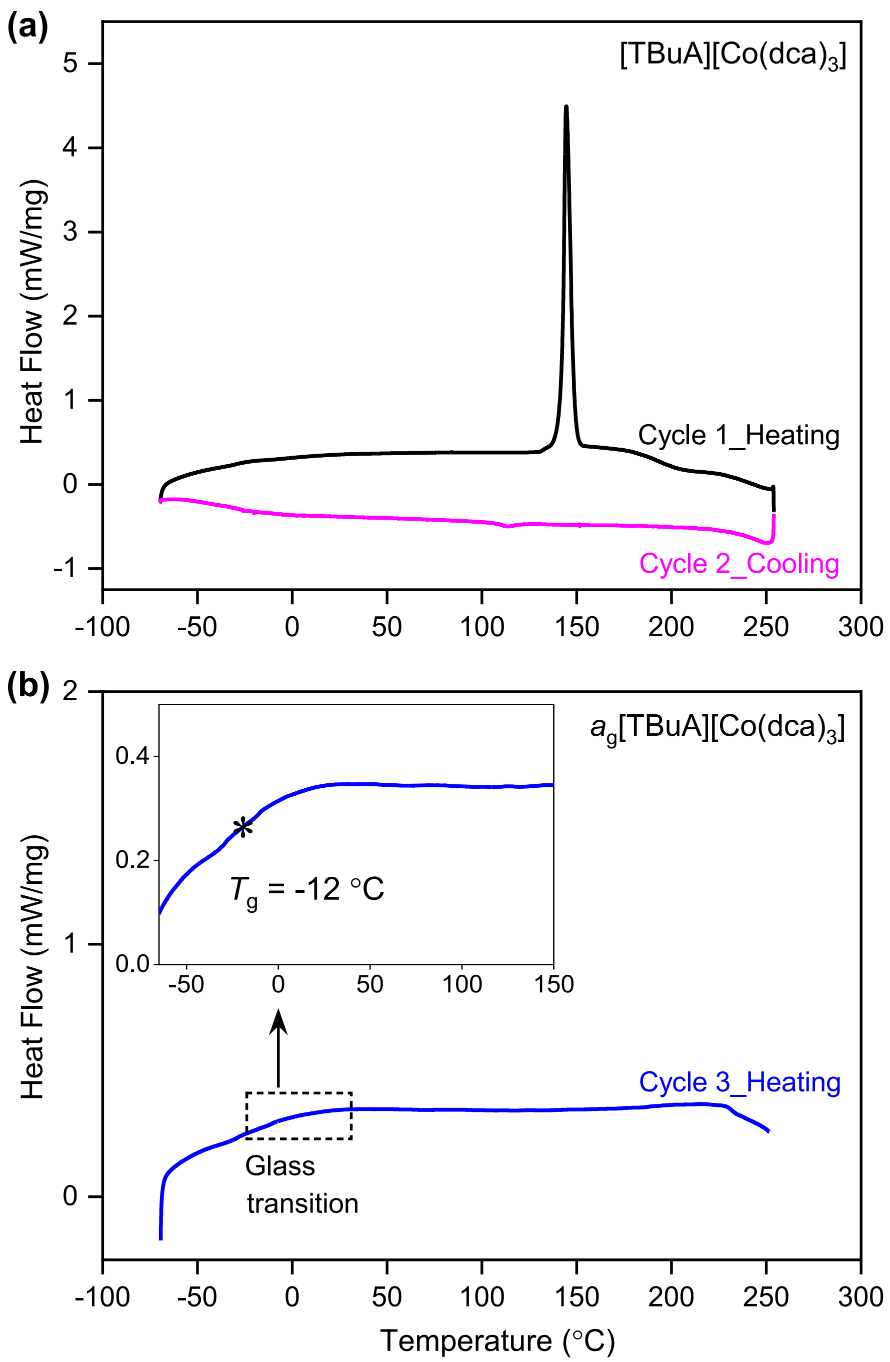

Figure S28: Change in heat flow as a function of temperature for **(a)** [TBuA][Co(dca)$_3$] and **(b)** a_g[TBuA][Co(dca)$_3$]. **(a)** Crystals were first heated above T_m at a rate of 10 °C min^{-1} under an argon atmosphere and then cooled to -70 °C at a rate of 5 °C min^{-1}. **(b)** The glass formed after quenching was then reheated at a rate of 10 °C min^{-1} to obtain the glass transition (T_g) which was identified (shown within black dashed line box) and marked with asterisk (zoomed and shown in inset).

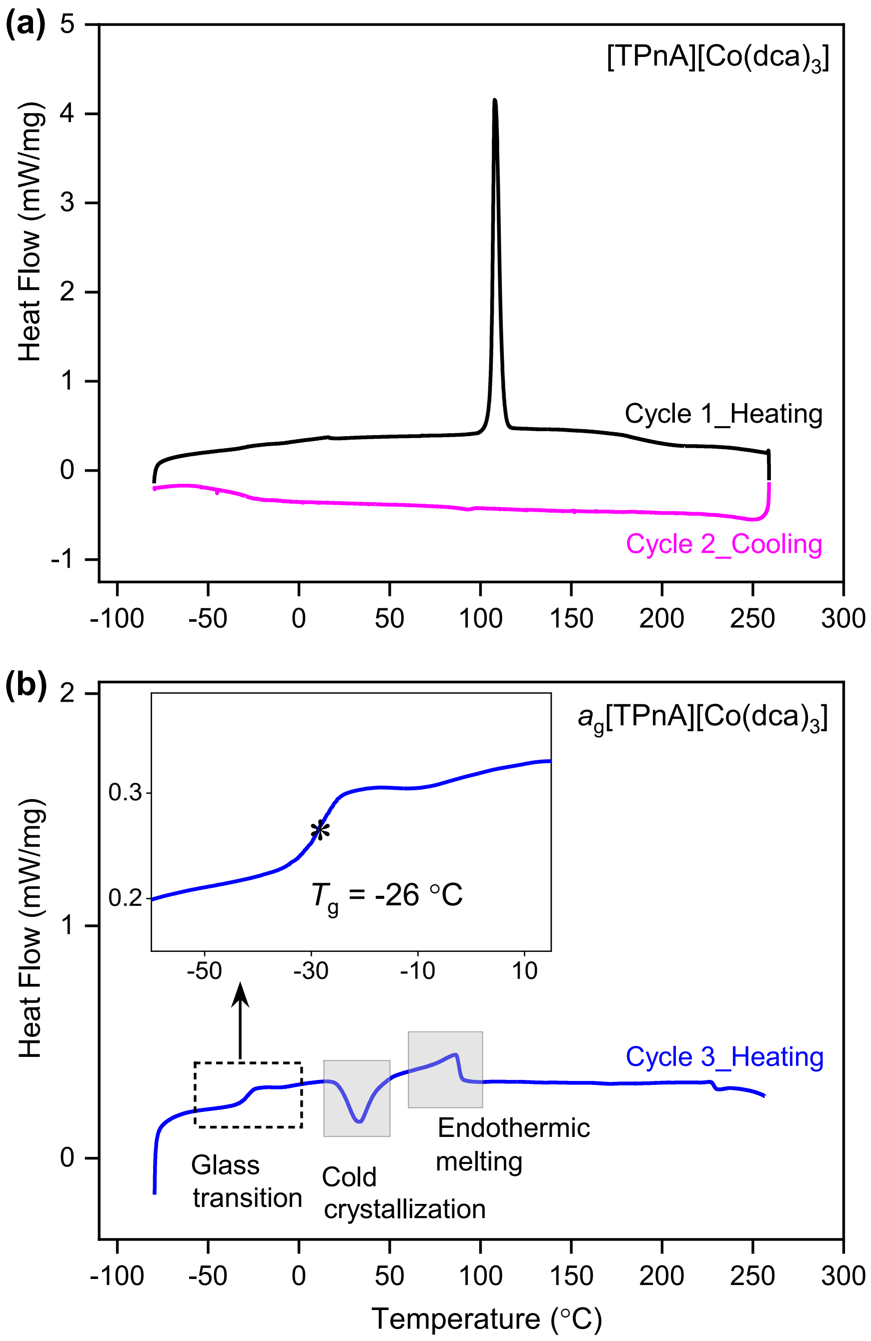

Figure S29: Change in heat flow as a function of temperature for **(a)** [TPnA][Co(dca)$_3$] and **(b)** a_g[TPnA][Co(dca)$_3$]. **(a)** Crystals were first heated above T_m at a rate of 10 °C min^{-1} under an argon atmosphere and then cooled to -80 °C at a rate of 5 °C min^{-1}. **(b)** The glass formed after quenching was then reheated at a rate of 10 °C min^{-1} to obtain the glass transition (T_g) which was identified (shown within black dashed line box) and marked with asterisk (zoomed and shown in inset). The glass also found to have cold crystallised and then melts at higher temperature (represented with sections under grey boxes).

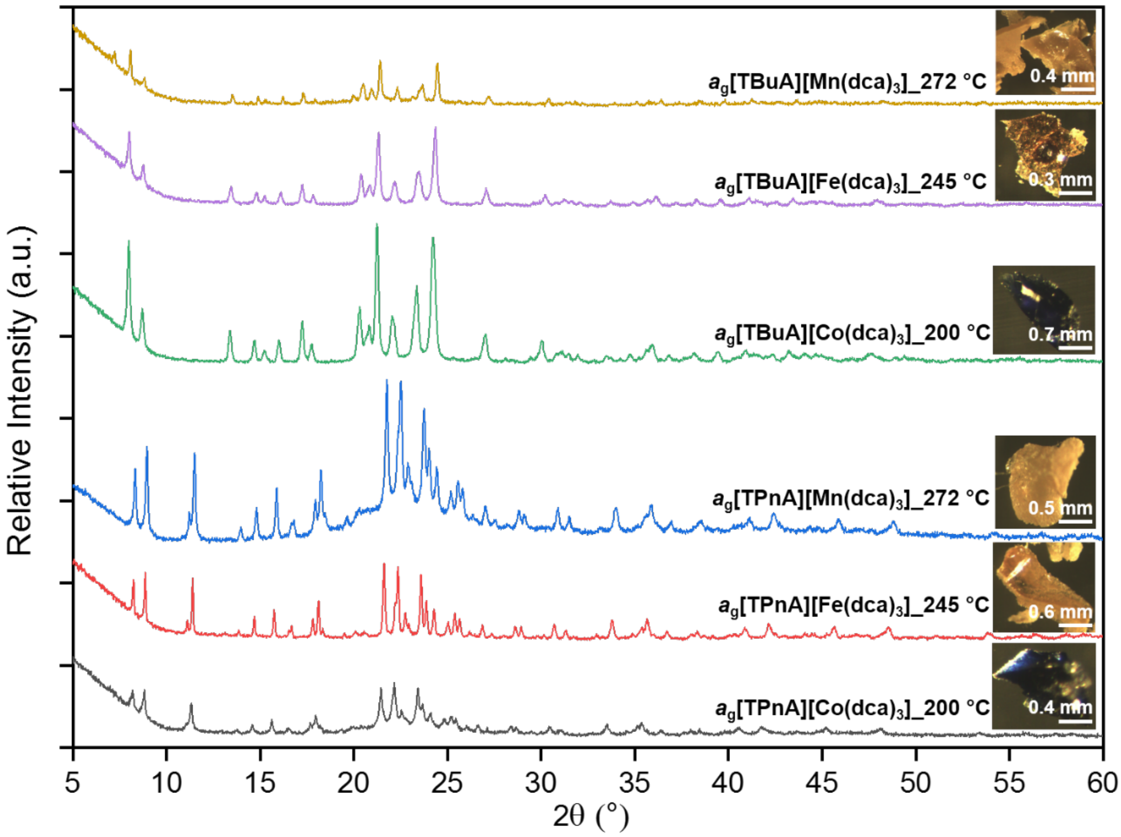

Figure S30. Ambient temperature X-ray diffraction patterns of samples show partial recrystallisation in a_g[TBuA][M(dca)$_3$] and a_g[TPnA][M(dca)$_3$] obtained by heating the samples to 272 °C (Mn), 245 °C (Fe) and 200 °C (Co) respectively, before cooling back to room temperature. Inset shows the optical images of the corresponding materials.

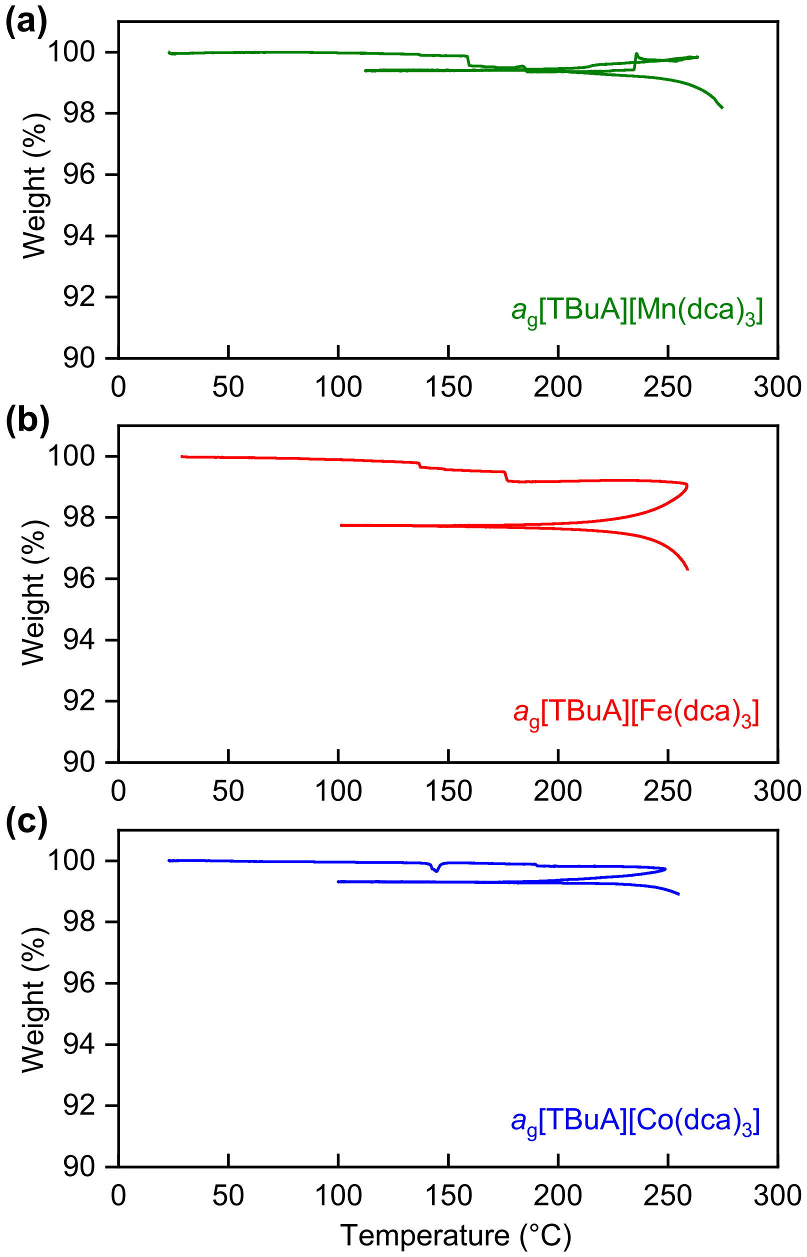

Figure S31. Values of gravimetric weight change (%) with temperature for two consecutive heating upscans for a_g[TBuA][Mn(dca)$_3$] (green), a_g[TBuA][Fe(dca)$_3$] (red) and a_g[TBuA][Co(dca)$_3$] (blue) at a heating/cooling rate ca. 10 °C min^{-1} under an argon atmosphere. Temperature profile of the scans were above the melting offset (T_m) identified in each case, before returning to low temperature and then heating again above the melting temperature identified previously.

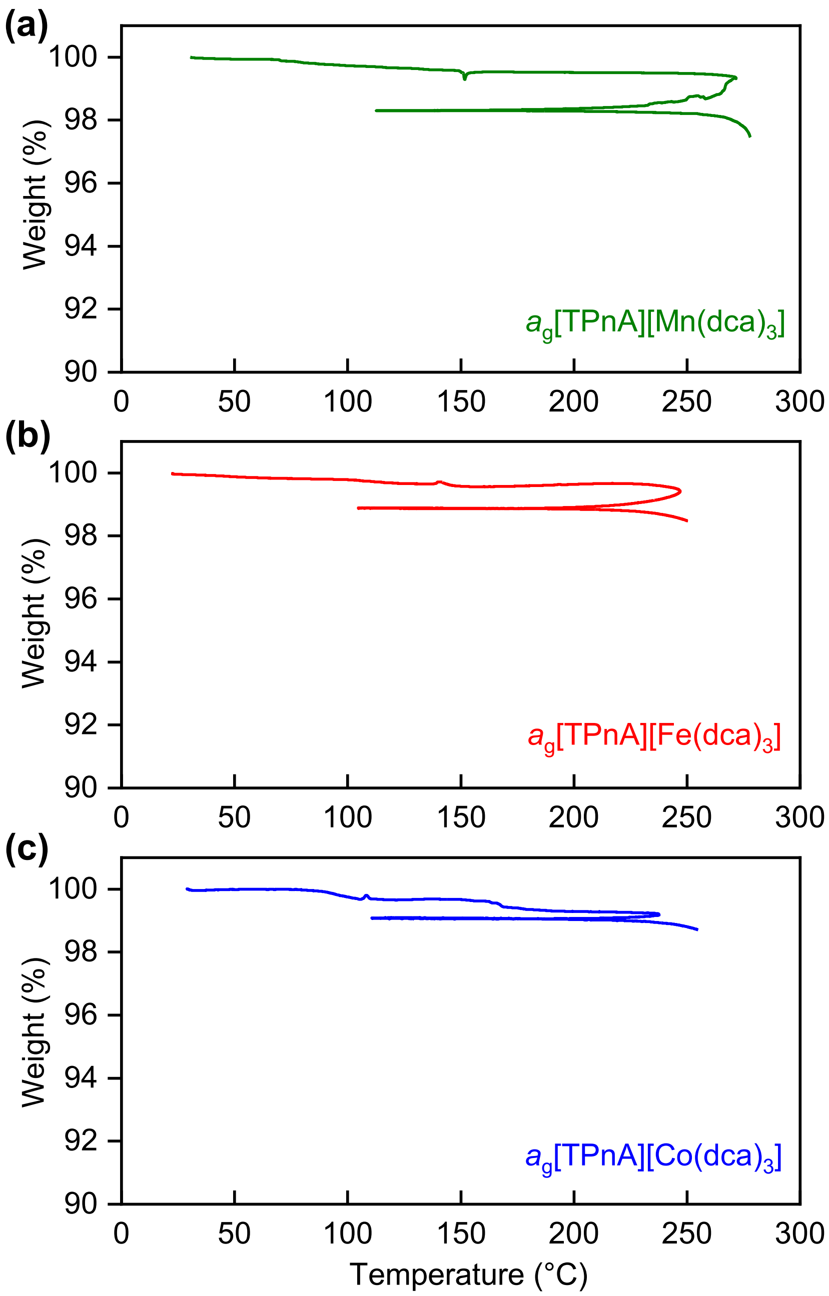

Figure S32. Values of gravimetric weight change (%) with temperature for two consecutive heating upscans for a_g[TPnA][Mn(dca)$_3$] (green), a_g[TPnA][Fe(dca)$_3$] (red) and a_g[TPnA][Co(dca)$_3$] (blue) at a heating/cooling rate ca. 10 °C min^{-1} under an argon atmosphere. Temperature profile of the scans were above the melting offset (T_m) identified in each case, before returning to low temperature and then heating again above the melting temperature identified previously.

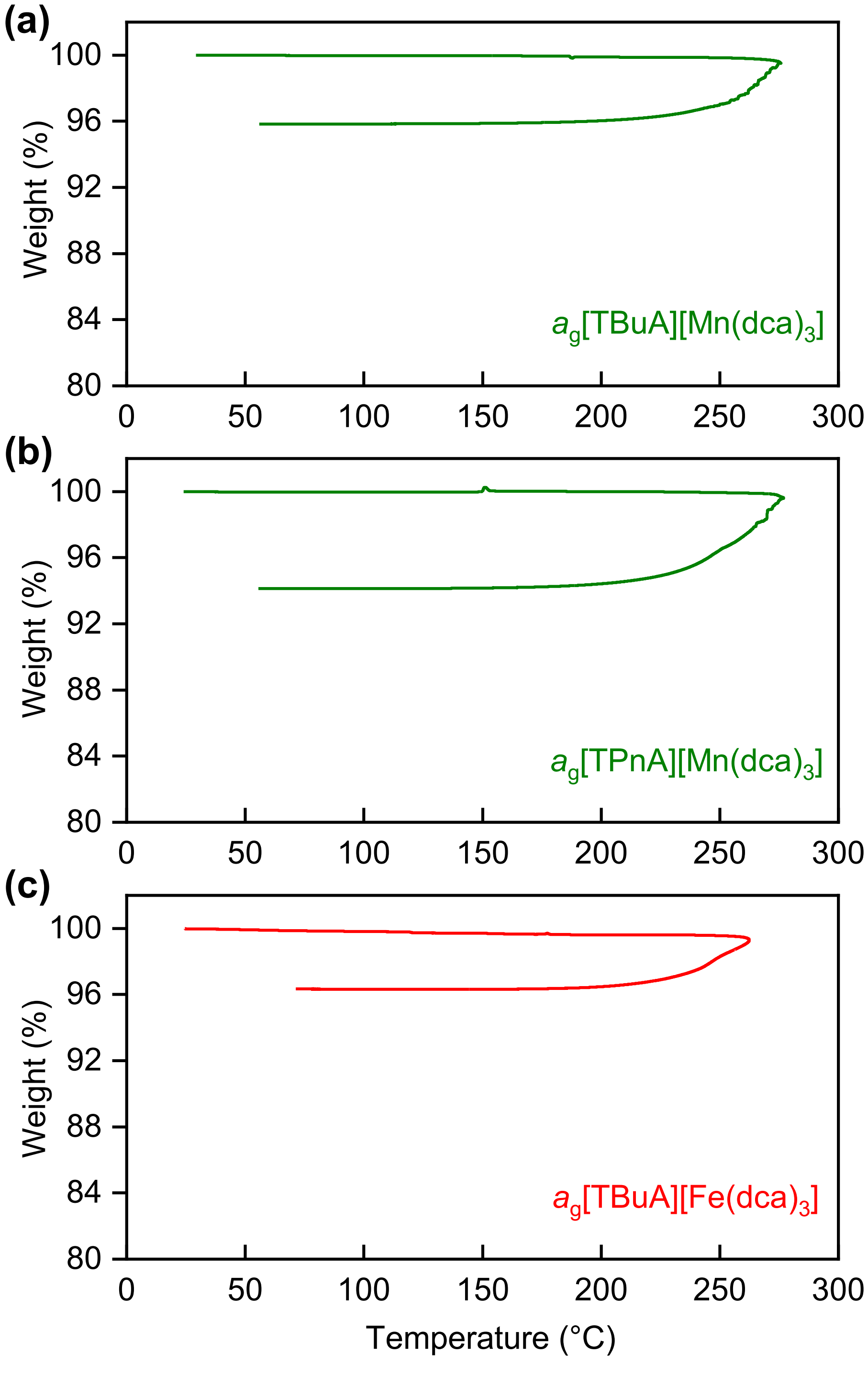

Figure S33. Values of gravimetric weight change (%) with temperature for two consecutive heating upscans at a heating/cooling rate ca. 10 °C min^{-1}/3 °C min^{-1} for a_g[TBuA][Mn(dca)$_3$] (green) and a_g[TPnA][Mn(dca)$_3$] (green), and ca. 10 °C min^{-1}/5 °C min^{-1} for a_g[TBuA][Fe(dca)$_3$] (red) under an argon atmosphere. Temperature profile of the scans were above the melting offset (T_m) identified in each case, before returning to low temperature and then heating again above the melting temperature identified previously.

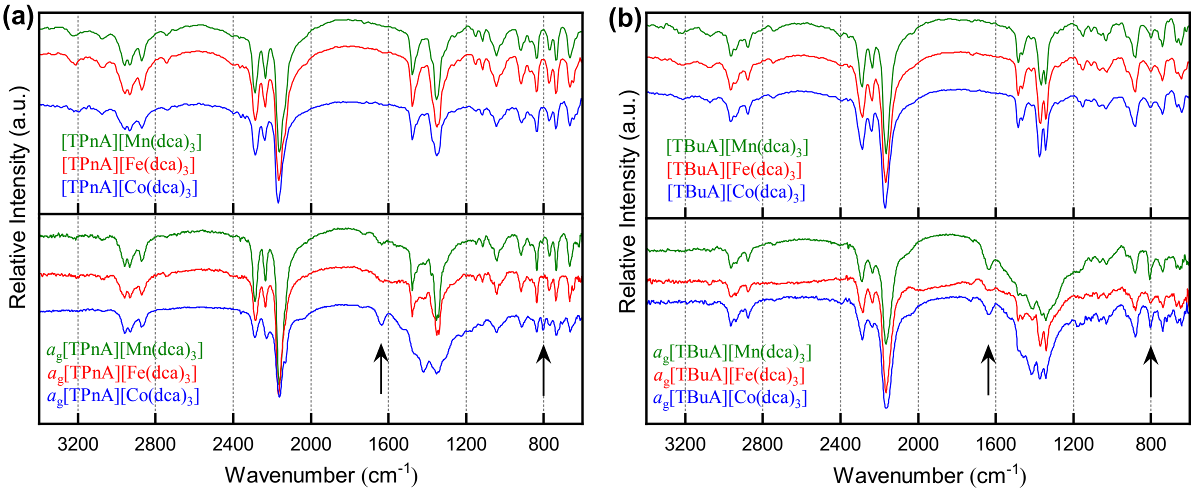

Figure S34. FT-IR spectra of **(a)** [TPnA][M(dca)$_3$], (**b)** [TBuA][M(dca)$_3$], crystals (top) and glasses (bottom). Small bands appeared at 1629-1634 cm^{-1} and 802-806 cm^{-1} regions (indicated by black arrow) in all a_g[TAlA][M(dca)$_3$] samples indicates high temperature deformation of a portion of dca ligand (vibration of $\delta_{C\text{-}N\text{-}C}$).[7–10]

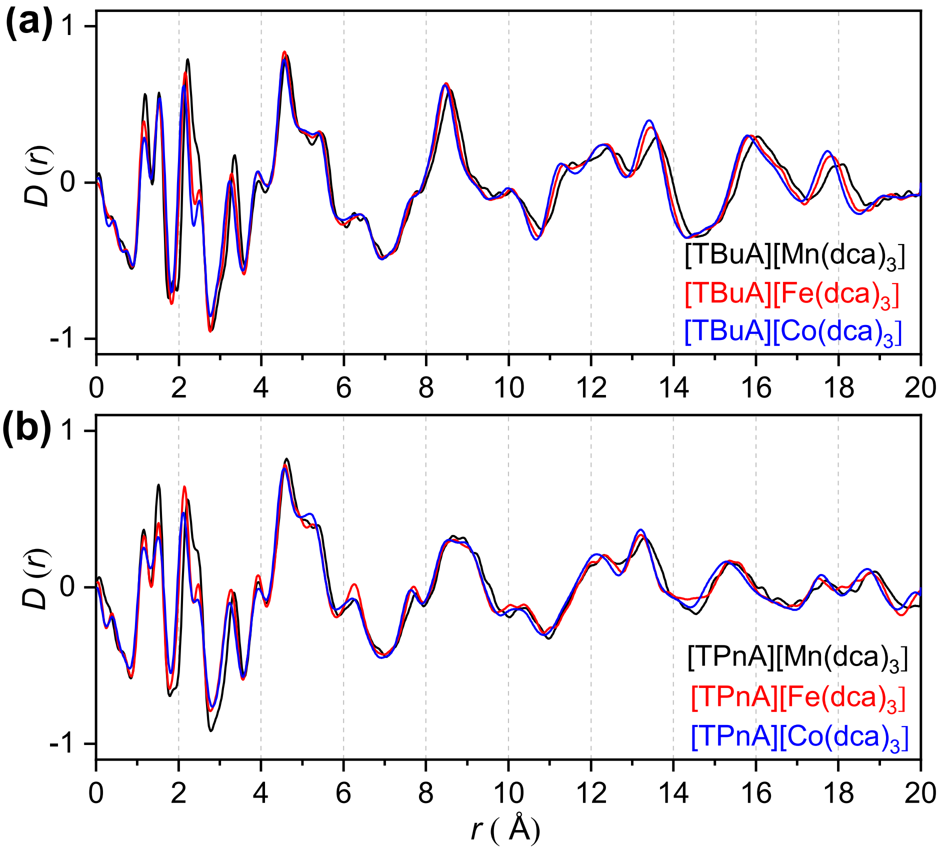

Figure S35. Pair distribution functions, $D(r)$ of **(a)** [TBuA][M(dca)$_3$] and **(b)** [TPnA][M(dca)$_3$] (M = Mn, Fe, Co).

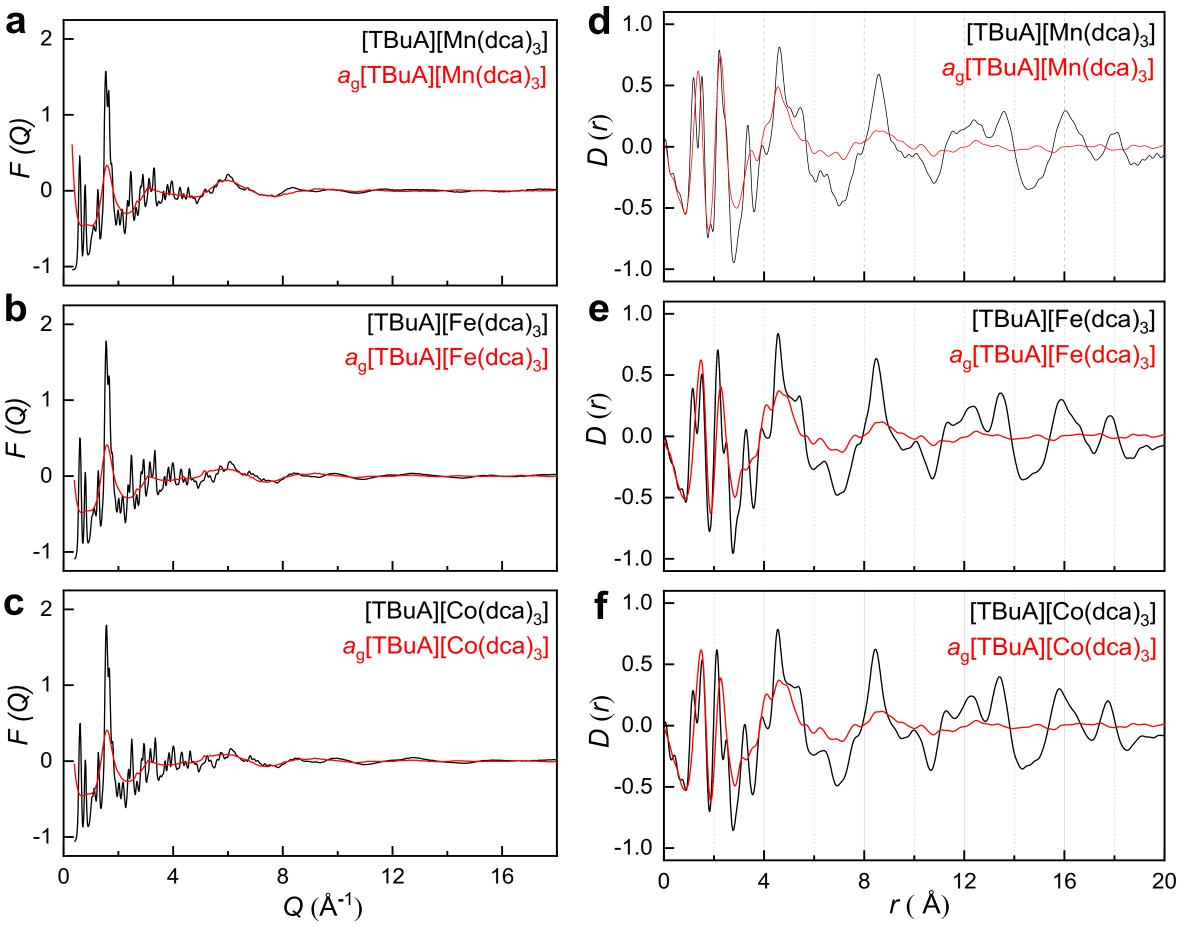

Figure S36. X-ray total scattering data of [TBuA][M(dca)$_3$] (M = Mn, Fe, Co). **(a-c)** Structure factors, $F(Q)$ and corresponding **(d-f)** Pair distribution functions, $D(r)$ before heating (black - crystal) and upon quenching from the liquid phase (red - glass).

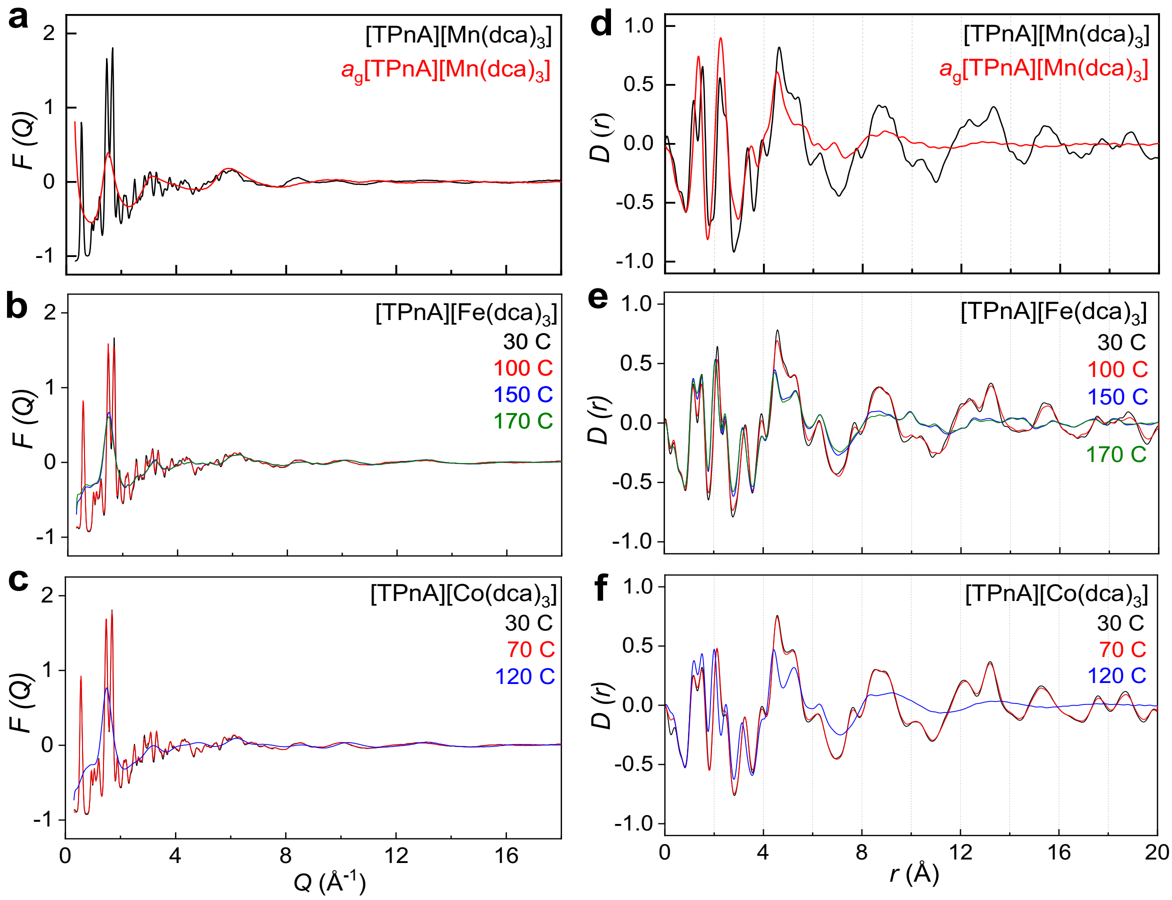

Figure S37. X-ray total scattering data of [TPnA][M(dca)$_3$] (M = Mn, Fe, Co). **(a)** Structure factors, *F*(*Q*) and corresponding **(d)** Pair distribution functions, *D*(*r*) of [TPnA][Mn(dca)$_3$] before heating (black - crystal) and upon quenching from the liquid phase (red - glass). **(b-c)** Structure factors, *F*(*Q*) and corresponding **(e-f)** Pair distribution functions, *D*(*r*) of [TPnA][Fe(dca)$_3$] and [TPnA][Co(dca)$_3$] with the variation of temperature.

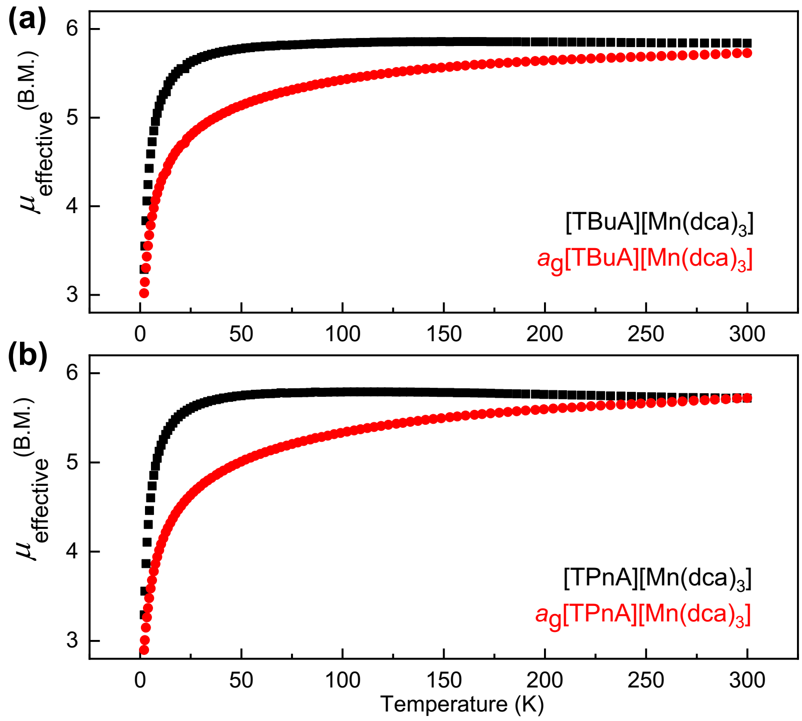

Figure S38. Variation of magnetic susceptibility in the form of effective magnetic moment (μ_{eff}) vs. temperature for [TBuA][Mn(dca)$_3$] (top) and [TPnA][Mn(dca)$_3$] (bottom) crystal (black) and glass (red). A 500 Oe magnetic field is applied to measure the field-cooled susceptibility data.

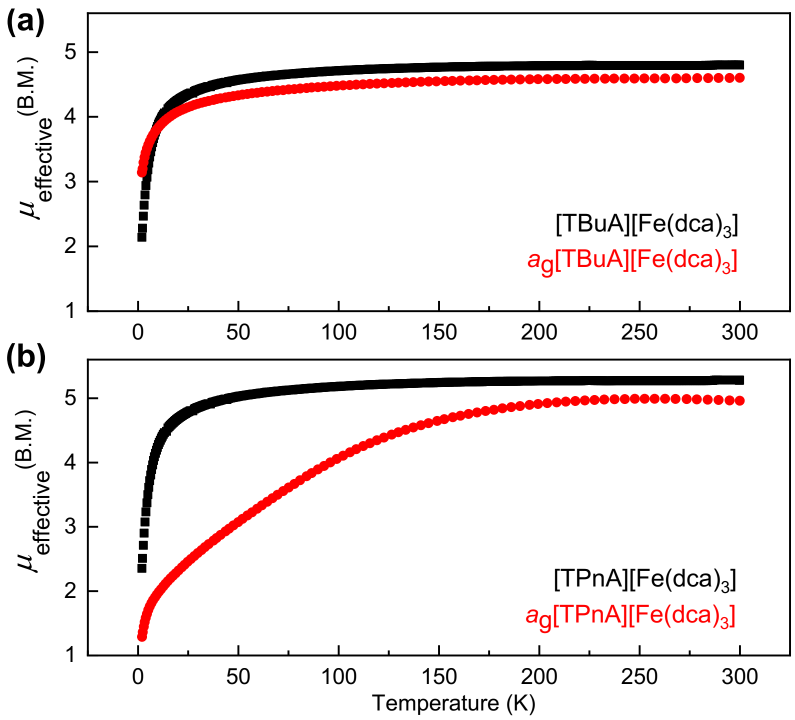

Figure S39. Variation of magnetic susceptibility in the form of effective magnetic moment (μ_{eff}) vs. temperature for [TBuA][Fe(dca)$_3$] (top) and [TPnA][Fe(dca)$_3$] (bottom) crystal (black) and glass (red). A 500 Oe magnetic field is applied to measure the field-cooled susceptibility data.

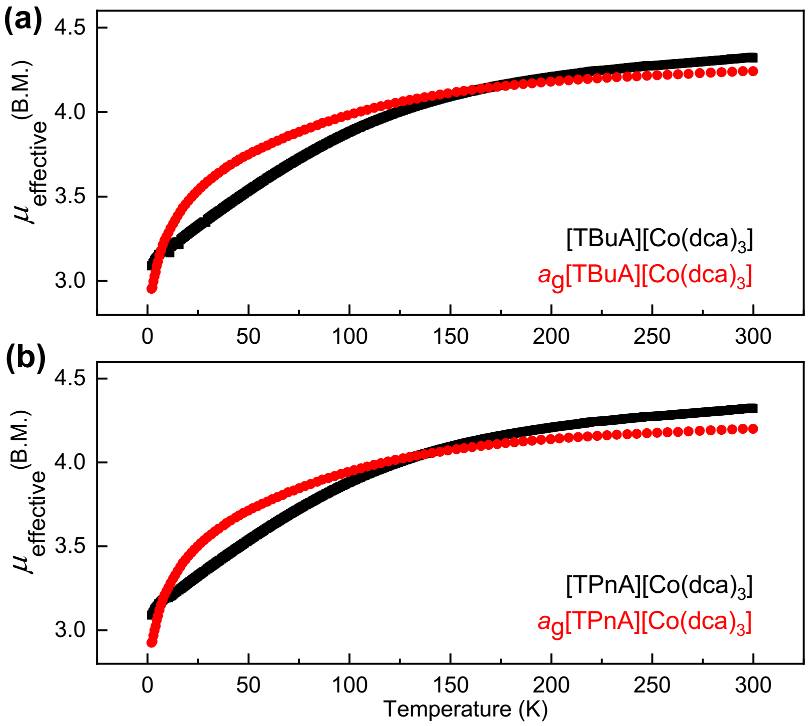

Figure S40. Variation of magnetic susceptibility in the form of effective magnetic moment (μ_{eff}) vs. temperature for [TBuA][Co(dca)$_3$] (top) and [TPnA][Co(dca)$_3$] (bottom) crystal (black) and glass (red). A 500 Oe magnetic field is applied to measure the field-cooled susceptibility data.

Table S5. Values of the enthalpy of fusion (ΔH_f) and the entropy of fusion (ΔS_f) obtained after integrating the melting peaks both with or without the negative baseline for [TPrA][Fe(dca)$_3$] and [TPrA][Co(dca)$_3$] compounds.

**Samples**	**ΔH_f (without baseline) (kJ mol^{-1})**	**ΔS_f (without baseline) (J mol^{-1} K^{-1})**	**ΔH_f (with baseline) (kJ mol^{-1})**	**ΔS_f (with baseline) (J mol^{-1} K^{-1})**	**Ref**
[TPrA][Fe(dca)$_3$]	35	67	50 [b]	95 [b]	10
[TPrA][Co(dca)$_3$]	49	101	65 [b]	134 [b]	10

[b] We have preferred the values in the manuscript obtained after integrating the peaks up to the melting offset temperature which defines the whole region of melting.

Table S6. Pycnometric densities of hybrid organic-inorganic ABX_3 structures in their crystalline and glassy (a_g) states.

Samples	ρ_c (g cm^{-3})	ρ_g (g cm^{-3})
[TBuA][Mn(dca)$_3$]	1.195 (±0.005)	1.235 (±0.006)
[TBuA][Fe(dca)$_3$]	1.270 (±0.004)	1.316 (±0.002)
[TBuA][Co(dca)$_3$]	1.234 (±0.005)	1.283 (±0.004)
[TPnA][Mn(dca)$_3$]	1.205 (±0.005)	1.265 (±0.006)
[TPnA][Fe(dca)$_3$]	1.211 (±0.004)	1.305 (±0.004)
[TPnA][Co(dca)$_3$]	1.217 (±0.005)	1.335 (±0.003)

Table S7. Values of FWHM, d_0 and the peak area obtained after analyzing the Mn-N pair distribution function (PDF) peak trajectories with the variation of temperature (Figure S15 and S19).

[TBuA][Mn(dca)$_3$]

**34C**	C-N (Grey)	C-C (Cyan)	C-N (Pink)	Mn-N (Blue)	C-C (Orange)	C-N (Green)
Position	1.187	1.527	1.860	2.200	2.471	2.591
Area	0.376	0.412	0.126	0.651	0.175	0.612
FWHM	0.264	0.251	0.204	0.297	0.226	0.517

**114C**	C-N (Grey)	C-C (Cyan)	C-N (Pink)	Mn-N (Blue)	C-C (Orange)	C-N (Green)
Position	1.185	1.532	1.851	2.201	2.461	2.579
Area	0.415	0.466	0.148	0.711	0.139	0.674
FWHM	0.278	0.265	0.215	0.305	0.198	0.447

**145C**	C-N (Grey)	C-C (Cyan)	C-N (Pink)	Mn-N (Blue)	C-C (Orange)	C-N (Green)
Position	1.182	1.536	1.860	2.204	2.456	2.547
Area	0.373	0.431	0.089	0.587	0.121	0.438
FWHM	0.278	0.266	0.200	0.310	0.198	0.383

**169C**	C-N (Grey)	C-C (Cyan)	C-N (Pink)	Mn-N (Blue)	C-C (Orange)	C-N (Green)
Position	1.176	1.536	1.856	2.205	2.470	2.570
Area	0.369	0.446	0.070	0.606	0.114	0.484
FWHM	0.293	0.296	0.195	0.329	0.200	0.460

**190C**	C-N (Grey)	C-C (Cyan)	C-N	Mn-N (Blue)	C-C (Orange)	C-N (Green)
Position	1.155	1.523	1.856	2.140	2.477	2.589
Area	0.317	0.549	0.070	0.610	0.133	0.552
FWHM	0.301	0.402	0.195	0.414	0.288	0.581

211C	C-N (Grey)	C-C (Cyan)	C-N	Mn-N (Blue)	C-C (Orange)	C-N (Green)
Position	1.150	1.524	1.856	2.117	2.460	2.586
Area	0.329	0.681	0.070	0.687	0.165	0.931
FWHM	0.304	0.441	0.195	0.413	0.344	0.638

236C	C-N (Grey)	C-C (Cyan)	C-N	Mn-N (Blue)	C-C (Orange)	C-N (Green)
Position	1.152	1.526	1.856	2.110	2.469	2.562
Area	0.341	0.642	0.070	0.644	0.284	0.665
FWHM	0.312	0.433	0.195	0.425	0.429	0.555

258C	C-N (Grey)	C-C (Cyan)	C-N	Mn-N (Blue)	C-C (Orange)	C-N (Green)
Position	1.159	1.526	1.856	2.119	2.480	2.589
Area	0.350	0.606	0.070	0.695	0.181	0.752
FWHM	0.318	0.412	0.195	0.430	0.340	0.611

[TPnA][Mn(dca)$_3$]

34C	C-N (Grey)	C-C (Cyan)	C-N (Pink)	Mn-N (Blue)	C-C (Orange)	C-N (Green)
Position	1.162	1.527	1.870	2.202	2.490	2.579
Area	0.406	0.564	0.174	0.703	0.187	0.702
FWHM	0.307	0.292	0.247	0.328	0.256	0.476

113C	C-N (Grey)	C-C (Cyan)	C-N (Pink)	Mn-N (Blue)	C-C (Orange)	C-N (Green)
Position	1.164	1.539	1.867	2.195	2.478	2.593
Area	0.451	0.625	0.188	0.786	0.244	0.745
FWHM	0.336	0.308	0.233	0.345	0.265	0.458

145C	C-N (Grey)	C-C (Cyan)	C-N (Pink)	Mn-N (Blue)	C-C (Orange)	C-N (Green)
Position	1.164	1.532	1.858	2.169	2.475	2.542
Area	0.427	0.668	0.196	0.829	0.304	0.819
FWHM	0.313	0.331	0.242	0.361	0.334	0.439

169C	C-N (Grey)	C-C (Cyan)	C-N	Mn-N (Blue)	C-C (Orange)	C-N (Green)
Position	1.094	1.496	1.858	2.119	2.450	2.556
Area	0.225	0.824	0.196	0.667	0.157	0.683
FWHM	0.270	0.548	0.242	0.394	0.279	0.477

190C	C-N (Grey)	C-C (Cyan)	C-N	Mn-N (Blue)	C-C (Orange)	C-N (Green)
Position	1.137	1.493	1.858	2.122	2.463	2.596
Area	0.312	0.621	0.196	0.737	0.200	0.609
FWHM	0.288	0.420	0.242	0.416	0.332	0.513

215C	C-N (Grey)	C-C (Cyan)	C-N	Mn-N (Blue)	C-C (Orange)	C-N (Green)

Position	1.132	1.489	1.858	2.101	2.468	2.585
Area	0.319	0.683	0.196	0.716	0.241	0.801
FWHM	0.292	0.439	0.242	0.410	0.375	0.607

**237C**	C-N (Grey)	C-C (Cyan)	C-N	Mn-N (Blue)	C-C (Orange)	C-N (Green)
Position	1.144	1.495	1.858	2.102	2.478	2.595
Area	0.357	0.632	0.196	0.768	0.175	0.554
FWHM	0.304	0.401	0.242	0.421	0.292	0.450

**257C**	C-N (Grey)	C-C (Cyan)	C-N	Mn-N (Blue)	C-C (Orange)	C-N (Green)
Position	1.124	1.470	1.858	2.102	2.473	2.530
Area	0.268	0.753	0.196	0.789	0.211	0.642
FWHM	0.284	0.463	0.242	0.426	0.374	0.477

Table S8. Elemental composition (C, H, N) of hybrid organic-inorganic ABX_3 structures in their crystalline and glassy (a_g) states.

**Samples**	**C - %**	**H - %**	**N - %**	**C-a_g - %**	**H-a_g - %**	**N-a_g - %**
[TBuA][Mn(dca)$_3$]	53.16 (53.44)	7.60 (7.22)	27.39 (28.30)	51.18	7.05	27.39
[TBuA][Fe(dca)$_3$]	53.13 (53.40)	7.35 (7.29)	27.22 (28.14)	51.98	7.27	26.70
[TBuA][Co(dca)$_3$]	52.89 (53.25)	7.37 (7.39)	26.56 (27.80)	51.92	6.94	26.56
[TPnA][Mn(dca)$_3$]	57.10 (57.22)	8.78 (8.88)	24.33 (25.45)	56.57	8.70	23.46
[TPnA][Fe(dca)$_3$]	56.96 (57.12)	8.49 (8.78)	24.10 (25.22)	56.82	8.31	23.71
[TPnA][Co(dca)$_3$]	56.27 (57.10)	8.15 (8.44)	24.54 (25.05)	55.36	8.01	24.55

* Expected elemental compositions were shown in first bracket.

References

1. Schlueter, J. A., Manson, J. L. & Geiser, U. Structural and Magnetic Diversity in Tetraalkylammonium Salts of Anionic M [N(CN) 2] 3 - (M = Mn and Ni) Three-Dimensional Coordination Polymers. *Inorg. Chem.* **44**, 3194–3202 (2005).

2. Dolomanov, O. V., Bourhis, L. J., Gildea, R. J., Howard, J. A. K. & Puschmann, H. OLEX2 : a complete structure solution, refinement and analysis program. *J. Appl. Crystallogr.* **42**, 339–341 (2009).

3. Sheldrick, G. M. A short history of SHELX. *Acta Crystallogr. Sect. A Found. Crystallogr.* **64**, 112–122 (2008).

4. Palatinus, L. & Chapuis, G. SUPERFLIP – a computer program for the solution of crystal structures by charge flipping in arbitrary dimensions. *J. Appl. Crystallogr.* **40**, 786–790 (2007).

5. Coelho, A. A. TOPAS and TOPAS-Academic : an optimization program integrating computer algebra and crystallographic objects written in C++. *J. Appl. Crystallogr.* **51**, 210–218 (2018).

6. Farrow, C. L. *et al.* PDFfit2 and PDFgui: computer programs for studying nanostructure in crystals. *J. Phys. Condens. Matter* **19**, 335219 (2007).

7. Bermúdez-García, J. M. *et al.* A simple in situ synthesis of magnetic M@CNTs by thermolysis of the hybrid perovskite [TPrA][M(dca) 3]. *New J. Chem.* **41**, 3124–3133 (2017).

8. Kroke, E. *et al.* Tri-s-triazine derivatives. Part I. From trichloro-tri-s-triazine to graphitic C3N4 structures. *New J. Chem.* **26**, 508–512 (2002).

9. Täuber, K., Dani, A. & Yuan, J. Covalent Cross-Linking of Porous Poly(ionic liquid) Membrane via a Triazine Network. *ACS Macro Lett.* **6**, 1–5 (2017).

10. Shaw, B. K., Hughes, A. R., Ducamp, M., Moss, S., Debnath, A., Sapnik, A. F., Thorne, M. F., McHugh, L. N., Keeble, D., Chater, P., Bermudez-Garcia, J. M., Moya, X., Saha, S. K., Keen, D. A., Coudert, F. X., Blanc, F. and T. D. Bennett, Melting of hybrid organic–inorganic perovskites. *Nat. Chem.* **13**, 778-785 (2021).
